# Multiple Translocation of the *AVR-Pita* Effector Gene among Chromosomes of the Rice Blast Fungus *Magnaporthe oryzae* and Related Species

**DOI:** 10.1371/journal.ppat.1002147

**Published:** 2011-07-28

**Authors:** Izumi Chuma, Chihiro Isobe, Yuma Hotta, Kana Ibaragi, Natsuru Futamata, Motoaki Kusaba, Kentaro Yoshida, Ryohei Terauchi, Yoshikatsu Fujita, Hitoshi Nakayashiki, Barbara Valent, Yukio Tosa

**Affiliations:** 1 Graduate School of Agricultural Sciences, Kobe University, Kobe, Japan; 2 Faculty of Agriculture, Saga University, Saga, Japan; 3 Iwate Biotechnology Research Center, Kitakami, Japan; 4 National Agricultural Research Center, Tsukuba, Japan; 5 Department of Plant Pathology, Kansas State University, Manhattan, Kansas, United States of America; Virginia Polytechnic Institute and State University, United States of America

## Abstract

*Magnaporthe oryzae* is the causal agent of rice blast disease, a devastating problem worldwide. This fungus has caused breakdown of resistance conferred by newly developed commercial cultivars. To address how the rice blast fungus adapts itself to new resistance genes so quickly, we examined chromosomal locations of *AVR-Pita*, a subtelomeric gene family corresponding to the *Pita* resistance gene, in various isolates of *M. oryzae* (including wheat and millet pathogens) and its related species. We found that *AVR-Pita* (*AVR-Pita1* and *AVR-Pita2*) is highly variable in its genome location, occurring in chromosomes 1, 3, 4, 5, 6, 7, and supernumerary chromosomes, particularly in rice-infecting isolates. When expressed in *M. oryzae*, most of the *AVR-Pita* homologs could elicit *Pita*-mediated resistance, even those from non-rice isolates. *AVR-Pita* was flanked by a retrotransposon, which presumably contributed to its multiple translocation across the genome. On the other hand, family member *AVR-Pita3*, which lacks avirulence activity, was stably located on chromosome 7 in a vast majority of isolates. These results suggest that the diversification in genome location of *AVR-Pita* in the rice isolates is a consequence of recognition by *Pita* in rice. We propose a model that the multiple translocation of *AVR-Pita* may be associated with its frequent loss and recovery mediated by its transfer among individuals in asexual populations. This model implies that the high mobility of *AVR-Pita* is a key mechanism accounting for the rapid adaptation toward *Pita*. Dynamic adaptation of some fungal plant pathogens may be achieved by deletion and recovery of avirulence genes using a population as a unit of adaptation.

## Author Summary

Rice blast disease, caused by *Magnaporthe oryzae*, is a serious threat to global rice production. Dozens of resistance genes are available for disease control, but the fungus rapidly evolves to overcome a resistance gene within 2 or 3 years in the field. Resistance requires recognition of corresponding pathogen “avirulence effectors”, proteins or small molecules secreted by the fungus in the host plant. Resistance gene breakdown involves mutation of the avirulence effector so that it is no longer recognized by the resistance gene product. We describe evolutionary processes associated with the subtelomeric avirulence effector *AVR-Pita* corresponding to rice resistance gene *Pita*. Comparing individuals in the asexual fungal population infecting rice, *AVR-Pita* has frequently been translocated to different chromosomes, including unstable supernumerary chromosomes. *AVR-Pita* occurs at different chromosomal localizations in populations from weeds and millet crops lacking *Pita*, but this location is stable in individuals of each population. Family member *AVR-Pita3*, which lacks a corresponding rice resistance gene, shows extremely stable localization on a single chromosome throughout all host-adapted populations of the pathogen. Translocation of *AVR-Pita* might be associated with the frequent transfer of this gene between individuals in the asexual pathogen population responding to selection by sporadic deployment of *Pita*.

## Introduction

Breeding for disease resistance is a cost-effective, labor-saving, and environmentally sound crop protection strategy. Cultivar resistance [1] conferred by major genes has been extensively used for breeding in many crop species. However, newly developed resistant cultivars have frequently been rendered ineffective within a few years after their release to farmer's fields [2], [3]. This breakdown of resistance has been caused by rapid adaptation of the pathogen, that is, evolution of new races that overcome the introduced resistance genes. The wide cultivation of a new cultivar with a major resistance gene and its breakdown by a new race has been called the boom-and-bust cycle [4]. The primary question is how plant pathogens adapt themselves to new resistance genes so quickly.

The rapid evolution of new races is attributed to rapid loss of function of avirulence effector genes that correspond to the resistance genes in a gene-for-gene manner [5]. Loss of the avirulence function can be due to point mutations, including repeat-induced point mutations [6], [7], insertions of transposable elements, or deletions of entire genes [8]. Extensive surveys of large natural populations of some fungal pathogens revealed that deletion of avirulence genes is a common mechanism of evolution towards virulence [7], [9], [10]. Then, the second question arises: how is a system involving continuous gene loss being sustained? Fungal populations often regain expelled avirulence genes after varieties containing the corresponding resistance genes are removed from the field [e.g. 11]. A simple explanation for the recovery is that isolates carrying the avirulence gene had survived as a minor race in the field during the prevalent cultivation of a resistant cultivar carrying its corresponding resistance gene. Then, when planting of the resistant cultivar came to a halt, their population increased again owing to the contribution of the avirulence gene to fitness. An alternative explanation is that isolates carrying the avirulence gene migrated from regions where the resistance gene had not been deployed. Sexual recombination would allow isolates that have undergone deletion of an avirulence gene to regain that gene, but pathogens such as the rice blast fungus are predominantly asexual in the field. It is of interest to understand if asexually-reproducing pathogen populations have a mechanism to regain deleted avirulence genes.

The genus *Pyricularia* is the causal agent of blast diseases of monocot species. This genus includes several morphological species such as *P. higginsii*, *P. zingiberi*, *P. zizaniaecola*, and the *P. grisea/oryzae* species complex [12]. The *P. grisea/oryzae* species complex is then composed of some cryptic species such as *Pyricularia* sp. (CE) pathogenic on *Cenchrus* spp. [12], *P. grisea* pathogenic on *Digitaria* spp. (including crabgrass) [13], and *P. oryzae* [13]. The most familiar species, *P. oryzae*, is the anamorph (asexual reproductive stage) of *Magnaporthe oryzae* [14], and is composed of several host-specific subgroups such as *Oryza* isolates pathogenic on *Oryza* spp. (including rice, *O. sativa*), *Setaria* isolates pathogenic on *Setaria* spp. (including foxtail millet, *S. italica*), *Triticum* isolates pathogenic on *Triticum* spp. (including common wheat, *T. aestivum*), *Panicum* isolates pathogenic on *Panicum* spp. (including common millet, *P. miliaceum*), etc. [13], [15]. Finally, *Oryza* isolates and *Setaria* isolates include various races which show different patterns of avirulence on cultivars of rice and foxtail millet, respectively [16]. Extensive population analyses of mating type distribution, sexual fertility, and genotypic diversity indicate that *Oryza* isolates responsible for rice blast disease are limited to asexual reproduction in most areas of the world [17]. The *P. oryzae* teleomorph can be produced on artificial media [18]–[20], but has not been found in natural fields.

Both subgroup - genus specificity (a type of traditional “host species specificity”) in *P. oryzae* and race - cultivar specificity in *Oryza* isolates are governed by gene-for-gene interactions [21]–[24]. Three avirulence genes have been cloned which are involved in host species specificity; *PWL1* [25], *PWL2* [26], and *AVR1-CO39* [27]. Cloned avirulence effector genes involved in rice cultivar specificity are *AVR-Pita* [28], *ACE1* [29], *AvrPiz-t* [30], *AVR-Pia* [31], [32], *AVR-Pii* [32], and *AVR-Pik/km/kp* [32]. As with other host-pathogen systems, deletion of *P. oryzae* avirulence genes is a common mechanism for the pathogen to overcome resistance genes deployed in the host. Indeed, cloning of *AVR-Pita* [28], *PWL2* [26] and *AVR-Pia* [31] was facilitated by isolation of spontaneous virulent mutants with deletions of the avirulence genes. Similarly, association of avirulence phenotypes in field isolates with presence of candidate avirulence genes facilitated cloning of *AVR-Pia*, *AVR-Pii*, and *AVR-Pik/km/kp* [32]. Several studies confirmed that deletion of *AVR-Pita* is a common mechanism for overcoming resistance in the field, although point mutations and insertions of transposable elements also lead to virulence [28], [33]–[36].

*AVR-Pita*, which corresponds to the rice resistance gene *Pita*, is a subtelomeric gene (located adjacent to a telomere on chromosome 3), and it undergoes frequent spontaneous mutation in laboratory studies [28]. *AVR-Pita* encodes a protein with features typical of zinc metalloproteases, and the putative mature protease is predicted to bind directly to the cognate Pita protein [37] inside a plant cell to initiate hypersensitive resistance [38]. Recently, Khang et al. [39] showed that *AVR-Pita* is a member of a gene family, and they renamed it *AVR-Pita1*. Two additional family members named *AVR-Pita2* and *AVR-Pita3* shared 92% and 71% DNA sequence identity, respectively, to *AVR-Pita1. AVR-Pita2* was functional as an avirulence gene, but *AVR-Pita3* was not [39].

In this report we describe the process of evolution of the *AVR-Pita* family during the course of speciation and parasitic specialization of the genus *Pyricularia* based on analysis of morphological species, cryptic species, host-specific subgroups, and races of the rice blast pathogen. We focused on chromosomal locations of the *AVR-Pita* family for two reasons. First, Orbach et al. [28] suggested that the telomeric location of an avirulence gene may facilitate rapid adaptation of the blast fungus to its host. This strategy for avoiding the host recognition seemed similar to that adopted by animal parasites such as *Trypanosoma brucei*, a causal agent of African sleeping sickness [40]–[42], and *Plasmodium falciparum*, a causal agent of malaria. In *P. falciparum*, genes involved in antigenic variation (*var*, *rif*, etc) are concentrated in subtelomeric chromosomal regions [43]. Extraordinary diversity of *var* genes, produced by telomere-mediated ectopic recombination, makes it possible for the malaria parasite to evade attack by the host immune system [44]. This recombination can be associated with movement of *var* genes to new chromosome ends [44]. Second, based on differences in stability of the *AVR-Pita* gene among different fungal strains, Orbach et al. [28] hypothesized that the gene might reside at different chromosomal locations in different strains. Here, we demonstrate that *AVR-Pita* (*AVR-Pita1* and *AVR-Pita2*) has been frequently translocated among chromosomes, particularly in *Oryza* isolates that have evolved in response to periodic deployment of *Pita* to control blast disease. We propose that “multiple translocation” of *AVR-Pita* may be associated with its frequent loss and recovery mediated by its transfer among individuals in asexual populations. This model implies that the high mobility of an avirulence gene is a key mechanism accounting for the rapid adaptation toward its corresponding resistance gene and that the system of continuous loss of an avirulence gene is sustained by its recovery from other individuals.

## Results

### *AVR-Pita* is unique to the *Pyricularia grisea/oryzae* species complex

Throughout this paper the anamorph (asexual) name “*Pyricularia*” will be used instead of the teleomorph (sexual) name “*Magnaporthe*” because fungal materials employed include several *Pyricularia* species whose sexual stages have not been recognized. To survey the distribution of *AVR-Pita* homologs in the genus *Pyricularia*, we chose 99 “core isolates” from our stock cultures that covered the entire variation of *Pyricularia* isolates we have collected. These isolates are listed in Table S1 with their code names, original hosts, place and year of isolation, etc. The code name (e.g., O-1G) is composed of its host genus (*Oryza*), a serial number within isolates from the same host genus (−1), and the country where it was collected (Guiana). The core isolates included seven “old” *Oryza* isolates (O-2J, O-3J, O-4J, O-5J, O-6J, O-7J, and O-8J) collected before 1960.

**Figure 1:**
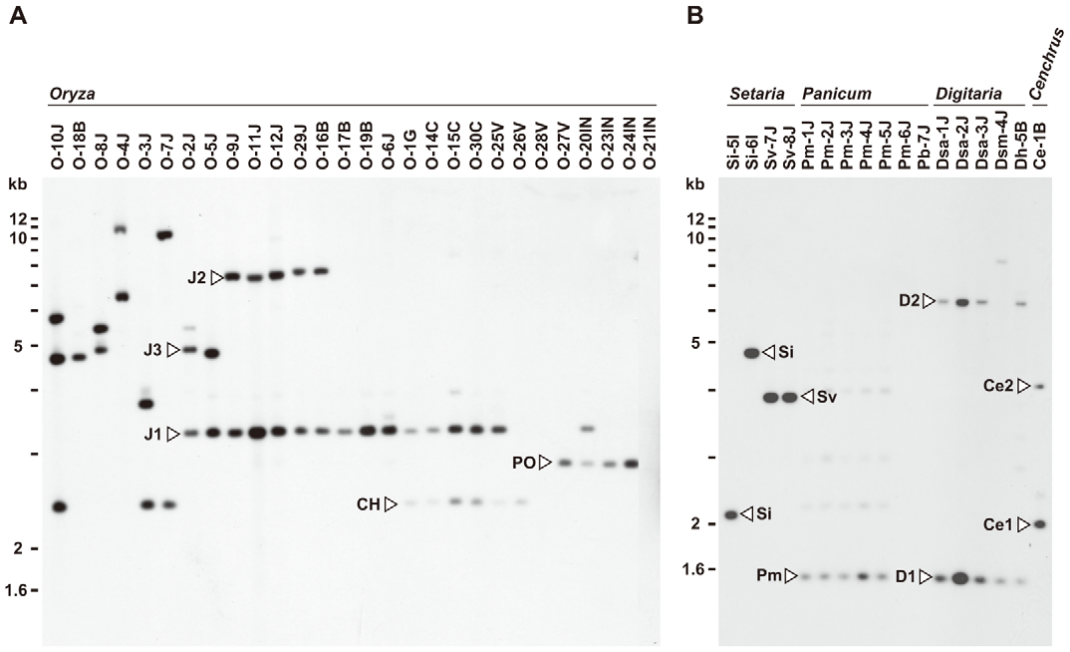
Southern blot analysis of *AVR-Pita* homologs in *Pyricularia* isolates. Genomic DNA was digested with *Acl*I and hybridized with the 766-bp *AVR-Pita* fragment (APita766). (A) RFLP types (J1, J2, J3, CH, and PO) found in *Oryza* isolates. (B) RFLP types (Si, Sv, Pm, D1, D2, Ce1, and Ce2) found in *Setaria*, *Panicum*, *Digitaria,* and *Cenchrus* isolates.

For Southern analysis, genomic DNAs from the core isolates were digested with *Acl*I, *Bam*HI, *Eco*RI, *Hin*dIII, and *Xho*I, and probed with APita766, a 766bp fragment (from O-5J) that spanned the middle of exon 1 through the end of the *AVR-Pita* ORF (see Materials and Methods, and Table S2). Under hybridization conditions used, APita766 hybridized to both *AVR-Pita1* and *AVR-Pita2*, referred to collectively as *AVR-Pita*, but not to *AVR-Pita3* [39]. Taking results with the five restriction enzymes together, copy numbers in individual isolates and RFLP types of these copies were determined. Blots of the *Acl*I digests are shown in Figure 1 as examples. This restriction enzyme was the most useful for classifying and defining RFLP types because *AVR-Pita* had an *Acl*I site just downstream from its stop codon. *Oryza* isolates of *P. oryzae* carried two copies of *AVR-Pita* on average (Figure 1A). Representative RFLP types were designated as J1, J2, J3, CH, and PO. J1 was the most common type, and was often accompanied by J2 or J3 in Japanese isolates and CH in Chinese isolates. PO was common among Indonesian isolates. A few isolates (O-28V, O-21IN) had no copies of *AVR-Pita*, indicating that *AVR-Pita* is dispensable for the survival of at least some *Oryza* isolates in natural fields.

*AVR-Pita* or its homologs were also detected in other host-specific subgroups of *P. oryzae* (*Setaria* and *Panicum* isolates) and other cryptic species of the *P*. *grisea*/*oryzae* species complex (*Digitaria* and *Cenchrus* isolates) (Figure 1B). Hybridizing fragments in *P. oryzae* isolates from *S. italica*, *S. viridis*, and *P. miliaceum* were designated as Si, Sv, and Pm, respectively. The same symbol, Si, was assigned to the homologs in Si-5I and Si-6I despite their different-sized *Acl*I fragments, because the same-sized fragments were found in *Eco*RI, *Hin*dIII, and *Xho*I digests. Homologous fragments in *P. grisea* (from *Digitaria* spp.) and *Pyricularia* sp. (CE) (from *Cenchrus echinatus*) were designated as D1/D2 and Ce1/Ce2, respectively.

When plotted on the phylogenic tree of *Pyricularia* spp. constructed by Hirata et al. [12], the *AVR-Pita* homologs were confined in the *P. grisea/oryzae* species complex, also known as the *M. grisea* species complex (Figure S1). This result suggests that *AVR-Pita* arose during the early stage of evolution of this species complex. It should be noted that *P. oryzae* isolates adapted for infection of finger millet (*Eleusine* spp.), ryegrass (*Lolium* spp.), and wheat (*Triticum* spp.) lack *AVR-Pita* homologs (Figure S1).

### *AVR-Pita* homologs map to three locations on chromosome 4 in a cross between wild *Oryza* isolates

**Figure 2:**
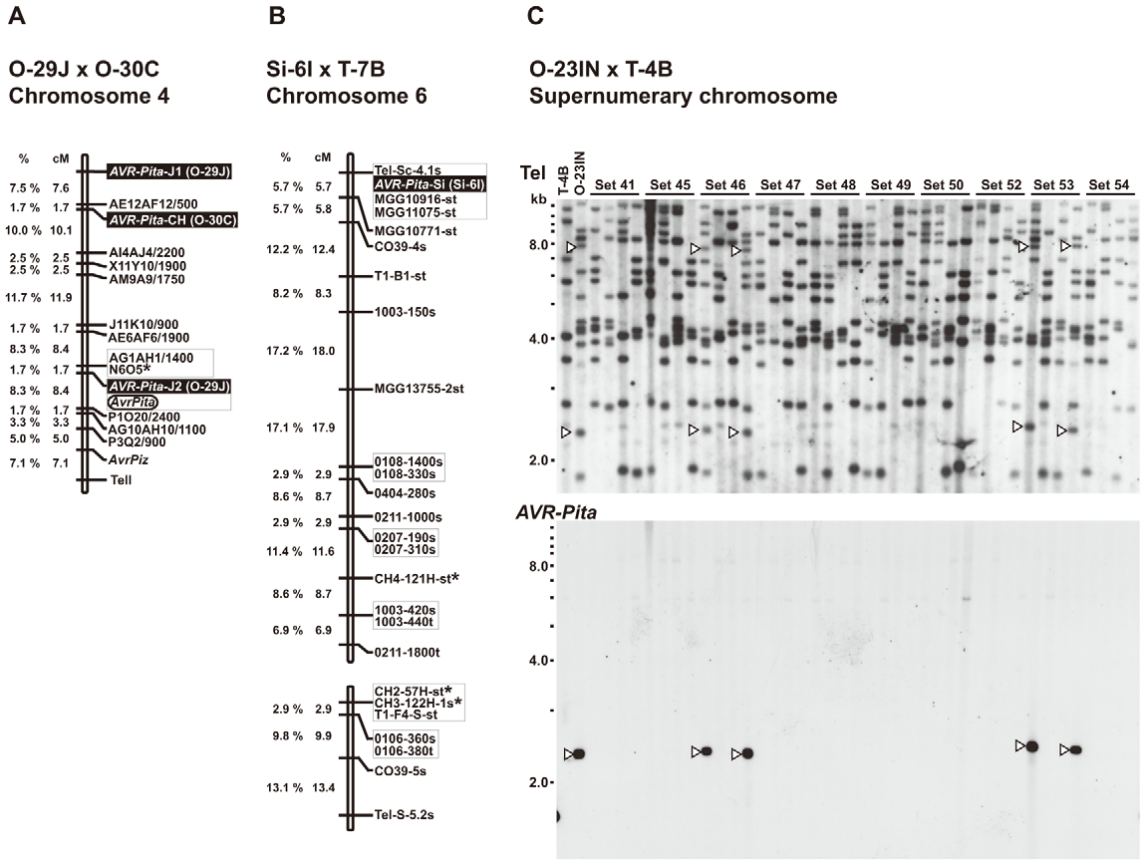
Linkage analyses of *AVR-Pita* homologs. (A) A map of chromosome 4 constructed by using an F_1_ population derived from a cross between O-29J and O-30C. Segregation data of three *AVR-Pita* homologs (*AVR-Pita-*J1(O-29J), *AVR-Pita-*J2(O-29J), and *AVR-Pita-*CH(O-30C), enclosed in black rectangles) and the avirulence on cv. Yashiro-mochi (*Pita* carrier) determined by infection assay (*AvrPita*, enclosed in an open oval) were combined with those of genetic markers reported by Luo et al. [46]. A chromosome 4 – specific marker is indicated by an asterisk. “Tell” is a telomere signal produced by Southern hybridization with a telomere repeat oligonucleotide (TTAGGG)_10_. (B) A map of chromosome 6 constructed by using an F_1_ population derived from a cross between Si-6I and T-7B. An *AVR-Pita* homolog found in Si-6I is enclosed in a black rectangle. Chromosome 6 – specific markers [47] are indicated by asterisks. Markers prefixed with “Tel” are telomere signals produced by Southern hybridization with the telomere repeat probe. Letters at the end of the markers (s and t) represent parents they are derived from. For example, CH4-121H-st represents two cosegregating fragments, one from Si-6I (*Setaria* isolate) and one from T-7B (*Triticum* isolate). (C) Southern blot analysis of an F_1_ population derived from a cross between O-23IN and T-4B. Genomic DNAs representing each meiotic product from ten tetrads (Set41 through Set54) were digested with *Bam*HI and hybridized with telomere repeat (upper panel) and *AVR-Pita* (lower panel) probes. Open arrowheads indicate restriction fragments inherited in a non-Mendelian manner.

For simplification, hereafter *AVR-Pita* homologs will be represented by their RFLP types with codes for isolates they are derived from in parentheses. For example, J2(O-29J) represents the J2 type homolog from O-29J. As mentioned above, *Oryza* isolates carry two copies of *AVR-Pita* on average, and representative combinations are J1+J3, J1+J2, and J1+CH (Figure 1A). To map the *AVR-Pita* homologs and determine which confer avirulence, a linkage analysis was performed using an F_1_ population derived from a cross between O-29J and O-30C [45]. O-29J is avirulent on rice cultivar Yashiro-mochi (harboring *Pita*) and carries J1 and J2, while O-30C is virulent on Yashiro-mochi and carries J1 and CH (Figure 1A). For mapping J1, primers specific to J1(O-29J) (AVRPita-O23-3 and AVRPita-O29-5, Table S3) were designed using a single nucleotide polymorphism between J1(O-29J) and J1(O-30C). When segregation data for J1, J2, and CH were combined with those of molecular markers reported by Luo et al. [46], the three types mapped to different locations on a single linkage group (Figure 2A). Using data from Luo et al. [46] and information from the *M. oryzae* (70–15) genome database, this linkage group was deduced to be chromosome 4. Avirulence toward rice with the *Pita* resistance gene cosegregated perfectly with J2, but not with CH or J1 (Figure 2A). Therefore, J2 appeared to be a functional avirulence gene (confirmed by transformation assay as described later). Location of the functional *AVR-Pita* homolog J2(O-29J), ∼25.5 cM apart from a telomere of chromosome 4 (Figure 2A), was intriguing, because the functional *AVR-Pita* gene first cloned by Orbach et al. [28] adjoined a telomere of chromosome 3. This led us to a hypothesis that *AVR-Pita* might have frequently been translocated to different chromosomal locations.

### *AVR-Pita* homologs reside near telomeres on chromosome 6 (*Setaria* isolate) and a supernumerary chromosome (*Oryza* isolate)

A *Setaria* pathogen from India, Si-6I, contained an *AVR-Pita* homolog designated as the Si type (Figure 1B). To reveal its chromosomal location, Si-6I was crossed with T-7B (*Triticum* isolate lacking *AVR-Pita*), and an F_1_ population was produced which consisted of 33 cultures isolated randomly. A linkage map was constructed using various AFLP, RFLP, and telomere markers (Table S4). Si(Si-6I) cosegregated completely with a telomere marker that was located on a linkage group carrying chromosome 6 – specific markers reported by Nitta et al. [47] (Figure 2B). This result suggests that Si(Si-6I) resides in a subtelomeric region of chromosome 6.

An *Oryza* isolate from Indonesia, O-23IN, contained an *AVR-Pita* homolog of the PO type (Figure 1A). Segregation analysis was performed using 10 complete tetrads from a cross between O-23IN and T-4B (*Triticum* isolate lacking *AVR-Pita*) [22]. When *Bam*HI-digested genomic DNA was hybridized with the telomere probe, most signals segregated in a 1∶1 ratio as expected (Figure 2C). However, two telomere fragments (7.8 kb and 2.4 kb) in O-23IN were inherited in a non-Mendelian manner (Figure 2C), as is characteristic of supernumerary chromosomes [48], [49]. Interestingly, the 2.4 kb telomere-containing fragment hybridized to APita766 (Figure 2C). Therefore, it appeared that PO(O-23IN) resided within 2.4 kb from a telomere of a supernumerary chromosome.

### Chromosomes containing *AVR-Pita* homologs show extreme size variation in *Oryzae* isolates, but not in *Digitaria* isolates

**Figure 3:**
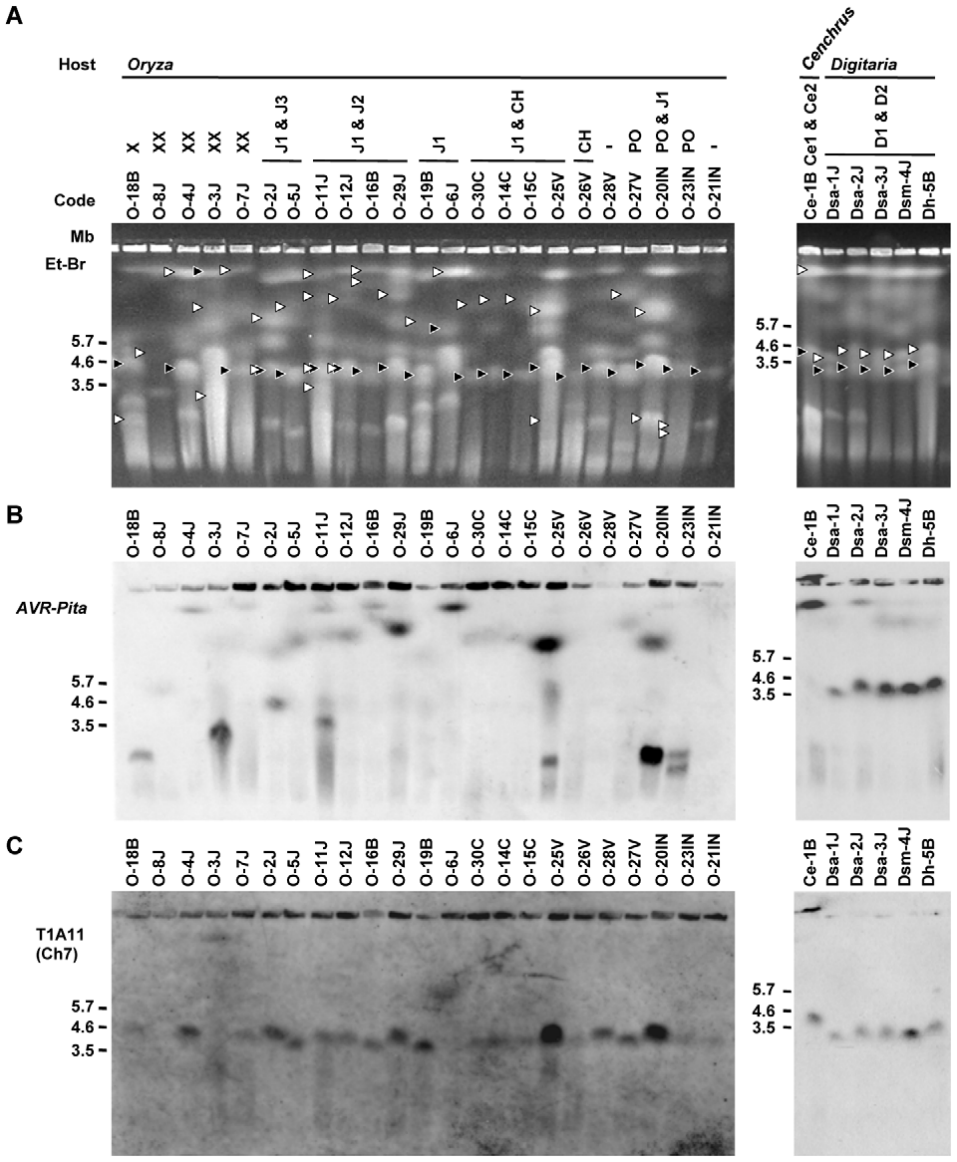
CHEF-Southern analyses of chromosomal locations of *AVR-Pita* homologs in *Pyricularia* isolates. (A) Representative examples of chromosomal DNAs separated by contour-clamped homogeneous electric field (CHEF) gel electrophoresis. White arrowheads indicate chromosomal bands that hybridized to the *AVR-Pita* probe (APita766) in (B). Black arrowheads indicate chromosomal bands that hybridized to a chromosome 7 – specific cosmid marker, T1A11 (see Figure 4A), in (C). (B) Chromosomal bands carrying *AVR-Pita* homologs. The chromosomal DNAs in (A) were blotted and hybridized with the *AVR-Pita* probe (APita766). (C) The location of chromosome 7. The membrane in (B) was reprobed with the chromosome 7 – specific cosmid marker, T1A11.

To further survey variation in *AVR-Pita*-containing chromosomes in *Pyricularia* isolates, 66 isolates were chosen from the *P. grisea/oryzae* species complex, and their chromosomal DNAs were separated by contour-clamped homogeneous electric field (CHEF) gel electrophoresis (Figure 3). Electrophoretic karyotypes varied in *Oryza* isolates (Figure 3A, left) as reported previously [49], [50]. When the gel was blotted and probed with APita766, various sized chromosomes were detected (Figure 3B, left), suggesting that *AVR-Pita* homologs were located on different chromosomes in different field isolates. In contrast, hybridization with a chromosome 7 – specific marker (T1A11, Kobe University) showed that this smallest essential chromosome was approximately 3.5 Mb throughout the *Oryza* isolates (Figure 3C, left). It should be noted that the *AVR-Pita*-containing chromosomes in several isolates (including O-23IN mentioned above) are smaller than chromosome 7 (Figure 3A).

In contrast to *Oryza* isolates, *Digitaria* isolates (*P. grisea*) showed relatively little karyotype variation (Figure 3A, right). Sizes of *AVR-Pita*-containing chromosomes were almost uniform (Figure 3B, right), even between isolates from Japan and Brazil. These chromosomes (∼4 Mb) were a little larger than chromosome 7 (Figure 3A, B, C, right). Dsa-2J showed a weak signal on the top band, but this signal was not reproducibly detected under high-stringency conditions. From these results we concluded that the D1 and D2 homologs (Figure 1B) were both located on the ∼4 Mb chromosome in all the *Digitaria* isolates. A *Cenchrus* isolate (another cryptic species) had its *AVR-Pita* homologs on the largest chromosomal band (Figure 3A, B, right).

### Chromosome-length polymorphisms, chromosome rearrangements, and *AVR-Pita* location in diverse field isolates

**Figure 4:**
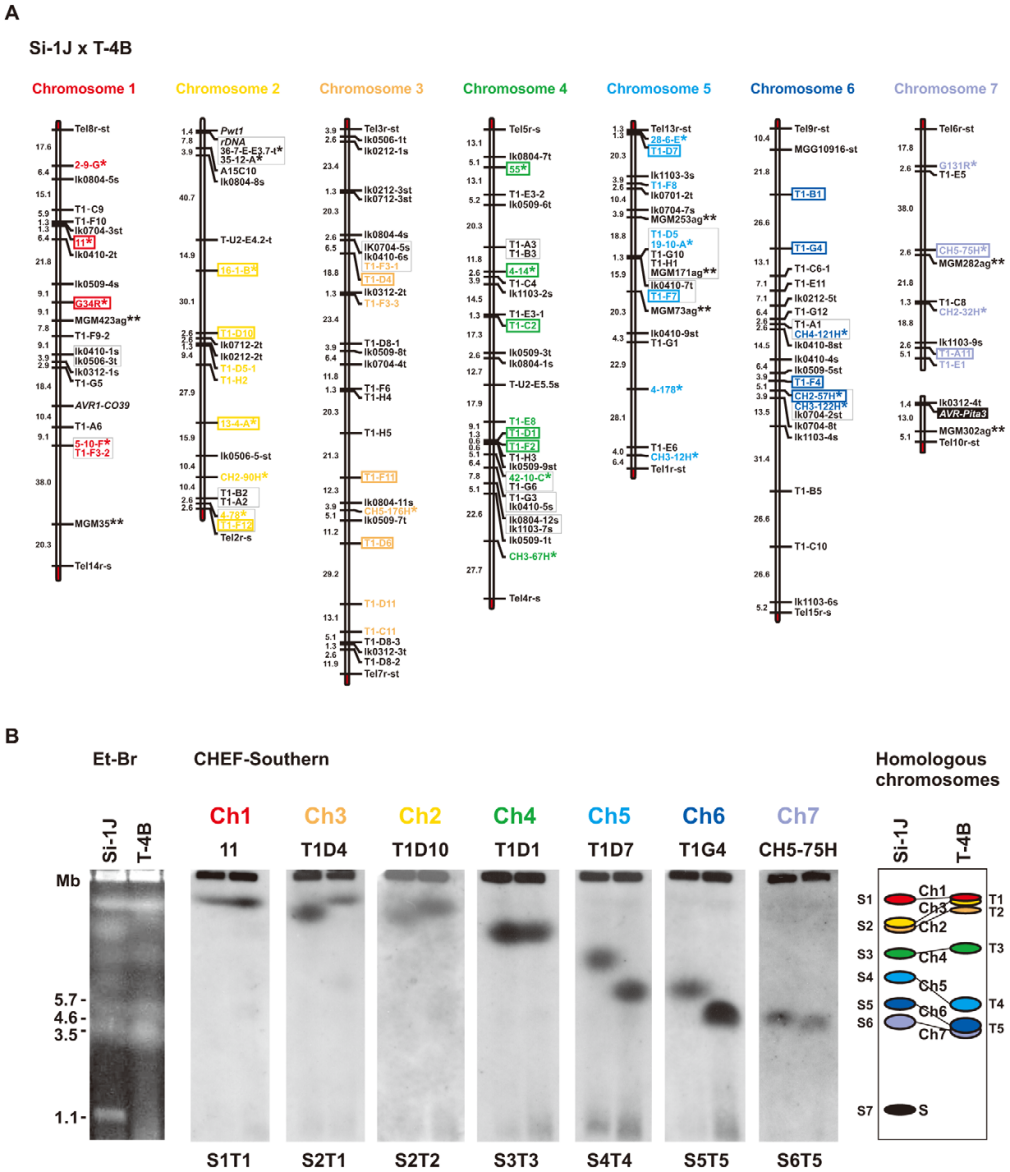
Identification of chromosome specific markers spanning the *P. oryzae* chromosomes. (A) A genetic map of a cross between *P*. *oryzae* isolates, Si-1J and T-4B. Asterisks indicate chromosome-specific markers reported by Nitta et al. [47]. Double asterisks indicate chromosome-specific SSR markers reported by Zheng et al. [52]. Markers prefixed with “Tel” are telomere signals produced by Southern hybridization with telomere repeats (TTAGGG)_10_. Letters at the end of the markers (s and t) represent parents they are derived from (s, from Si-1J; t, from T-4B). Markers in red, yellow, brown, green, light blue, blue, and violet, were used in the CHEF-Southern analysis (B) for identification of chromosomes 1, 2, 3, 4, 5, 6, and 7, respectively. Markers enclosed in rectangles were used for karyotype analysis in Figure 5A. (B) Identification of homologous chromosomes in the parental isolates (Si-1J and T-4B) on a CHEF gel. Chromosomal DNAs in the parental isolates were separated on a CHEF gel and stained with ethidium bromide (the left panel). Sizes of *Schizosaccharomyces pombe* chromosomes are indicated on the left of the gel photograph. The gel was then blotted and probed with the RFLP markers shown in the chromosome-specific colors in (A). Seven hybridization patterns (S1T1 through S6T5) are shown in the middle seven panels with marker names on the top as examples. Based on these Southern analyses, chromosome number(s) were assigned to each band (the right panel). The diagrammatic chromosomal bands are painted in the chromosome-specific colors used in (A). S (painted in black) indicates a supernumerary chromosome described previously [48].

Determining which chromosomes contain *AVR-Pita* homologs in diverse field isolates, many of which are sexually infertile and contain chromosomal rearrangements [49], [50], required hybridization probes spanning each chromosome for use in CHEF analysis. To construct a system for accurate identification of chromosomes, we performed a comparative linkage and CHEF analysis. As a mapping population, we chose an F_1_ population between Si-1J (*Setaria* isolate collected in Japan) and T-4B (*Triticum* isolate collected in Brazil) that consisted of 78 random progenies [51], because this cross produced the most abundant polymorphic markers among crosses performed in our laboratories (at Kobe University and Saga University). The final map (Figure 4A) contained 160 markers, including 60 RFLP markers developed at Kobe University (KU markers), 24 chromosome-specific RFLP markers developed at University of Wisconsin-Madison (WU markers) [47], and 7 chromosome-specific SSR markers developed by Zheng et al. [52]. Based on this map, 48 RFLP markers were selected for comparative CHEF analysis (shown in colors, Figure 4A).

When chromosomal DNAs of the parental isolates (Si-1J and T-4B) were run on a CHEF gel, chromosome-length polymorphisms were observed (Figure 4B, left panel). Chromosomal bands of Si-1J and T-4B were tentatively designated as S1 through S7 and T1 through T5, respectively, in the order of their sizes (Figure 4B, the right panel). When the gel was blotted and probed with the 48 markers, hybridization patterns were classified into seven types (S1T1 through S6T5) (Figure 4B, the middle seven panels). There was a perfect correspondence between the linkage groups (Figure 4A) and the hybridization patterns (Figure 4B). Chromosome number(s) were assigned to each band (right panel). Despite chromosome-length polymorphisms, the genomes of the Japanese *Setaria* and Brazilian *Triticum* isolates used for linkage analysis did not differ by major chromosome rearrangements such as translocations or duplications.

**Figure 5:**
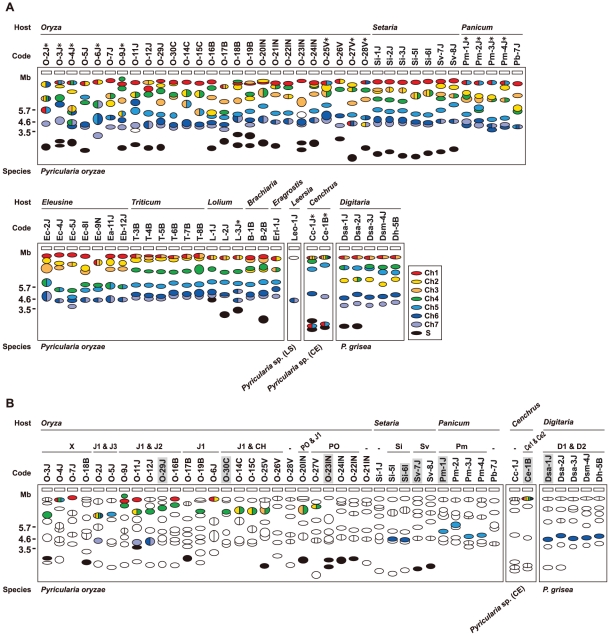
Frequent *AVR-Pita* translocation occurred independently from major chromosomal translocations or duplications. (A) A diagram of electrophoretic karyotypes of *Pyricularia* isolates revealed by Southern analyses. Blots of CHEF gels were hybridized with the markers enclosed in colored rectangles in Figure 4A. Chromosomal bands that hybridized exclusively to markers assigned to a single, same chromosome are painted in the color assigned to the chromosome in Figure 4. Chromosomal bands that hybridized to markers assigned to two or more, different chromosomes are divided with vertical lines and painted in the colors assigned to those chromosomes. Chromosomal bands that were smaller than the average size of chromosome 7 and did not hybridize to any chromosome-specific probes were considered to be supernumerary chromosomes and are painted in black. Asterisks indicate isolates which are deduced to have suffered from chromosomal rearrangements such as translocations or duplications. (B) A diagram of chromosomal locations of *AVR-Pita* homologs. Chromosomal bands that hybridized to the *AVR-Pita* probe (APita766) were painted with the chromosome-specific colors used in (A). The RFLP types defined in Figure 1 are shown above isolate codes. Hyphens indicate isolates carrying no *AVR-Pita* homologs that are detectable in the genomic Southern analysis. Shaded isolates are representatives chosen for further analyses of *AVR-Pita* flanks (see Figure 6). Isolates from *Eleusine*, *Triticum*, *Lolium*, *Brachiaria*, *Eragrostis*, and *Leersia* are omitted from this diagram because all isolates from these hosts are non-carriers of *AVR-Pita* homologs (see Figure S1).

The membranes carrying chromosomal DNAs of the 66 isolates from the *P. grisea/oryzae* species complex were sequentially hybridized with 22 markers selected from the chromosome-specific markers (Figure 4A). The results are summarized in Figure 5A. Sometimes, one chromosomal band hybridized to markers that were assigned to different chromosomes. This was assumed to be caused by a translocation, a duplication, or co-migration of more than one chromosomal DNA. These three possibilities were differentiated as follows. In some isolates, markers assigned to a single chromosome in Figure 4 were split into two subgroups which hybridized to different chromosomal bands (e.g. O-4J, chromosome 6 in blue). This was considered to be caused by a chromosomal translocation. In some isolates each of markers assigned to a single chromosome hybridized to two chromosomal bands simultaneously (e.g. O-9J, chromosome 4 in green). This was considered to be caused by a chromosomal duplication. When there was no sign of translocation or duplication, the hybridization of a single band to multiple markers was considered to be attributable to the co-migration of more than one chromosomal DNA.

The *AVR-Pita*-containing chromosomes identified by CHEF-Southern analyses with the APita766 probe are summarized in Figure 5B. This analysis confirmed results from the linkage analysis (Figure 2A, B), which located *AVR-Pita* homologs on chromosomes 4 and 6 in O-29J/O-30C and Si-6I, respectively. *AVR-Pita* homologs in *Digitaria* isolates were stably located on chromosome 6, even though the isolates tested were from Japan and Brazil. Those in *Panicum* isolates were stably located on chromosome 5. By contrast, those in *Oryza* isolates were located on various chromosomes: for example, chromosome 1 in O-7J, chromosome 4 in O-19B, chromosome 5 in O-5J, chromosome 7 in O-2J, and a supernumerary chromosome in O-18B. Although this analysis detected chromosome rearrangements in some isolates (marked by asterisks in Figure 5A), there is no correlation between these chromosome rearrangements and *AVR-Pita* location.

Supernumerary chromosomes carrying *AVR-Pita* homologs were detected in significant numbers of *P. oryzae* isolates (Figure 5B). RFLP types of the homologs on supernumerary chromosomes varied, e.g. J1 in O-17B, PO in O-22IN, and Sv in Sv-7J, suggesting that multiple events produced these supernumerary chromosomes. Interestingly, O-23IN, which was suggested to have its *AVR-Pita* homolog on a supernumerary chromosome in the segregation analysis (Figure 2C), had two supernumerary chromosomes hybridizing to *AVR-Pita* (Figure 3B, 5B). The F_1_ cultures [22] used in the segregation analysis were produced in 1998. To check chromosomal constitutions at that time, O-23IN and O-20IN were retrieved from stock cultures produced in 1996, and subjected to CHEF-Southern analysis with the *AVR-Pita* probe. O-20IN from the old stock produced the same pattern of *AVR-Pita* signals as that shown in Figure 3B, whereas O-23IN from the old stock produced only one *AVR-Pita* signal (Figure S2). This analysis revealed that the *AVR-Pita* chromosome in the old O-23IN culture corresponded to the smaller *AVR-Pita* chromosome in the current culture (Figure 3B and Figure S2). These results suggest that the larger supernumerary chromosome in the current O-23IN culture was recently generated during subculture, possibly through chromosomal rearrangements such as duplication of the smaller one.

### Association of the *AVR-Pita* homologs with the retrotransposons Inago1 and Inago2

To reveal mechanisms for the frequent changes of chromosomal locations, genomic DNA sequences around representative *AVR-Pita* homologs were examined using fosmid and plasmid clones containing the homologs (see Materials and Methods). An analysis of the J3 type was omitted because restriction analyses showed that J3 had an almost identical structure as J2. Most of the *AVR-Pita* homologs had a full length coding sequence (Figure S3). One exception was CH, which had a deletion of the 5′ half of the coding sequence (Figure S3). This homolog was apparently nonfunctional, and therefore, omitted from further analyses. Comparison of DNA sequences flanking the *AVR-Pita* coding sequence revealed that 30 bp after the stop codon and 441 bp before the start codon (yellow and orange regions in Figure S3) were conserved among *P. oryzae* isolates tested.

**Figure 6:**
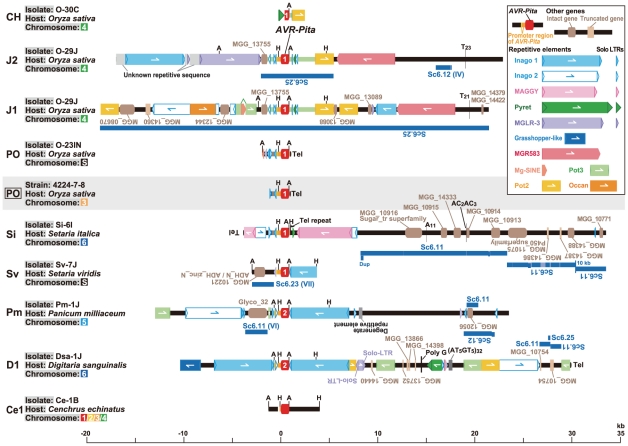
Structures around *AVR-Pita* homologs in representative RFLP types. Structures of plasmid clones (in PO, Sv, and Ce1), whole fosmid clones (in J2, J1, Si, Pm, and D1), and a partial sequence of a fosmid clone (in CH) are shown with the structure of the authentic *AVR-Pita* reported by Orbach et al. [28] (PO enclosed in a rectangle). *AVR-Pita* homologs are depicted in red at the center. Those identified as *AVR-Pita1* and *AVR-Pita2* in sequence analysis (Figure 7A) were labeled with “1” and “2”, respectively. Rectangles with triangles at both ends in the same direction indicate LTR-retrotransposons. The triangles are direct, long terminal repeats (LTRs). Detached triangles indicate solo-LTRs. Rectangles with a triangle at one end indicate LINE- or SINE-like elements. Rectangles with oppositely directed small triangles at their ends indicate DNA transposons. Directions of those elements are indicated by arrows. Brown and light brown rectangles indicate intact and truncated genes, respectively, other than transposable elements. Directions of those genes are indicated by the orientation of gene names. Black ovals designated as ‘Tel’ represent telomere repeats. Dark blue lines indicate corresponding supercontigs in the *M. oryzae* (70–15) genome database ver.6 (http://www.broadinstitute.org/annotation/fungi/magnaporthe/). The scale is shown at the bottom of the figure. A, *Acl*I sites; H, *Hin*dIII sites.

The *AVR-Pita* gene isolated from the strain 4224-7-8 closely adjoined a telomere [28]. A structure almost identical to this “authentic” *AVR-Pita* was found in the PO type carried by O-23IN, an isolate from Indonesia (Figure 6). The authentic *AVR-Pita* was, therefore, classified into the PO type and designated as PO(4224-7-8). The J1 and J2 types were accompanied by various transposable elements such as Inago1 (GenBank/EMBL accession no. AB334124), Inago2 (AB334125), Pyret (AB062507) [53], MGLR-3 (AF314096) [54], MGR583 (AF018033) [55], [56], Pot2 (Z33638) [57], Pot3 (U60989) [55], [58], and Occan (AB074754) [59]. The common feature shared by the three types (J1, J2, and PO) in *Oryza* isolates was that these *AVR-Pita* homologs were flanked by solo-LTRs of retrotransposons, Inago1 and Inago2 (Figure S3).

We hypothesized that the solo-LTRs flanking *AVR-Pita* homologs in *Oryza* isolates are remnants of full-sized copies of Inago1 and Inago2 that had once flanked the ancestral gene. Analysis of homologs from non-rice isolates provided evidence to support this hypothesis. In Pm-1J belonging to the *Panicum* host-specific subgroup of *P. oryzae*, a full-sized copy of Inago1 was detected in the right flank of *AVR-Pita* (Figure 6). This Inago1 copy had a complete structure as a retrotransposon, that is, ORFs flanked by 5′-LTR and 3′-LTR, although its nucleotide sequence contained mutations leading to a truncation of the encoded protein. A similar structure was also detected in Sv-7J belonging to the *Setaria* host-specific subgroup. Furthermore, in Dsa-1J belonging to a different cryptic species (*P. grisea*), full-sized copies of Inago1 were detected in both flanks of the *AVR-Pita* homolog (the D1 type) (Figure 6). This fosmid clone from the Dsa-1J genomic library contained telomere repeats at one end, which was 28.8 kb downstream from the *AVR-Pita* homolog (Figure 6).

Considering the *Cenchrus* isolates, Ce-1B did not have Inago1 or its solo-LTRs in the flanks of the *AVR-Pita* homolog (the Ce1 type) (Figure 6). Interestingly, when genomic DNA of Ce-1B was hybridized with an Inago1 probe, >40 signals were detected (data not shown), indicating that its genome harbors many copies of Inago1. One of the features of retrotransposons is that they are not completely excised from genomic positions in which they reside. Therefore, we suggest that the flanks of Ce1(Ce-1B) are virgin regions which have not suffered from insertions of Inago1.

In contrast to the Sv type in the *Setaria* subgroup, the Si type showed a distinct structure lacking a full-sized copy of Inago1 sequences. Instead, Si(Si-6I) was accompanied by a full-sized copy of retrotransposon MAGGY (L35053) [60] in the right flank and its truncated copy in the left flank (Figure 6). In the linkage analysis (Figure 2B), Si(Si-6I) was suggested to reside in a subtelomeric region. Actually, this *AVR-Pita* homolog was located on a Si-6J fosmid clone containing telomere repeats (Figure 6). However, the telomere was located 3.8 kb upstream from the *AVR-Pita* homolog, not downstream as in the authentic *AVR-Pita* or the PO type.

The *M. oryzae* (70–15) genome database (http://www.broadinstitute.org/annotation/fungi/magnaporthe/) contains an *AVR-Pita* homolog with >99% homology to J1(O-29J). The structure around this homolog in the database was almost identical to that of the fosmid clone containing J1(O-29J); perfect synteny was observed from one end to the other of its insert (38.9 kb) (Figure 6). On the other hand, synteny with the fosmid clone containing J2(O-29) was confined to a 7.5 kb region around the *AVR-Pita* homolog (Figure 6). The *AVR-Pita* homolog in the database was, therefore, concluded to belong to the J1 type, and designated as J1(70–15).

### Processes of rearrangements in the *AVR-Pita*-flanking regions

**Figure 7:**
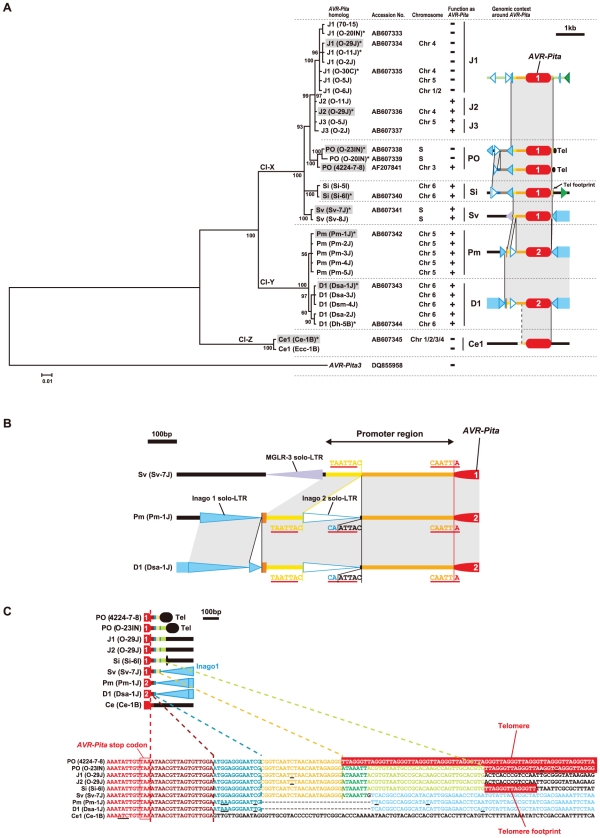
Molecular evidence suggesting the course of evolution of the *AVR-Pita* family. (A) A Bayesian tree constructed from exon sequences of *AVR-Pita* homologs. The number on each branch indicates posterior probability. The tree was rooted using *AVR-Pita3* [39] as an outgroup taxon. Shaded are representative homologs whose flanks were analyzed in detail (see Figure 6). Asterisks indicate homologs used for the transformation assay (see Figure 8). The function of the homologs as an avirulence gene, which were deduced from the transformation assay and sequence analyses, is represented by + (functional) and – (nonfunctional) in the “function as *AVR-Pita*” column. Chromosomes carrying the homologs are shown in the “chromosome” column. “S” represents a supernumerary chromosome. The RFLP types of the homologs and structures of their flanks are depicted in the right column. See Figure 6 for legends of symbols. Cl-X, Cl-Y, and Cl-Z indicate three major clusters found in the present study. (B) Structure at the 5′ flanks of *AVR-Pita* homologs suggesting a horizontal transfer. (C) Structures at the 3′ flanks of *AVR-Pita* homologs suggesting stepwise stacking of blocks of DNA fragments. Each block is painted in a distinct color. Corresponding blocks in the diagrams (upper panel) and the nucleotide sequences (lower panel) are shown in the same color. Red boxes indicate telomere repeats. Underlines indicate nucleotide substitutions.

To reveal evolutionary relationships among the structures shown in Figure 6, 29 representative homologs were amplified from 24 isolates and sequenced. Their exon sequences were aligned with those of J1(70-15) and PO(4224-7-8) (Dataset S1) and used to construct a Bayesian tree. The resulting tree of a total of 31 homologs was rooted using *AVR-Pita3* as an outgroup taxon. The RFLP types of these homologs were plotted on the tree with the diagrams showing structures in their flanks (Figure 7A). The *AVR-Pita* homologs were grouped into three clusters; Cl-X consisting of the RFLP types found only in *P. oryzae*, Cl-Y consisting of the *P. grisea* D1 and *P. oryzae* Pm types, and Cl-Z consisting of the Ce1 type. The homologs grouped into Cl-X and Cl-Y showed >98% homology to *AVR-Pita1* and *AVR-Pita2*, respectively. This result indicates that J1, J2, J3, PO, Si, and Sv found in *Oryza* and *Setaria* isolates are *AVR-Pita1* while Pm in *Panicum* isolates and D1 in *Digitaria* isolates are *AVR-Pita2*. The topology of the tree suggests that a key event in the evolution of *AVR-Pita* flanks was the insertion of full length copies of Inago1.

It should be noted that *Panicum* isolates of *P. oryzae* (Figure S1) contained *AVR-Pita2* characteristic of *P. grisea* (*Digitaria* isolates), instead of *AVR-Pita1* characteristic of the other host species-specific subgroups in *P. oryzae* (Figure 7A). The Pm and D1 *AVR-Pita2* genes shared a 99.7% identical coding sequence and a 97.4% identical promoter sequence including an inserted solo-LTR of Inago2 (Figure 7B) while the rDNA-ITS1, ITS2, actin genes, ß-tubulin genes, and calmodulin genes of the *Panicum* and *Digitaria* isolates shared 92.1%, 90.9%, 93.3%, 93.5%, and 84.7% identities in nucleotide sequences, respectively [12]. The *AVR-Pita2* genes are located in non-homologous sequences on chromosomes 5 and 6 for the Pm and D1 types, respectively (Figure 6). These results suggest that the Pm type originated from a homolog that had been horizontally transferred from *P. grisea* to *P. oryzae*.

Additional clues suggesting evolutionary processes were found in structures in the 3′ flanks of the homologs. A comparison of nucleotide sequences of the 3′ flanks revealed that this region had been constructed through stepwise stacking of blocks (Figure 7C). The eight types other than Ce1 shared a 13 bp block shown in blue. Among the eight, the six types other than D1 and Pm shared a 22 bp block shown in yellow. Among the six, the five types other than PO(4224-7-8) shared a 7 bp block shown in green. Among the five, the four types other than Sv shared a 32 bp block shown in pale green. Intriguingly, short telomere repeats were found in the right flank of the pale green block in the Si type. The real telomere on this chromosome is located at 3.8 kb upstream from the *AVR-Pita* homolog as mentioned above (Figure 6, Figure S3). These results suggest that an ancestor of the Si type had a telomere at the position shown in Figure 7C (in the 3′ flank), but that the telomeric fragment containing the homolog was then excised and fused to the same or another telomere in the opposite direction.

### Functional analysis of *AVR-Pita* homologs across the *P. grisea/oryzae* species complex

**Figure 8:**
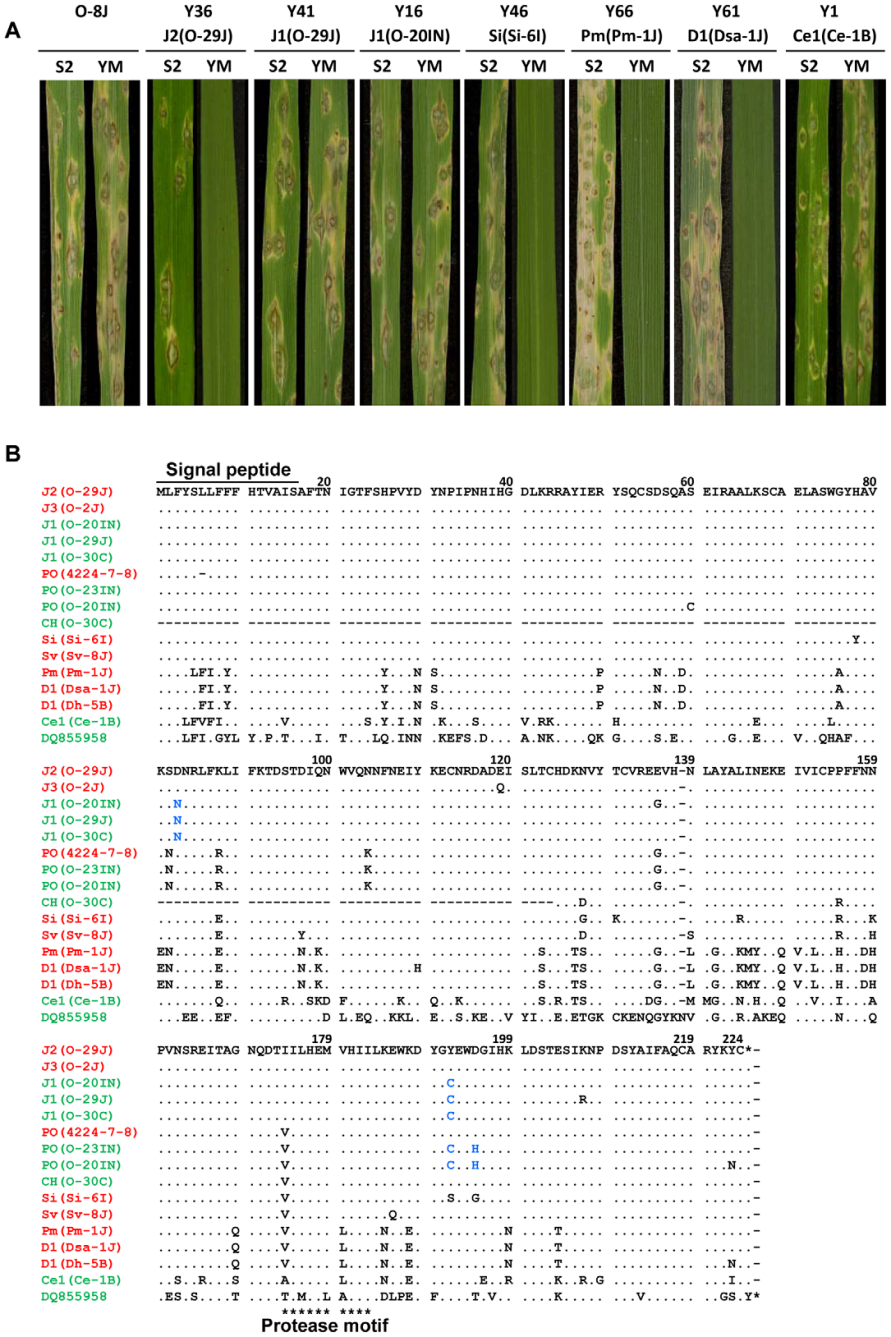
Functional analysis of *AVR-Pita* homologs with respect to avirulence activity. (A) Pathogenicity of *Oryza* isolate P-2b (O-8J) and its transformants expressing various *AVR-Pita* homologs (Y36 through Y1) on rice cultivars, Shin2 (S2) carrying *pita* and Yashiromochi (YM) carrying *Pita*, 7 days after inoculation. See Table 1 for details of the transgenes. (B) Alignment of amino acid sequences of the *AVR-Pita* homologs. DQ855958 is the accession number of *AVR-Pita3* [39] used as an outgroup in Figure 7A. Functional (triggers *Pita*-mediated resistance) and non-functional (fails to trigger *Pita*-mediated resistance) homologs are shown in red and green, respectively. Amino acids shown in blue letters indicate substitutions shared by the three non-functional J1 homologs in comparison with the functional J2(O-29J) and those shared by the two non-functional PO homologs in comparison with the functional PO (4224-7-8).

To assess avirulence activity of the *AVR-Pita* homologs, fosmid or plasmid clones (Table 1) carrying 13 representative homologs (indicated by asterisks in Figure 7A) were introduced into O-8J, an *Oryza* isolate virulent on rice cultivars Yashiro-mochi (*Pita*) and Shin2 (*pita*) (Figure 8A). Infection assays revealed that the fosmid clone with J2(O-29J) (shown in Figure 6) transformed O-8J to avirulence on Yashiro-mochi without a change in virulence on Shin2 (Figure 8A, Table 1). By contrast, the fosmid clone with J1(O-29) (shown in Figure 6) did not transform O-8J to avirulence on Yashiro-mochi. These results confirm the inference from the linkage analysis (Figure 2A) that the functional avirulence gene in O-29J is not J1, but J2. The J1 homologs were grouped into three variants at the level of nucleotide sequences of the gene (including the promoter and ORFs). These variants were amplified from genomic DNA of O-11J, O-20IN, and O-30C, and cloned into pBluescript II SK (+). None of these clones changed the virulence of O-8J on Yashiro-mochi (Table 1). Plasmid clones containing our PO homologs also did not have any effect on the virulence of O-8J. On the other hand, all plasmid clones containing Si, Sv, Pm, and D1 transformed O-8J to avirulence on Yashiro-mochi without a change in virulence on Shin2 (Figure 8A, Table 1). This result indicates that all these homologs encode proteins that are recognized by the rice resistance gene product Pita in spite of being derived from non-rice isolates. Only one type from the non-rice isolates, Ce1, failed to trigger *Pita*-mediated resistance.

**Table 1:** Transformants of rice isolate P-2b (O-8J) with *AVR-Pita* homologs and their pathogenicity on rice cvs. Yashiromochi (YM) and Shin2 (S2).

	Transgene				Pathogenicity^a^ on	
		Insert				
Transformant	Plasmid	Origin	Type	Size (kb)	YM	S2
Y6–10	pBluescript II SK (+)	-	-	0	0/5	0/5
Y36–40	pTA-O29-F8	O-29J	J2	39.9	3/5	0/5
Y41–45	pTA-O29-F39	O-29J	J1	38.9	0/5	0/5
Y31–35	pTA-O30-28	O-30C	J1	1.8	0/5	0/5
Y11–15	pTA-O11-1	O-11J	J1	1.8	0/5	0/5
Y16–20	pTA-O20-7	O-20IN	J1	1.8	0/5	0/5
Y21–25	pTA-O20-12	O-20IN	PO	2.1	0/5	0/5
Y26–30	pTA-O23-17	O-23IN	PO	2.1	0/5	0/5
Y46–50	pTA-S6-P4.6-1	Si-6I	Si	4.6	2/5	0/5
Y51–55	pTA-S7-3-22	Sv-7J	Sv	1.5	4/5	0/5
Y66–70	pTA-P1-B5.5-5	Pm-1J	Pm	5.5	5/5	0/5
Y61–65	pTA-D1-E3.6-1	Dsa-1J	D1	1.6	5/5	0/5
Y56–60	pTA-D5-75	Dh-5B	D1	1.6	5/5	0/5
Y1–5	pTA-X4-5-32	Ce-1B	Ce1	2.1	0/5	0/5

a No. of avirulent transformants / No. of transformants tested.

Avirulence function of the other homologs shown in Figure 7 was determined by sequence analyses or infection assay with wild isolates carrying them. For example, J1(70–15) from the database was considered to be non-functional (Figure 7A) because its nucleotide sequence (spanning the entire gene) was identical to that of J1(O-20IN), which was non-functional in the transformation assay (Figure 8A). Similarly, J3(O-5J), which shared a 100% identical nucleotide sequence with J2(O-29J), was considered to be functional as an avirulence gene. The five homologs belonging to the Pm type were all deduced to be functional (Figure 7A) because their nucleotide sequences were identical to that of Pm(Pm-1J). Taken together, all *AVR-Pita* homologs in non-rice isolates of *P. oryzae* and *P. grisea* were concluded to be functional as avirulence genes (Figure 7A).

Amino acid sequences of the *AVR-Pita* homologs were aligned and compared (Figure 8B). Within *Oryza* isolates, the homologs differed from one another by only a few amino acids. For example, J1(O-30C) differed from J2(O-29J) only by two amino acids, at the 83rd and 192nd positions. Nevertheless, the former was non-functional while the latter was functional. Similarly, PO(O-23IN) (non-functional) differed from PO(4224-7-8) (functional) only by two amino acids at the 192nd and 195th positions. When they were compared with homologs in other host-specific subgroups of *P. oryzae* or *P. grisea*, however, a high diversity of amino acid sequences was observed. For example, Si(Si-6I) and D1(Dsa-1J) differed from J2(O-29J) by 10 and 38 amino acids, respectively. Nevertheless, these three homologs were all functional. These results suggest that the *AVR-Pita* homologs are under different selection pressures within and outside the *Oryza* subgroup.

### Nonfunctional *AVR-Pita3* has been stably localized on chromosome 7

The *AVR-Pita* homologs described above did not include *AVR-Pita3*, a non-functional homolog reported by Khang et al. [39], because the APita766 probe did not hybridize to *AVR-Pita3*. To analyze *AVR-Pita3*, a fragment (958 bp) was amplified from genomic DNA of Ei-13I using *AVR-Pita3*-specific primers (Table S3). Direct sequencing of this fragment confirmed that it belonged to the *AVR-Pita3* class. Southern hybridization with this *AVR-Pita3*-specific probe (APita3-958) indicated that *AVR-Pita3* is widely distributed in *P. oryzae*, but absent in the other species (data not shown) as reported by Khang et al. [39].

Khang et al. [39] further reported that *AVR-Pita3* was located on chromosome 7. APita3-958 produced the S6T5 pattern (Figure 4B) (which is specific to chromosome 7 markers) in Southern hybridization with the chromosomal DNAs of Si-1J and T-4B, indicating that the *AVR-Pita3* homologs in these isolates are also located on chromosome 7. When *Bam*HI digests of their genomic DNA were hybridized with APita3-958, 7.9 kb and 5.0 kb signals were detected in Si-1J and T-4B, respectively. In the mapping population derived from their cross, the two *AVR-Pita3* signals (7.9 kb and 5.0 kb) cosegregated completely in repulsion and were mapped to a linkage group assigned to chromosome 7, apart from a telomere marker by 18.1 cM (Figure 4A).

**Figure 9:**
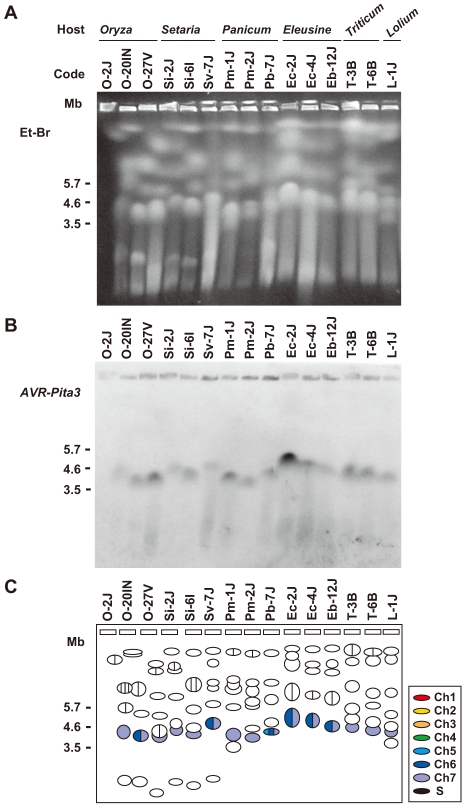
CHEF-Southern analyses of chromosomal locations of *AVR-Pita3* in representative isolates of *P. oryzae*. (A) Chromosomal DNAs separated in the contour-clamped homogeneous electric field (CHEF). The CHEF gel was stained with ethidium bromide. Note that the left five samples did not run straight. (B) Chromosomal bands carrying *AVR-Pita3*. The chromosomal DNAs in (A) were blotted and hybridized with the *AVR-Pita3* probe (APita3-958). (C) Identification of the chromosomes hybridizing to the *AVR-Pita3* probe. Chromosomes on the gel (A) were identified by reprobing the membrane (B) with chromosome – specific markers; e.g., T1-A11 and CH5-75H for chromosome 7 and T1-G4 for chromosome 6. Chromosomal bands that hybridized to the *AVR-Pita3* probe in (B) were painted with the chromosome-specific colors used in Figure 5A.

To examine whether *AVR-Pita3* has also frequently moved to different chromosomal locations, representative isolates were chosen from each host-specific subgroup of *P. oryzae*, and their chromosomal DNAs were again separated on a CHEF gel (Figure 9A). When the gel was blotted to a membrane and hybridized with APita3-958, similar sized fragments were identified for all isolates (Figure 9B). A comparison with the karyotype profiles on the other gel (Figure 5A) suggested that all of these signals corresponded to chromosome 7. To confirm it, the membrane was reprobed with chromosome 7 – specific markers (T1-A11 and CH5-75H). In all isolates, these chromosome 7 – specific markers hybridized to the chromosomal bands that hybridized to APita3-958 (Figure 9C). These results suggest that *AVR-Pita3* has been stably located on chromosome 7 during the course of parasitic specialization into the host-adapted subgroups of *P. oryzae*.

The membrane shown in Figure 9A contained chromosomes from three *Oryza* isolates. The membrane shown in Figure 3A (left) surveyed chromosomes from additional 20 *Oryza* isolates. Reprobing this membrane with APita3-958 produced the same pattern of chromosome 7-specific bands shown in Figure 3C. This suggests that *AVR-Pita3* has been stably located on chromosome 7 during the course of race differentiation in the *Oryza* isolates.

## Discussion

### Multiple translocation of *AVR-Pita* homologs during parasitic specialization of the *P. grisea/oryzae* species complex

The most striking finding in the present study is that *AVR-Pita* is highly variable in its chromosomal location. In *P. grisea*, *AVR-Pita2* is located within 30 kb of telomeric repeat sequence on chromosome 6 (Figure 6). *AVR-Pita1* homologs in *Setaria* isolates (*P. oryzae*) reside at a different location near a chromosome 6 telomere (Si homologs) or on a supernumerary chromosome (Sv homologs) (Figure 6). The *AVR-Pita* chromosomal position was most variable in *Oryza* isolates, which were derived from asexual populations evolving in response to periodic deployment of the *Pita* resistance gene in rice (Figure 5B). In diverse *Oryza* isolates, *AVR-Pita1* was absent or present in 1 or 2 copies residing on chromosomes 1, 3, 4, 5 or 7, or on different supernumerary chromosomes. Additionally, *AVR-Pita1* types J1, J2 and CH mapped to three different locations on chromosome 4. This extraordinary variability in chromosomal position contrasts with the stable location of *AVR-Pita3*, a family member that lacks avirulence activity. *AVR-Pita3* was stably localized on chromosome 7 in all isolates of all host-adapted forms of *P. oryzae* (Figure 9).

One simple explanation for the chromosomal position variation of *AVR-Pita* is that homologs have frequently been translocated to different chromosomal locations during the course of evolution of the *P. grisea/oryzae* species complex. An alternative explanation is that *AVR-Pita* originally occurred as a dispersed gene family in the ancestral strain, but family members were later lost in specific lineages (differential gene-loss). The large numbers of ancestral family members required (at least 12) for the gene-loss scenario make this hypothesis seem less likely, especially since contemporary isolates contain at most 3 copies of *AVR-Pita*. Additionally, the structures at the 3′ flanks of the *AVR-Pita* homologs indicate stepwise stacking of blocks of DNA fragments (Figure 7C), which is consistent with multiple steps of movement rather than with a process of differential gene-loss.

Aboukhaddour et al. [61] reported that genes for host-selective toxins (*ToxA* and *ToxB*) in *Pyrenophora tritici-repentis* were located on different sizes of chromosomes in different isolates. Unlike the case with *AVR-Pita*, however, most of these chromosomes were homologous. We shall call the frequent movement associated with *AVR-Pita* homologs “multiple translocation”, although the unit of movement in all cases seems to be a small DNA fragment (Figures 6 and 7C). It should be noted that the multiple translocation of *AVR-Pita* is not due to disintegration of chromosomal constitution. The mapping and CHEF analyses of the Si-1J x T-4B cross (Figure 4) suggested that, in spite of the chromosome-length polymorphisms between these two diverse parental isolates from the *Setaria* and *Triticum* host-adapted forms, the major, trunk regions of their chromosomes were syntenic. The relative chromosomal stability of the neutral chromosome-specific markers in our study resembles the stability of the nonrecognized *AVR-Pita3* gene on chromosome 7 (Figure 9B, C).

*AVR-Pita* homologs are flanked by various transposable elements, suggesting that these elements have been involved in the multiple translocation events. Khang et al. [39] found fragments of retrotransposons, RETRO7-1 and RETRO6-1, in flanking regions of *AVR-Pita* homologs. The present study showed that these retrotransposons are portions of Inago1 (AB334124) and Inago2 (AB334125), respectively, and that full-sized copies are present in the flanking regions of some homologs (Pm and D1, Figure 6). All *AVR-Pita* homologs are accompanied by full-sized copies and/or solo-LTRs of Inago1, suggesting that this element has had a role in *AVR-Pita* movement (Figure 6). Based on this fact and phylogenetic relationships (Figure 7A), we suggest that the insertion of Inago1 into flanks of ancestral *AVR-Pita* homolog(s) was a key event that produced basic structure(s) associated with the *AVR-Pita* mobility. Retrotransposons and their solo-LTRs are known to mediate ectopic recombination through homologous recombination between preexisting elements located at non-allelic positions [62]. *AVR-Pita* may have been translocated through ectopic recombination mediated by Inago1 and its solo-LTR.

### Why have *AVR-Pita* homologs undergone multiple translocation?

The extraordinary mobility of *AVR-Pita* in *Oryza* isolates compared to that in the other *P. oryzae* host-adapted forms suggests that the multiple translocation is associated with the long history of their arms race with rice varieties. In addition, the high mobility of *AVR-Pita* in contrast to *AVR-Pita3*, which lacks avirulence activity, suggests that the multiple translocation of *AVR-Pita* is associated with selection by *Pita* in rice. Then the first key question is why the recognition by *Pita* leads to the multiple translocation of *AVR-Pita*.

In laboratory studies, Orbach et al. [28] reported frequent spontaneous mutation of *AVR-Pita* to virulence on rice with *Pita*, and found that the majority of events were deletions of all or a portion of the gene. Similar mutations in *AVR-Pita*, including frequent deletions, are responsible for breakdown of *Pita* in the field [33], [36]. Takahashi et al. [35] recently reported that *Oryza* isolates collected in Japan carried two paralogs, *AVR-Pita1^JA^* and *AVR-Pita1^JB^*, which presumably correspond to J2/J3 and J1, respectively, and suggested that virulence on *Pita* has mainly evolved via deletion of *AVR-Pita1^JA^*. Nevertheless, the avirulence homolog is maintained in *Oryza* isolates from farmer's fields (Fig. 1). This leads to a second key question of how *AVR-Pita* is maintained in the current population of *Oryza* isolates. Without recovery of *AVR-Pita*, the system of continuous gene loss should not be sustainable.

We suggest that these two key questions could be simultaneously answered by assuming that the multiple translocation of *AVR-Pita* is associated with its frequent deletion and with recovery mediated by its transfer among individuals in asexual populations. It is well known that, after planting of resistant cultivars comes to a halt, the “unnecessary” virulence alleles at avirulence loci tend to disappear through “stabilizing selection” [63]. In other words, the frequency of avirulence genes in a population tends to increase again after the corresponding resistance genes are removed from the field. Recovery could result from restoration of minor races or migration of races from other regions. However, another possibility is that the avirulence genes may have actually been regained by the races that defeated the resistance genes. If DNA fragments carrying the avirulence genes are introduced into virulent isolates, they may insert into unstable chromosomal sites such as at telomeres. In this process of recovery, the avirulence genes will be recognized to have undergone multiple translocations.

Our data include evidence consistent with the transfer of genetic materials between individuals in the species complex. *AVR-Pita1* was found in *P. oryzae* isolates while *AVR-Pita2* was found in *P. grisea* isolates with one exception. The exception was the Pm type of *AVR-Pita2* found in *Panicum* isolates of *P. oryzae* (Figure 7A). The nucleotide sequences of the *AVR-Pita2* fragments in the *Digitaria* and *Panicum* isolates are more highly conserved than expected conserved genes such as the actin and ß-tubulin genes. It should also be noted that both types of *AVR-Pita2* (Pm in *P. oryzae* and D1 in *P. grisea*) had an insertion of a solo-LTR of Inago2 into their 5′ flanks (Figure 7B). Retroelements are good markers for tracing evolutionary processes, and their insertion patterns can be treated as synapomorphic characters [64]. Although we cannot completely rule out the possibility that the Pm and D1 *AVR-Pita2* genes have been highly conserved after introduction from a common ancestor, these data strongly suggest that *AVR-Pita2* has been horizontally transferred beyond species. Therefore, there is no reason to exclude the possibility that *AVR-Pita* has been transferred among individuals within the same species (*P. oryzae*) and within the same host-specific subgroup (*Oryza* isolates).

If this scenario is correct, our results suggest that rare events in which *AVR-Pita* had been inserted into a new chromosomal location were selected for in the *Oryza* population. This implies that the avirulence allele of *AVR-Pita* confers a fitness advantage that is not provided by *AVR-Pita3*. So far, attempts to understand how *AVR-Pita* functions to promote disease in the absence of the *Pita* resistance gene have not succeeded. Defining this fitness role for the different *AVR-Pita* family members remains an important priority.

### Potential role of supernumerary chromosomes in the multiple translocation of *AVR-Pita* homologs

An additional significant finding from this study is that *AVR-Pita* frequently appears on supernumerary chromosomes (Figure 5B), which were previously reported to be common in *Oryza* isolates reproducing asexually in the field [49]. Supernumerary chromosomes are extra chromosomes (B chromosomes) composed primarily of DNA not found in all representatives of the species [65]. In some fungal species, genes on such chromosomes play important roles in host-parasite interactions. In *Nectria haematococca*, a supernumerary chromosome carries genes encoding cytochrome P450 monooxygenases for phytoalexin detoxification and other genes contributing to pathogenicity [66], [67]. In *Alternaria alternata*, supernumerary chromosomes carry genes involved in the production of host-specific toxins [68]–[71].

Supernumerary chromosomes are structurally unstable [48], [66]. Here, we will point out three properties of supernumerary chromosomes that may have been associated with the dynamics of avirulence genes. First, supernumerary chromosomes are known to be spontaneously lost from the genome [70] because they are dispensable. This property of supernumerary chromosomes would confer a selective advantage upon isolates carrying avirulence genes on those chromosomes; such isolates could adapt quickly to cultivars carrying corresponding resistance genes by losing the supernumerary chromosomes. Second, supernumerary chromosomes are suggested to have contributed to gene expansion [72]. Some of the “expanded” genes appeared to have resulted from gene duplication events [72]. The present results with O-23IN suggest that supernumerary chromosomes may contribute to the gene duplication through duplication of themselves. Finally, supernumerary chromosomes or small chromosomes are suggested to have been horizontally transferred [68], [73], [74]. Supernumerary chromosomes in the *P. grisea/oryzae* species complex may have played significant roles in the loss, duplication, and multiple translocation of *AVR-Pita*.

### How and where can *AVR-Pita* be transferred among isolates?

In laboratory studies, *Oryza* isolates are known to exchange DNA through parasexual recombination following hyphal anastomosis and transient diploid formation [75]. Zeigler et al. [17] suggested that parasexual DNA exchanges occur at a detectable frequency in the field. Furthermore, *Oryza* isolates are sometimes isolated from blast lesions on nonhost weeds, e.g., green bristlegrass (*Setaria viridis*) and crabgrass (*Digitaria sanguinalis*), around paddy fields (data not shown). They are considered to have colonized blast lesions produced by adapted isolates (*Setaria* isolates and *Digitaria* isolates) through opportunistic infection. Such lesions on weeds may provide opportunities for different isolates to exchange supernumerary chromosomes or DNA fragments carrying avirulence genes. Further studies are needed to test this hypothesis.

### Does multiple translocation occur with other blast avirulence genes?

*AVR-Pii*, an avirulence gene corresponding to the *Pii* resistance gene of rice, appears to be subtelomeric like *AVR-Pita*, and is located on chromosome 7 [76]. *AVR-Pia*, an avirulence gene corresponding to the *Pia* resistance gene, also appears to be subtelomeric and is located on chromosome 7 in a cross [77]. However, in studies by Yasuda et al. [76], *AVR-Pia* was linked to three markers from chromosome 5 and one marker (40-12-G) from chromosome 7 that was presumed to reside in chromosome 5 in their isolates. *AVR-Pik*, an avirulence gene corresponding to the *Pik* resistance gene, was linked to DNA markers on chromosome 1 in some crosses [46], [78]. In Japanese isolate 84R-62B, however, this gene was located on a small 1.6Mb chromosome derived from fusion of a portion of chromosome 1 to a supernumerary chromosome [79]. These results suggest that *AVR-Pia* and *AVR-Pik* have undergone translocation, sometimes via supernumerary chromosomes. Our preliminary experiments also suggest that *AVR-Pia*, *AVR-Pik*, and *AVR-Pii*, have undergone multiple translocation. These data will be reported elsewhere.

Are the multiple translocations of these avirulence genes also mediated by loss/gain processes? Using Japanese isolates, Miki et al. [31] found a perfect correspondence between the presence or absence of *AVR-Pia* signals and avirulence or virulence on *Pia*, respectively. They also found that the homologs in avirulent isolates shared 100% identical nucleotide sequences. Similar results were obtained by Yoshida et al. [32]. These results suggest that the primary mechanism for *Oryza* isolates to overcome *Pia* is deletion of *AVR-Pia* from their genomes. *AVR-Pik* homologs (*AVR-Pik/km/kp* homologs) exhibited both presence/absence and nucleotide polymorphisms [32]. Yoshida et al. [32] found some isolates that were virulent on *Pik* in spite of carrying *AVR-Pik* homologs (A, B, C). These nonfunctional homologs were presumed to be paralogs rather than orthologous variants of the functional homolog (D) because several isolates contained both A and D homologs. This distribution of homologs resembles that of *AVR-Pita*. That is, most *Oryza* isolates carry *AVR-Pita* homologs, but they are composed of functional and non-functional paralogs (Figs. 1, 2), and deletion of the functional paralogs (J2 and J3) is primarily associated with the gain of virulence on *Pita* (Fig. 1 and unpublished data). Consequently, deletion of the functional *AVR-Pik* paralog seems to be a common mechanism for *Oryza* isolates to overcome *Pik*. *AVR-Pii* also appears to be deleted in virulent strains of the fungus [32]. Taken together, these results suggest that *AVR-Pia*, *AVR-Pik, and AVR-Pii* have undergone multiple translocations through the loss/gain processes. It is not clear why *AVR-Pita* and *AVR-Pik* have developed paralogs while *AVR-Pia* has not. It may be attributable to the structure in their flanking regions. Alternatively, the loss of *AVR-Pita* and *AVR-Pik* may pose higher fitness costs to isolates. If this is the case, the “nonfunctional” paralogs in terms of avirulence should retain their original function as effectors.

The involvement of the loss/gain processes in the arms race of *P. oryzae* against rice has also been suggested by Yoshida et al. [32] based on their observation that the majority of candidate effector loci in *P. oryzae* displayed low nucleotide diversity while frequently showing presence/absence polymorphisms. We assume that, in general, effector genes located on unstable chromosomal regions have a potential to be translocated sometimes within an individual and sometimes through loss/gain processes. *M. oryzae* may use the loss/gain system for adaptaion to resistance genes.

Jones and Dangl [80] noted that effector genes are often associated with transposable elements or telomeres and are commonly observed as presence/absence polymorphisms across bacterial and fungal pathogens. This observation led them to suggest that the simplest pathogen response to host recognition is to jettison the detected effector gene, provided the population's effector repertoire can cover the potential loss of fitness on susceptible hosts. It is of interest to examine whether the multiple translocation of avirulence genes may also be observed in other plant pathogens.

### Comparison with telomeric genes in other microbes

Whatever the mechanism, multiple translocation has resulted in the dispersal of *AVR-Pita* to various sites (often in subtelomeric regions) of various chromosomes in the population of the *P. grisea/oryzae* species complex. Similar dispersal has been recognized in subtelomeric gene families of yeast that are involved in niche-specific processes [81]–[83]. Brown et al. [83] suggested that the evolvability of such subtelomeric gene families allows rapid adaptation to novel niches.

From the viewpoint of the importance of subtelomeric gene families in adaptation, we also notice a similarity to the immune evasion systems in animal pathogens. Several *P. oryzae* avirulence genes are mapped to terminal regions of chromosomes [46], [76], [77], [84]–[86], which are extraordinarily unstable in some strains [85]. One tempting hypothesis from this observation is that a telomere-based switching mechanism might underlie the pathogenic variability as in the animal pathogens [87]. However, Rehmeyer et al. [87] failed to identify the massively amplified families of surface protein genes or genes coding for secreted proteins in subtelomeric regions, which makes it unlikely that this fungus uses switching mechanisms like those in animal pathogens to evade the host defenses [85].

Here, we suggest that the strategy for host adaptation adopted by (fungal) plant pathogens is fundamentally different from that adopted by (protozoan) animal pathogens although they initially appear similar. First, antigenic genes are indispensable for the survival of the animal pathogens whereas individual avirulence genes are dispensable for the survival of the plant pathogens (as exemplified by O-28V and O-21IN in Figure 1). This makes it possible for the plant pathogens to survive after deletion of avirulence genes from their genomes, although the avirulence gene may provide an advantage in fitness in the field that favors regaining the gene after the resistance gene is removed [3]. Second, an individual of the animal pathogens carries a huge number of antigenic genes, e.g., ∼60 *var* genes in *P. falciparum* and more than 1000 VSG (variant surface glycoprotein) genes in *T. brucei* [88], whereas an isolate of the plant pathogens carry at most a few copies of individual avirulence genes. Third, antigenic genes are highly variable whereas avirulence genes themselves are relatively stable (Figure 8B). The high posterior probabilities on branches of the Bayesian tree (Figure 7A) suggest that the *AVR-Pita* homologs have not suffered from shuffling of gene fragments. Taken together, the interaction between animals and their protozoan pathogens appears to be a battle between individuals whereas the interaction between plants and their fungal pathogens seems to be a battle between populations. An animal host produces various antibodies in an individual. To evade the recognition by the antibodies, an animal pathogen carries a huge reservoir of antigenic genes in an individual. On the other hand, a plant individual carries at most a few resistance genes against a given pathogen. As a population, however, plants carry various resistance genes and, by using this variation, they survive pathogen attacks. Similarly, an isolate of plant pathogens carries at most a few copies of avirulence genes. As a population, however, a plant pathogen carries various avirulence genes at various chromosomal sites. In other words, a plant pathogen carries a reservoir of avirulence genes in its population. A *Pyricularia* population composed of various isolates, each of which carry avirulence genes at different sites on different chromosomes, may be an equivalent to an animal pathogen individual which carries antigenic genes at various sites of various chromosomes in its own nucleus. The dynamic adaptation of fungal plant pathogens may be primarily achieved by the deletion and recovery of avirulence genes using a population as a unit of adaptation. It appears to be the dispensability of individual avirulence genes that enables the fungal plant pathogens to adopt this strategy.

## Materials and Methods

### *Pyricularia* isolates and strains

*Pyricularia* isolates used in the present study are listed in Table S1. In addition to these field isolates, four F_1_ populations derived from crosses between them were employed for molecular mapping: (i) 60 random cultures derived from a cross, O-29J (84R-62B) x O-30C (Y93-245c-2) [46], (ii) 40 cultures (composed of ascospore cultures representing each meiotic product in ten tetrads) derived from a cross, O-23IN (PO12-7301-2) x T-4B (Br48) [22], (iii) 78 random cultures derived from a cross, Si-1J (GFSI1-7-2) x T-4B (Br48) [51], and (iv) 33 random cultures derived from a cross, Si-6I (IN77-20-1-1) x T-7B (Br116.5). In (i), (iii), and (iv), each culture was derived from a distinct ascus. The (iv) population was produced in the present study using methods described previously [89]. The eight ascospores in a single tetrad are derived by mitotic division of each product of meiosis. The (ii) population included only one representative of each of the four products of meiosis, which are easily differentiated based on cultural morphologies, mating types and other markers (Figure 2C). All cultures were maintained as described previously [12].

### DNA extraction and Southern hybridization

Fungal genomic DNAs were extracted as described by Nakayashiki et al. [90], digested with restriction enzymes, electrophoresed on 0.7% agarose gels, and blotted to Hybond N+ membranes (GE healthcare, Buckinghamshire, U.K.). Southern hybridization of the genomic DNA digests was performed using the Alkphos Direct Labeling and Detection System (GE healthcare) for the telomere repeat probe (a synthetic oligonucleotide (TTAGGG)_10_) or the ECL Direct Nucleic Acid Labeling and Detection System (GE healthcare) for the other probes, according to the manufacturer's instructions. To detect *AVR-Pita*, a 766 bp fragment was amplified from O-5J using *AVR-Pita*-specific primers (AVRPita-ORF-F2 and AVRPita-ORF-R2) (Table S3), cloned into pBluescript II SK (+), and established as pP4-1 (Table S2). The insert was amplified from pP4-1 with the same primers and used as an *AVR-Pita*-specific probe, APita766. Sequence analysis revealed that the APita766 fragment was derived from J3(O-5J), the functional copy of O-5J. Homology of the APita766 fragment to *AVR-Pita1*, *AVR-Pita2*, and *AVR-Pita3* [39] was >98%, ∼91%, and ∼70%, respectively, suggesting that APita766 hybridizes to *AVR-Pita1* and *AVR-Pita2*, but not to *AVR-Pita3*, with the hybridization conditions used. To detect *AVR-Pita3*, a 958 bp fragment was amplified from Ei-13I using *AVR-Pita3*-specific primers (AVRPita-G22-2 and AVRPita-G22-3) (Table S3), and used as an *AVR-Pita3*-specific probe, APita3-958.

### Genetic mapping

Molecular markers used in the genetic mapping are listed in Table S4. Clones for the WU markers were provided by S.A. Leong, University of Wisconsin-Madison, U.S.A. Nucleotide sequences of the WU markers were provided by M. Farman, University of Kentucky, U.S.A. Cosmid clones for the KU markers were selected from genomic libraries of Si-1J and T-4B which were constructed in the pMLF2 vector as described by Nakayashiki et al. [53]. SSR markers developed by Zheng et al. [52] were used as additional chromosome-specific markers. PCR products amplified from SSR loci were fractionated by electrophoresis through 6% denaturing polyacrylamide gels and stained using SILVER SEQUENCE DNA Staining Reagents (Promega, Madison, WI, USA). AFLP analysis was performed using AFLP Analysis System for Microorganisms (Invitrogen, Carlsbad, CA, USA) as described previously [51]. Segregation data were analyzed using MAPMAKER Macintosh V2.0 with the haploid program. Parameters for map construction were a minimum LOD of 3.0 and a maximum theta of 0.4. The Kosambi mapping function was employed to compute recombination distances in centimorgans (cM).

### Electrophoretic karyotyping

Preparation of gel plugs, running conditions for CHEF gel electrophoresis, staining, blotting and hybridization procedures were described previously [48]. To prevent chromosomal degradation caused by Tris-radical reaction, 200 µM thiourea was added to TBE buffer as described by Römling and Tümmler [91].

### Plasmid, cosmid, and fosmid clones

Cosmid, fosmid, and plasmid clones used for sequence analyses or complementation assays are listed in Table S2. A cosmid library of O-11J (2403-1) was constructed in the pMLF2 vector as described by Nakayashiki et al. [53]. Fosmid libraries of O-29J, O-30C, Si-1J, Si-6I, Pm-1J, and Dsa-1J were constructed with CopyControl Fosmid Library Production Kit (EPICENTRE, Madison, Wisconsin) by Takara Bio (Shiga, Japan). Total DNAs were sheared into approximately 40 kb fragments, blunt-ended, and ligated into the pCC1FOS vector. Clones containing *AVR-Pita* homologs were selected from these libraries by colony hybridization. Plasmid clones were constructed by inserting *AVR-Pita*-containing fragments into pBluescript II SK (+) as described in Table S2.

### DNA sequencing

Cosmid, fosmid, and plasmid clones listed in Table S2 were sequenced using the ABI Prism Big Dye Terminator Cycle Sequencing Kit (Applied Biosystems, Foster City, CA) and the ABI 3100 Genetic Analyzer following the manufacturer's instructions. When PCR clones were used as templates, three or more clones from each ligation reaction were sequenced and compared to eliminate PCR errors. For Si(Si-5I) and Sv(Sv-8J) PCR amplicons were directly sequenced using the primers listed in Table S3. The resulting sequences were assembled using SeqManII (DNASTAR, Madison, WI), analyzed with Genetyx version 6.1.1 (GENETYX, Tokyo) and GeneQuest (DNASTAR, Madison, Wisconsin), and aligned with MEGA 4 [92].

### Genetic complementation assay

A total of 13 clones containing *AVR-Pita* homologs were used for the transformation assay (Table 1 and Table S2). For J1(O-29J) and J2(O-29J), fosmid clones with these homologs selected from the O-29J fosmid library were used without subcloning as a negative and positive controls. For Si(Si-6I), Pm(Pm-1J), and D1(Dsa-1J), restriction fragments containing them were isolated from gels, ligated with pBluescript II SK(+), and transformed into *E. coli*. Colonies carrying the target homologs were detected by colony hybridization. For the other 8 homologs PCR cloning was performed. Each of the 8 homologs was amplified from genomic DNA so that the amplicon contained the conserved region, and cloned into pBluescript II SK (+). The 13 plasmid and fosmid clones were introduced into O-8J (P-2b) by co-transformation with pSH75 containing the hygromycin B phosphotransferase gene [93] as described by Tosa et al. [94]. Five transformants carrying full-length transgene(s) were chosen for each homolog through Southern hybridization, and employed for infection assay. Pathogenicity tests were performed as described previously [94].

### Phylogenetic analysis

Two isolates (O-29J and O-30C) were added to the isolates used by Hirata et al. [12], and a phylogenic tree of *Pyricularia* isolates was constructed again as described previously [12]. The phylogenetic tree of *AVR-Pita* homologs was constructed using Bayesian inference in MrBayes v3.1.2 [95]. We chose the general time-reversible model and across-site rate variation for gamma distribution with a proportion of invariant sites for the analysis. Two independent Monte Carlo Markov Chain analyses for 500,000 generations were performed. The temperature for heating the chains was 0.2. We sampled once every 100 generations. After the 500,000 generations, average standard deviation of split frequency was 0.0076, which is acceptable for convergence between trees from the two parallel runs. After the first 1250 trees were discarded, a 50% majority rule consensus tree was constructed based on the remaining samples.

### Accession numbers

Sequence data from this article can be found in the EMBL/GenBank data libraries under accession numbers: AB607333, J1(O-20IN); AB607334, J1(O-29J); AB607335, J1(O-30C); AB607336, J2(O-29J); AB607337, J3(O-2J); AB607338, PO(O-23IN); AB607339, PO(O-20IN); AB607340, Si(Si-6I); AB607341, Sv(Sv-7J); AB607342, Pm(Pm-1J); AB607343, D1(Dsa-1J); AB607344, D1(Dh-5B); AB607345, Ce1(Ce-1B).

## Supporting Information

Figure S1 **Distribution of the** ***AVR-Pita*** **family in** ***Pyricularia*** **isolates.** The RFLP types defined in Figure 1 were plotted on an MP tree constructed from combined data of rDNA, actin, β-tubulin, and calmodulin gene sequences [12]. Bootstrap values >50% are noted at nodes. X, undefined homologs; -, no homologs. Refer to Table S1 and to Hirata et al. [12] for genotypes.
(EPS)

Figure S2 **Chromosomes carrying** ***AVR-Pita*** **homologs in O-23IN and O-20IN retrieved from the 1996 stock.** Chromosomal DNAs were separated by CHEF electrophoresis in the same condition as in Figure 3, blotted, and hybridized with APita766.
(EPS)

Figure S3 **Magnified diagrams of structures around** ***AVR-Pita*** **homologs in representative RFLP types.** Orange/yellow bars and red areas represent the promoter and coding regions, respectively, of the *AVR-Pita* homologs. Gray areas represent syntenic regions. See Figure 6 for legends of symbols.
(EPS)

Table S1 ***Pyricularia*** **isolates used in this study.**
(DOC)

Table S2 ***AVR-Pita*** **clones used in this study.**
(DOC)

Table S3 **Primers used in this study.**
(DOC)

Table S4 **Genetic markers used in this study.**
(DOC)

Dataset S1 **Alignments of exon nucleotide sequences of** ***AVR-Pita*** **homologs used for the construction of** **Figure 7A** **.**
(DOC)
