# Supplementary material for: Multiple Translocation of the AVR-Pita Effector Gene among Chromosomes of the Rice Blast Fungus Magnaporthe oryzae and Related Species
Source: PLoS Pathog. 2011 Jul 28;7(7):e1002147. doi: 10.1371/journal.ppat.1002147 (PMC3145791; doi:10.1371/journal.ppat.1002147)
Supplement: Dataset S1 — Alignments of exon nucleotide sequences of AVR-Pita homologs used for the construction of Figure 7A . (DOC) [file ppat.1002147.s008.doc]

Dataset S1. Alignments of exon nucleotide sequences of *AVR-Pita* homologs used for the construction of Figure 7A.

#J1(70-15) ATG CTT TTT TAT TCA TTG TTA TTT TTT TTT CAC ACC GTT GCG ATT TCG GCC TTC ACC AAC [ 60]

#J1(O-20IN) ATG CTT TTT TAT TCA TTG TTA TTT TTT TTT CAC ACC GTT GCG ATT TCG GCC TTC ACC AAC [ 60]

#J1(O-29J) ATG CTT TTT TAT TCA TTG TTA TTT TTT TTT CAC ACC GTT GCG ATT TCG GCC TTC ACC AAC [ 60]

#J1(O-11J) ATG CTT TTT TAT TCA TTG TTA TTT TTT TTT CAC ACC GTT GCG ATT TCG GCC TTC ACC AAC [ 60]

#J1(O-2J) ATG CTT TTT TAT TCA TTG TTA TTT TTT TTT CAC ACC GTT GCG ATT TCG GCC TTC ACC AAC [ 60]

#J1(O-30C) ATG CTT TTT TAT TCA TTG TTA TTT TTT TTT CAC ACC GTT GCG ATT TCG GCC TTC ACC AAC [ 60]

#J1(O-5J) ATG CTT TTT TAT TCA TTG TTA TTT TTT TTT CAC ACC GTT GCG ATT TCG GCC TTC ACC AAC [ 60]

#J1(O-6J) ATG CTT TTT TAT TCA TTG TTA TTT TTT TTT CAC ACC GTT GCG ATT TCG GCC TTC ACC AAC [ 60]

#J2(O-11J) ATG CTT TTT TAT TCA TTG TTA TTT TTT TTT CAC ACC GTT GCG ATT TCG GCC TTC ACC AAC [ 60]

#J2(O-29J) ATG CTT TTT TAT TCA TTG TTA TTT TTT TTT CAC ACC GTT GCG ATT TCG GCC TTC ACC AAC [ 60]

#J3(O-5J) ATG CTT TTT TAT TCA TTG TTA TTT TTT TTT CAC ACC GTT GCG ATT TCG GCC TTC ACC AAC [ 60]

#J3(O-2J) ATG CTT TTT TAT TCA TTG TTA TTT TTT TTT CAC ACC GTT GCG ATT TCG GCC TTC ACC AAC [ 60]

#PO(O-23IN) ATG CTT TTT TAT TCA TTG TTA TTT TTT TTT CAC ACC GTT GCG ATT TCG GCC TTC ACC AAC [ 60]

#PO(O-20IN) ATG CTT TTT TAT TCA TTG TTA TTT TTT TTT CAC ACC GTT GCG ATT TCG GCC TTC ACC AAC [ 60]

#PO(4224-7-8) ATG CTT TTT TAT TCA --- TTA TTT TTT TTT CAC ACC GTT GCG ATT TCG GCC TTC ACC AAC [ 60]

#Si(Si-5I) ATG CTT TTT TAT TCA TTG TTA TTT TTT TTT CAC ACC GTT GCG ATT TCG GCC TTC ACC AAC [ 60]

#Si(Si-6I) ATG CTT TTT TAT TCA TTG TTA TTT TTT TTT CAC ACC GTT GCG ATT TCG GCC TTC ACC AAC [ 60]

#Sv(Sv-7J) ATG CTT TTT TAT TCA TTG TTA TTT TTT TTT CAC ACC GTT GCG ATT TCG GCC TTC ACC AAC [ 60]

#Sv(Sv-8J) ATG CTT TTT TAT TCA TTG TTA TTT TTT TTT CAC ACC GTT GCG ATT TCG GCC TTC ACC AAC [ 60]

#Pm(Pm-1J) ATG CTT TTT TAT TTA TTT ATA TTT TAT TTT CAC ACC GTT GCA ATT TCG GCC TTC ACC AAC [ 60]

#Pm(Pm-2J) ATG CTT TTT TAT TTA TTT ATA TTT TAT TTT CAC ACC GTT GCA ATT TCG GCC TTC ACC AAC [ 60]

#Pm(Pm-3J) ATG CTT TTT TAT TTA TTT ATA TTT TAT TTT CAC ACC GTT GCA ATT TCG GCC TTC ACC AAC [ 60]

#Pm(Pm-4J) ATG CTT TTT TAT TTA TTT ATA TTT TAT TTT CAC ACC GTT GCA ATT TCG GCC TTC ACC AAC [ 60]

#Pm(Pm-5J) ATG CTT TTT TAT TTA TTT ATA TTT TAT TTT CAC ACC GTT GCA ATT TCG GCC TTC ACC AAC [ 60]

#D1(Dsa-1J) ATG CTT TTT TAT TCA TTT ATA TTT TAT TTT CAC ACC GTT GCA ATT TCG GCC TTC ACC AAC [ 60]

#D1(Dsa-3J) ATG CTT TTT TAT TCA TTT ATA TTT TAT TTT CAC ACC GTT GCA ATT TCG GCC TTC ACC AAC [ 60]

#D1(Dsm-4J) ATG CTT TTT TAT TCA TTT ATA TTT TAT TTT CAC ACC GTT GCA ATT TCG GCC TTC ACC AAC [ 60]

#D1(Dsa-2J) ATG CTT TTT TAT TCA TTT ATA TTT TAT TTT CAC ACC GTT GCA ATT TCG GCC TTC ACC AAC [ 60]

#D1(Dh-5B) ATG CTT TTT TAT TCA TTT ATA TTT TAT TTT CAC ACC GTT GCA ATT TCG GCC TTC ACC AAC [ 60]

#Ce1(Ce-1B) ATG CTT TTC TTA TTC GTT TTC ATT TTT TTT CAC ACC GTT GCA GTT TCG GCC TTC ACT AAC [ 60]

#Ce1(Ecc-1B) ATG CTT TTC TTA TTC GTT TTC ATT TTT TTT CAC ACC GTT GCA GTT TCG GCC TTC ACT AAC [ 60]

#DQ855958 ATG CTT TTT TTA TTT ATT CTC GGT TAC CTT TAT ACC CCT GCA ACT TCG GCC TTT ATA AAT [ 60]

#J1(70-15) ATT GGC ACC TTT TCA CAC CCA GTT TAC GAT TAC AAT CCA ATT CCA AAC CAT ATC CAC GGA [120]

#J1(O-20IN) ATT GGC ACC TTT TCA CAC CCA GTT TAC GAT TAC AAT CCA ATT CCA AAC CAT ATC CAC GGA [120]

#J1(O-29J) ATT GGC ACC TTT TCA CAC CCA GTT TAC GAT TAC AAT CCA ATT CCA AAC CAT ATC CAC GGA [120]

#J1(O-11J) ATT GGC ACC TTT TCA CAC CCA GTT TAC GAT TAC AAT CCA ATT CCA AAC CAT ATC CAC GGA [120]

#J1(O-2J) ATT GGC ACC TTT TCA CAC CCA GTT TAC GAT TAC AAT CCA ATT CCA AAC CAT ATC CAC GGA [120]

#J1(O-30C) ATT GGC ACC TTT TCA CAC CCA GTT TAC GAT TAC AAT CCA ATT CCA AAC CAT ATC CAC GGA [120]

#J1(O-5J) ATT GGC ACC TTT TCA CAC CCA GTT TAC GAT TAC AAT CCA ATT CCA AAC CAT ATC CAC GGA [120]

#J1(O-6J) ATT GGC ACC TTT TCA CAC CCA GTT TAC GAT TAC AAT CCA ATT CCA AAC CAT ATC CAC GGA [120]

#J2(O-11J) ATT GGC ACC TTT TCA CAC CCA GTT TAC GAT TAC AAT CCA ATT CCA AAC CAT ATC CAC GGA [120]

#J2(O-29J) ATT GGC ACC TTT TCA CAC CCA GTT TAC GAT TAC AAT CCA ATT CCA AAC CAT ATC CAC GGA [120]

#J3(O-5J) ATT GGC ACC TTT TCA CAC CCA GTT TAC GAT TAC AAT CCA ATT CCA AAC CAT ATC CAC GGA [120]

#J3(O-2J) ATT GGC ACC TTT TCA CAC CCA GTT TAC GAT TAC AAT CCA ATT CCA AAC CAT ATC CAC GGA [120]

#PO(O-23IN) ATT GGC ACC TTT TCA CAC CCA GTT TAC GAT TAC AAT CCA ATT CCA AAC CAT ATC CAC GGA [120]

#PO(O-20IN) ATT GGC ACC TTT TCA CAC CCA GTT TAC GAT TAC AAT CCA ATT CCA AAC CAT ATC CAC GGA [120]

#PO(4224-7-8) ATT GGC ACC TTT TCA CAC CCA GTT TAC GAT TAC AAT CCA ATT CCA AAC CAT ATC CAC GGA [120]

#Si(Si-5I) ATT GGC ACC TTT TCA CAC CCA GTT TAC GAT TAC AAT CCA ATT CCA AAC CAT ATC CAC GGA [120]

#Si(Si-6I) ATT GGC ACC TTT TCA CAC CCA GTT TAC GAT TAC AAT CCA ATT CCA AAC CAT ATC CAC GGA [120]

#Sv(Sv-7J) ATT GGC ACC TTT TCA CAC CCA GTT TAC GAT TAC AAT CCA ATT CCA AAC CAT ATC CAC GGA [120]

#Sv(Sv-8J) ATT GGC ACC TTT TCA CAC CCA GTT TAC GAT TAC AAT CCA ATT CCA AAC CAT ATC CAC GGA [120]

#Pm(Pm-1J) ATT GGC ACC TTT TCA TAC CCA GTT TAC AAT TCC AAT CCA ATT CCA AAC CAT ATC CAC GGA [120]

#Pm(Pm-2J) ATT GGC ACC TTT TCA TAC CCA GTT TAC AAT TCC AAT CCA ATT CCA AAC CAT ATC CAC GGA [120]

#Pm(Pm-3J) ATT GGC ACC TTT TCA TAC CCA GTT TAC AAT TCC AAT CCA ATT CCA AAC CAT ATC CAC GGA [120]

#Pm(Pm-4J) ATT GGC ACC TTT TCA TAC CCA GTT TAC AAT TCC AAT CCA ATT CCA AAC CAT ATC CAC GGA [120]

#Pm(Pm-5J) ATT GGC ACC TTT TCA TAC CCA GTT TAC AAT TCC AAT CCA ATT CCA AAC CAT ATC CAC GGA [120]

#D1(Dsa-1J) ATT GGC ACC TTT TCA TAC CCA GTT TAC AAT TCC AAT CCA ATT CCA AAC CAT ATC CAC GGA [120]

#D1(Dsa-3J) ATT GGC ACC TTT TCA TAC CCA GTT TAC AAT TCC AAT CCA ATT CCA AAC CAT ATC CAC GGA [120]

#D1(Dsm-4J) ATT GGC ACC TTT TCA TAC CCA GTT TAC AAT TCC AAT CCA ATT CCA AAC CAT ATC CAC GGA [120]

#D1(Dsa-2J) ATT GGC ACC TTT TCA TAC CCA GTT TAC AAT TCC AAT CCA ATT CCA AAC CAT ATC CAC GGA [120]

#D1(Dh-5B) ATT GGC ACC TTT TCA TAC CCA GTT TAC AAT TCC AAT CCA ATT CCA AAC CAT ATC CAC GGA [120]

#Ce1(Ce-1B) ATT GGC ACC TCT TCA TAC CCA ATT TAC AAT TAT AAG CCA ATT CCA AGC CAT ATC CAC GGA [120]

#Ce1(Ecc-1B) ATT GGC ACC TCT TCA TAC CCA ATT TAC AAT TAT AAG CCA ATT CCA AGC CAT ATC CAC GGA [120]

#DQ855958 ACC GGT ACC TTT TTA CAG CCA ATT AAC AAC TAT AAA GAA TTT TCA AAT GAT ATC CAC GGG [120]

#J1(70-15) GAT TTA AAA AGG CGG GCT TAT ATT GAA CGC TAT TCC CAA TGT TCA GAT TCG CAG GCC TCC [180]

#J1(O-20IN) GAT TTA AAA AGG CGG GCT TAT ATT GAA CGC TAT TCC CAA TGT TCA GAT TCG CAG GCC TCC [180]

#J1(O-29J) GAT TTA AAA AGG CGG GCT TAT ATT GAA CGC TAT TCC CAA TGT TCA GAT TCG CAG GCC TCC [180]

#J1(O-11J) GAT TTA AAA AGG CGG GCT TAT ATT GAA CGC TAT TCC CAA TGT TCA GAT TCG CAG GCC TCC [180]

#J1(O-2J) GAT TTA AAA AGG CGG GCT TAT ATT GAA CGC TAT TCC CAA TGT TCA GAT TCG CAG GCC TCC [180]

#J1(O-30C) GAT TTA AAA AGG CGG GCT TAT ATT GAA CGC TAT TCC CAA TGT TCA GAT TCG CAG GCC TCC [180]

#J1(O-5J) GAT TTA AAA AGG CGG GCT TAT ATT GAA CGC TAT TCC CAA TGT TCA GAT TCG CAG GCC TCC [180]

#J1(O-6J) GAT TTA AAA AGG CGG GCT TAT ATT GAA CGC TAT TCC CAA TGT TCA GAT TCG CAG GCC TCC [180]

#J2(O-11J) GAT TTA AAA AGG CGG GCT TAT ATT GAA CGC TAT TCC CAA TGT TCA GAT TCG CAG GCC TCC [180]

#J2(O-29J) GAT TTA AAA AGG CGG GCT TAT ATT GAA CGC TAT TCC CAA TGT TCA GAT TCG CAG GCC TCC [180]

#J3(O-5J) GAT TTA AAA AGG CGG GCT TAT ATT GAA CGC TAT TCC CAA TGT TCA GAT TCG CAG GCC TCC [180]

#J3(O-2J) GAT TTA AAA AGG CGG GCT TAT ATT GAA CGC TAT TCC CAA TGT TCA GAT TCG CAG GCC TCC [180]

#PO(O-23IN) GAT TTA AAA AGG CGG GCT TAT ATT GAA CGC TAT TCC CAA TGT TCA GAT TCG CAG GCC TCC [180]

#PO(O-20IN) GAT TTA AAA AGG CGG GCT TAT ATT GAA CGC TAT TCC CAA TGT TCA GAT TCG CAG GCC TGC [180]

#PO(4224-7-8) GAT TTA AAA AGG CGG GCT TAT ATT GAA CGC TAT TCC CAA TGT TCA GAT TCG CAG GCC TCC [180]

#Si(Si-5I) GAT TTA AAA AGG CGG GCT TAT ATT GAA CGC TAT TCC CAA TGT TCA GAT TCG CAG GCC TCC [180]

#Si(Si-6I) GAT TTA AAA AGG CGG GCT TAT ATT GAA CGC TAT TCC CAA TGT TCA GAT TCG CAG GCC TCC [180]

#Sv(Sv-7J) GAT TTA AAA AGG CGG GCT TAT ATT GAA CGC TAT TCC CAA TGT TCA GAT TCG CAG GCC TCC [180]

#Sv(Sv-8J) GAT TTA AAA AGG CGG GCT TAT ATT GAA CGC TAT TCC CAA TGT TCA GAT TCG CAG GCC TCC [180]

#Pm(Pm-1J) GAT TTA AAA AGG CGG GCT TAT ATT GAA CCC TAT TCC CAA TGT TCA AAT TCG CAG GAC TCC [180]

#Pm(Pm-2J) GAT TTA AAA AGG CGG GCT TAT ATT GAA CCC TAT TCC CAA TGT TCA AAT TCG CAG GAC TCC [180]

#Pm(Pm-3J) GAT TTA AAA AGG CGG GCT TAT ATT GAA CCC TAT TCC CAA TGT TCA AAT TCG CAG GAC TCC [180]

#Pm(Pm-4J) GAT TTA AAA AGG CGG GCT TAT ATT GAA CCC TAT TCC CAA TGT TCA AAT TCG CAG GAC TCC [180]

#Pm(Pm-5J) GAT TTA AAA AGG CGG GCT TAT ATT GAA CCC TAT TCC CAA TGT TCA AAT TCG CAG GAC TCC [180]

#D1(Dsa-1J) GAT TTA AAA AGG CGG GCT TAT ATT GAA CCC TAT TCC CAA TGT TCA AAT TCG CAG GAC TCC [180]

#D1(Dsa-3J) GAT TTA AAA AGG CGG GCT TAT ATT GAA CCC TAT TCC CAA TGT TCA AAT TCG CAG GAC TCC [180]

#D1(Dsm-4J) GAT TTA AAA AGG CGG GCT TAT ATT GAA CCC TAT TCC CAA TGT TCA AAT TCG CAG GAC TCC [180]

#D1(Dsa-2J) GAT TTA AAA AGG CGG GCT TAT ATT GAA CCC TAT TCC CAA TGT TCA AAT TCG CAG GAC TCC [180]

#D1(Dh-5B) GAT TTA AAA AGG CGG GCT TAT ATT GAA CCC TAT TCC CAA TGT TCA AAT TCG CAG GAC TCC [180]

#Ce1(Ce-1B) GTT TTG AGA AAG CGG GCT TAT ATT GAA CGC CAT TCC CAA TGT TCA GAT TCG CAA GCC TCC [180]

#Ce1(Ecc-1B) GTT TTG AGA AAG CGG GCT TAT ATT GAA CGC CAT TCC CAA TGT TCA GAT TCG CAA GCC TCC [180]

#DQ855958 GCT TTA AAT AAA CGG GCT TAT ATA CAA AAA GGT TCT CAA TGC TCG AGT TCG GAA GCC TCG [180]

#J1(70-15) GAA ATT CGT GCC GCG CTA AAA AGT TGC GCC GAG CTC GCC TCG TGG GGC TAT CAC GCC GTT [240]

#J1(O-20IN) GAA ATT CGT GCC GCG CTA AAA AGT TGC GCC GAG CTC GCC TCG TGG GGC TAT CAC GCC GTT [240]

#J1(O-29J) GAA ATT CGT GCC GCG CTA AAA AGT TGC GCC GAG CTC GCC TCG TGG GGC TAT CAC GCC GTT [240]

#J1(O-11J) GAA ATT CGT GCC GCG CTA AAA AGT TGC GCC GAG CTC GCC TCG TGG GGC TAT CAC GCC GTT [240]

#J1(O-2J) GAA ATT CGT GCC GCG CTA AAA AGT TGC GCC GAG CTC GCC TCG TGG GGC TAT CAC GCC GTT [240]

#J1(O-30C) GAA ATT CGT GCC GCG CTA AAA AGT TGC GCC GAG CTC GCC TCG TGG GGC TAT CAC GCC GTT [240]

#J1(O-5J) GAA ATT CGT GCC GCG CTA AAA AGT TGC GCC GAG CTC GCC TCG TGG GGC TAT CAC GCC GTT [240]

#J1(O-6J) GAA ATT CGT GCC GCG CTA AAA AGT TGC GCC GAG CTC GCC TCG TGG GGC TAT CAC GCC GTT [240]

#J2(O-11J) GAA ATT CGT GCC GCG CTA AAA AGT TGC GCC GAG CTC GCC TCG TGG GGC TAT CAC GCC GTT [240]

#J2(O-29J) GAA ATT CGT GCC GCG CTA AAA AGT TGC GCC GAG CTC GCC TCG TGG GGC TAT CAC GCC GTT [240]

#J3(O-5J) GAA ATT CGT GCC GCG CTA AAA AGT TGC GCC GAG CTC GCC TCG TGG GGC TAT CAC GCC GTT [240]

#J3(O-2J) GAA ATT CGT GCC GCG CTA AAA AGT TGC GCC GAG CTC GCC TCG TGG GGC TAT CAC GCC GTT [240]

#PO(O-23IN) GAA ATT CGT GCC GCG CTA AAA AGT TGT GCC GAG CTC GCC TCG TGG GGC TAT CAC GCC GTT [240]

#PO(O-20IN) GAA ATT CGT GCC GCG CTA AAA AGT TGT GCC GAG CTC GCC TCG TGG GGC TAT CAC GCC GTT [240]

#PO(4224-7-8) GAA ATT CGT GCC GCG CTA AAA AGT TGT GCC GAG CTC GCC TCG TGG GGC TAT CAC GCC GTT [240]

#Si(Si-5I) GAA ATT CGT GCC GCG CTA AAA AGT TGT GCC GAG CTC GCC TCG TGG GGC TAT TAC GCC GTT [240]

#Si(Si-6I) GAA ATT CGT GCC GCG CTA AAA AGT TGT GCC GAG CTC GCC TCG TGG GGC TAT TAC GCC GTT [240]

#Sv(Sv-7J) GAA ATT CGT GCC GCG CTA AAA AGT TGT GCC GAG CTC GCC TCG TGG GGC TAT CAC GCC GTT [240]

#Sv(Sv-8J) GAA ATT CGT GCC GCG CTA AAA AGT TGT GCC GAG CTC GCC TCG TGG GGC TAT CAC GCC GTT [240]

#Pm(Pm-1J) GAA ATT CGT GCC GCG CTA AAA AGT TGT GCC GAA CTC GCC TCG TGG GCC TAT CAC GCC GTT [240]

#Pm(Pm-2J) GAA ATT CGT GCC GCG CTA AAA AGT TGT GCC GAA CTC GCC TCG TGG GCC TAT CAC GCC GTT [240]

#Pm(Pm-3J) GAA ATT CGT GCC GCG CTA AAA AGT TGT GCC GAA CTC GCC TCG TGG GCC TAT CAC GCC GTT [240]

#Pm(Pm-4J) GAA ATT CGT GCC GCG CTA AAA AGT TGT GCC GAA CTC GCC TCG TGG GCC TAT CAC GCC GTT [240]

#Pm(Pm-5J) GAA ATT CGT GCC GCG CTA AAA AGT TGT GCC GAA CTC GCC TCG TGG GCC TAT CAC GCC GTT [240]

#D1(Dsa-1J) GAA ATT CGT GCC GCG CTA AAA AGT TGT GCC GAA CTC GCC TCG TGG GCC TAT CAC GCC GTT [240]

#D1(Dsa-3J) GAA ATT CGT GCC GCG CTA AAA AGT TGT GCC GAA CTC GCC TCG TGG GCC TAT CAC GCC GTT [240]

#D1(Dsm-4J) GAA ATT CGT GCC GCG CTA AAA AGT TGT GCC GAA CTC GCC TCG TGG GCC TAT CAC GCC GTT [240]

#D1(Dsa-2J) GAA ATT CGT GCC GCG CTA AAA AGT TGT GCC GAA CTC GCC TCG TGG GCC TAT CAC GCC GTT [240]

#D1(Dh-5B) GAA ATT CGT GCC GCG CTA AAA AGT TGT GCC GAA CTC GCC TCG TGG GCC TAT CAC GCC GTT [240]

#Ce1(Ce-1B) GAA ATT CGT GCC GCG CTA GAG AGT TGT GCC GAG CTC GCC TCG CTG GGC TAT CAC GCC GTT [240]

#Ce1(Ecc-1B) GAA ATT CGT GCC GCG CTA GAG AGT TGT GCC GAG CTC GCC TCG CTG GGC TAT CAC GCC GTT [240]

#DQ855958 GAA ATC CGT GGC GCG CTA GAG AGT TGT GCC GTG CTT GCC CAA CAT GCC TTC CAT GCG GTT [240]

#J1(70-15) AAA AGT AAC AAT CGG TTA TTT AAA TTA ATC TTT AAA ACT GAC AGC ACA GAT ATT CAA AAC [300]

#J1(O-20IN) AAA AGT AAC AAT CGG TTA TTT AAA TTA ATC TTT AAA ACT GAC AGC ACA GAT ATT CAA AAC [300]

#J1(O-29J) AAA AGT AAC AAT CGG TTA TTT AAA TTA ATC TTT AAA ACT GAC AGC ACA GAT ATT CAA AAC [300]

#J1(O-11J) AAA AGT AAC AAT CGG TTA TTT AAA TTA ATC TTT AAA ACT GAC AGC ACA GAT ATT CAA AAC [300]

#J1(O-2J) AAA AGT AAC AAT CGG TTA TTT AAA TTA ATC TTT AAA ACT GAC AGC ACA GAT ATT CAA AAC [300]

#J1(O-30C) AAA AGT AAC AAT CGG TTA TTT AAA TTA ATC TTT AAA ACT GAC AGC ACA GAT ATT CAA AAC [300]

#J1(O-5J) AAA AGT AAC AAT CGG TTA TTT AAA TTA ATC TTT AAA ACT GAC AGC ACA GAT ATT CAA AAC [300]

#J1(O-6J) AAA AGT AAC AAT CGG TTA TTT AAA TTA ATC TTT AAA ACT GAC AGC ACA GAT ATT CAA AAC [300]

#J2(O-11J) AAA AGT GAC AAT CGG TTA TTT AAA TTA ATC TTT AAA ACT GAC AGC ACA GAT ATT CAA AAC [300]

#J2(O-29J) AAA AGT GAC AAT CGG TTA TTT AAA TTA ATC TTT AAA ACT GAC AGC ACA GAT ATT CAA AAC [300]

#J3(O-5J) AAA AGT GAC AAT CGG TTA TTT AAA TTA ATC TTT AAA ACT GAC AGC ACA GAT ATT CAA AAC [300]

#J3(O-2J) AAA AGT GAC AAT CGG TTA TTT AAA TTA ATC TTT AAA ACT GAC AGC ACA GAT ATT CAA AAC [300]

#PO(O-23IN) AAA AAT GAC AAT CGG TTA TTT AGA TTA ATC TTT AAA ACT GAC AGC ACA GAT ATT CAA AAC [300]

#PO(O-20IN) AAA AAT GAC AAT CGG TTA TTT AGA TTA ATC TTT AAA ACT GAC AGC ACA GAT ATT CAA AAC [300]

#PO(4224-7-8) AAA AAT GAC AAT CGG TTA TTT AGA TTA ATC TTT AAA ACT GAC AGC ACA GAT ATT CAA AAC [300]

#Si(Si-5I) AAA AGT GAC AAT CGG TTA TTT GAA TTA ATC TTT AAA ACT GAC AGC ACA GAT ATT CAA AAC [300]

#Si(Si-6I) AAA AGT GAC AAT CGG TTA TTT GAA TTA ATC TTT AAA ACT GAC AGC ACA GAT ATT CAA AAC [300]

#Sv(Sv-7J) AAA AGT GAC AAT CGG TTA TTT GAA TTA ATC TTT AAA ACT GAC AGC ACA TAT ATT CAA AAC [300]

#Sv(Sv-8J) AAA AGT GAC AAT CGG TTA TTT GAA TTA ATC TTT AAA ACT GAC AGC ACA TAT ATT CAA AAC [300]

#Pm(Pm-1J) GAA AAT GAC AAT CGG TTA TTT GAA TTG ATT TTT AAA ACT GAC AGC ACA AAT ATT AAA AAC [300]

#Pm(Pm-2J) GAA AAT GAC AAT CGG TTA TTT GAA TTG ATT TTT AAA ACT GAC AGC ACA AAT ATT AAA AAC [300]

#Pm(Pm-3J) GAA AAT GAC AAT CGG TTA TTT GAA TTG ATT TTT AAA ACT GAC AGC ACA AAT ATT AAA AAC [300]

#Pm(Pm-4J) GAA AAT GAC AAT CGG TTA TTT GAA TTG ATT TTT AAA ACT GAC AGC ACA AAT ATT AAA AAC [300]

#Pm(Pm-5J) GAA AAT GAC AAT CGG TTA TTT GAA TTG ATT TTT AAA ACT GAC AGC ACA AAT ATT AAA AAC [300]

#D1(Dsa-1J) GAA AAT GAC AAT CGG TTA TTT GAA TTG ATT TTT AAA ACT GAC AGC ACA AAT ATT AAA AAC [300]

#D1(Dsa-3J) GAA AAT GAC AAT CGG TTA TTT GAA TTG ATT TTT AAA ACT GAC AGC ACA AAT ATT AAA AAC [300]

#D1(Dsm-4J) GAA AAT GAC AAT CGG TTA TTT GAA TTG ATT TTT AAA ACT GAC AGC ACA AAT ATT AAA AAC [300]

#D1(Dsa-2J) GAA AAT GAC AAT CGG TTA TTT GAA TTG ATT TTT AAA ACT GAC AGC ACA AAT ATT AAA AAC [300]

#D1(Dh-5B) GAA AAT GAC AAT CGG TTA TTT GAA TTG ATT TTT AAA ACT GAC AGC ACA AAT ATT AAA AAC [300]

#Ce1(Ce-1B) AAA AGT GAC AAT CGG TTA TTT CAA TTA ATT TTT AAA ACT GAC CGC ACG GAT AGT AAA GAC [300]

#Ce1(Ecc-1B) AAA AGT GAC AAT CGG TTA TTT CAA TTA ATT TTT AAA ACT GAC CGC ACG GAT AGT AAA GAC [300]

#DQ855958 AAA AGC GAT GAG GAA TTA TTC GAG TTC ATT TTC AAA ACC GAC AGC ACA GAT ATT CAA GAC [300]

#J1(70-15) TGG GTT CAA AAT AAT TTT AAC GAA ATT TAC AAG GAA TGT AAC AGG GAC GCG GAC GAA ATT [360]

#J1(O-20IN) TGG GTT CAA AAT AAT TTT AAC GAA ATT TAC AAG GAA TGT AAC AGG GAC GCG GAC GAA ATT [360]

#J1(O-29J) TGG GTT CAA AAT AAT TTT AAC GAA ATT TAC AAG GAA TGT AAC AGG GAC GCG GAC GAA ATT [360]

#J1(O-11J) TGG GTT CAA AAT AAT TTT AAC GAA ATT TAC AAG GAA TGT AAC AGG GAC GCG GAC GAA ATT [360]

#J1(O-2J) TGG GTT CAA AAT AAT TTT AAC GAA ATT TAC AAG GAA TGT AAC AGG GAC GCG GAC GAA ATT [360]

#J1(O-30C) TGG GTT CAA AAT AAT TTT AAC GAA ATT TAC AAG GAA TGT AAC AGG GAC GCG GAC GAA ATT [360]

#J1(O-5J) TGG GTT CAA AAT AAT TTT AAC GAA ATT TAC AAG GAA TGT AAC AGG GAC GCG GAC GAA ATT [360]

#J1(O-6J) TGG GTT CAA AAT AAT TTT AAC GAA ATT TAC AAG GAA TGT AAC AGG GAC GCG GAC GAA ATT [360]

#J2(O-11J) TGG GTT CAA AAT AAT TTT AAC GAA ATT TAC AAG GAA TGT AAC AGG GAC GCG GAC GAA ATT [360]

#J2(O-29J) TGG GTT CAA AAT AAT TTT AAC GAA ATT TAC AAG GAA TGT AAC AGG GAC GCG GAC GAA ATT [360]

#J3(O-5J) TGG GTT CAA AAT AAT TTT AAC GAA ATT TAC AAG GAA TGT AAC AGG GAC GCG GAC GAA ATT [360]

#J3(O-2J) TGG GTT CAA AAT AAT TTT AAC GAA ATT TAC AAG GAA TGT AAC AGG GAC GCG GAC CAA ATT [360]

#PO(O-23IN) TGG GTT CAA AAG AAT TTT AAC GAA ATT TAC AAG GAA TGT AAC AGG GAC GCG GAC GAA ATT [360]

#PO(O-20IN) TGG GTT CAA AAG AAT TTT AAC GAA ATT TAC AAG GAA TGT AAC AGG GAC GCG GAC GAA ATT [360]

#PO(4224-7-8) TGG GTT CAA AAG AAT TTT AAC GAA ATT TAC AAG GAA TGT AAC AGG GAC GCG GAC GAA ATT [360]

#Si(Si-5I) TGG GTT CAA AAT AAT TTT AAC GAA ATT TAC AAG GAA TGT AAC AGG GAC GCG GAC GAA ATT [360]

#Si(Si-6I) TGG GTT CAA AAT AAT TTT AAC GAA ATT TAC AAG GAA TGT AAC AGG GAC GCG GAC GAA ATT [360]

#Sv(Sv-7J) TGG GTT CAA AAT AAT TTT AAC GAA ATT TAC AAG GAA TGT AAC AGG GAC GCG GAC GAA ATT [360]

#Sv(Sv-8J) TGG GTT CAA AAT AAT TTT AAC GAA ATT TAC AAG GAA TGT AAC AGG GAC GCG GAC GAA ATT [360]

#Pm(Pm-1J) TGG GTT CAA AAT AAT TTT AAC GAA ATT TAC AAG GAA TGT AAC AGG GAC GCG GAC GAA ATT [360]

#Pm(Pm-2J) TGG GTT CAA AAT AAT TTT AAC GAA ATT TAC AAG GAA TGT AAC AGG GAC GCG GAC GAA ATT [360]

#Pm(Pm-3J) TGG GTT CAA AAT AAT TTT AAC GAA ATT TAC AAG GAA TGT AAC AGG GAC GCG GAC GAA ATT [360]

#Pm(Pm-4J) TGG GTT CAA AAT AAT TTT AAC GAA ATT TAC AAG GAA TGT AAC AGG GAC GCG GAC GAA ATT [360]

#Pm(Pm-5J) TGG GTT CAA AAT AAT TTT AAC GAA ATT TAC AAG GAA TGT AAC AGG GAC GCG GAC GAA ATT [360]

#D1(Dsa-1J) TGG GTT CAA AAT AAT TTT AAC GAA ATT CAC AAG GAA TGT AAC AGG GAC GCG GAC GAA ATT [360]

#D1(Dsa-3J) TGG GTT CAA AAT AAT TTT AAC GAA ATT CAC AAG GAA TGT AAC AGG GAC GCG GAC GAA ATT [360]

#D1(Dsm-4J) TGG GTT CAA AAT AAT TTT AAC GAA ATT CAC AAG GAA TGT AAC AGG GAC GCG GAC GAA ATT [360]

#D1(Dsa-2J) TGG GTT CAA AAT AAT TTT AAC GAA ATT TAC AAG GAA TGT AAC AGG GAC GCG GAC GAA ATT [360]

#D1(Dh-5B) TGG GTT CAA AAT AAT TTT AAC GAA ATT TAC AAG GAA TGT AAC AGG GAC GCG GAC GAA ATT [360]

#Ce1(Ce-1B) TTT GTT CAA AAC AAT TTC AAC AAA ATC TAC CAG GAA TGT AAG AGG GAC GCG GAC GAA ATT [360]

#Ce1(Ecc-1B) TTT GTT CAA AAC AAT TTC AAC AAA ATC TAC CAG GAA TGT AAG AGG GAC GCG GAC GAA ATT [360]

#DQ855958 CTT GTG GAA CAG AAC TTT AAG AAA CTT TAC GAA GAA TGT AGT AGA AAA GAG GAC GAA GTT [360]

#J1(70-15) TCT CTA ACC TGC CAC GAT AAA AAT GTT TAT ACG TGC GTC CGA GAA GGA GTT CAT --- AAT [420]

#J1(O-20IN) TCT CTA ACC TGC CAC GAT AAA AAT GTT TAT ACG TGC GTC CGA GAA GGA GTT CAT --- AAT [420]

#J1(O-29J) TCT CTA ACC TGC CAC GAT AAA AAT GTT TAT ACG TGC GTC CGA GAA GAA GTT CAT --- AAT [420]

#J1(O-11J) TCT CTA ACC TGC CAC GAT AAA AAT GTT TAT ACG TGC GTC CGA GAA GAA GTT CAT --- AAT [420]

#J1(O-2J) TCT CTA ACC TGC CAC GAT AAA AAT GTT TAT ACG TGC GTC CGA GAA GAA GTT CAT --- AAT [420]

#J1(O-30C) TCT CTA ACC TGC CAC GAT AAA AAT GTT TAT ACG TGC GTC CGA GAA GAA GTT CAT --- AAT [420]

#J1(O-5J) TCT CTA ACC TGC CAC GAT AAA AAT GTT TAT ACG TGC GTC CGA GAA GAA GTT CAT --- AAT [420]

#J1(O-6J) TCT CTA ACC TGC CAC GAT AAA AAT GTT TAT ACG TGC GTC CGA GAA GAA GTT CAT --- AAT [420]

#J2(O-11J) TCT CTA ACC TGC CAC GAT AAA AAT GTT TAT ACG TGC GTC CGA GAA GAA GTT CAT --- AAT [420]

#J2(O-29J) TCT CTA ACC TGC CAC GAT AAA AAT GTT TAT ACG TGC GTC CGA GAA GAA GTT CAT --- AAT [420]

#J3(O-5J) TCT CTA ACC TGC CAC GAT AAA AAT GTT TAT ACG TGC GTC CGA GAA GAA GTT CAT --- AAT [420]

#J3(O-2J) TCT CTA ACC TGC CAC GAT AAA AAT GTT TAT ACG TGC GTC CGA GAA GAA GTT CAT --- AAT [420]

#PO(O-23IN) TCT CTA ACC TGC CAC GAT AAA AAT GTT TAT ACG TGC GTC CGA GAA GGA GTT CAT --- AAT [420]

#PO(O-20IN) TCT CTA ACC TGC CAC GAT AAA AAT GTT TAT ACG TGC GTC CGA GAA GGA GTT CAT --- AAT [420]

#PO(4224-7-8) TCT CTA ACC TGC CAC GAT AAA AAT GTT TAT ACG TGC GTC CGA GAA GGA GTT CAT --- AAT [420]

#Si(Si-5I) TCT CTA ACC TGC CAC GAT AAA GGT GTT TAT AAG TGC GTC CGA GAA GAA GTT CAT --- AAT [420]

#Si(Si-6I) TCT CTA ACC TGC CAC GAT AAA GGT GTT TAT AAG TGC GTC CGA GAA GAA GTT CAT --- AAT [420]

#Sv(Sv-7J) TCT CTA ACC TGC CAC GAT AAA GAT GTT TAT ACG TGC GTC CGA GAA GAA GTT CAT --- AGT [420]

#Sv(Sv-8J) TCT CTA ACC TGC CAC GAT AAA GAT GTT TAT ACG TGC GTC CGA GAA GAA GTT CAT --- AGT [420]

#Pm(Pm-1J) TCT CTA TCC TGC CAC GAT ACA AGT GTT TAT ACG TGC GTC CGA GAA GGA GTT CAT --- CTT [420]

#Pm(Pm-2J) TCT CTA TCC TGC CAC GAT ACA AGT GTT TAT ACG TGC GTC CGA GAA GGA GTT CAT --- CTT [420]

#Pm(Pm-3J) TCT CTA TCC TGC CAC GAT ACA AGT GTT TAT ACG TGC GTC CGA GAA GGA GTT CAT --- CTT [420]

#Pm(Pm-4J) TCT CTA TCC TGC CAC GAT ACA AGT GTT TAT ACG TGC GTC CGA GAA GGA GTT CAT --- CTT [420]

#Pm(Pm-5J) TCT CTA TCC TGC CAC GAT ACA AGT GTT TAT ACG TGC GTC CGA GAA GGA GTT CAT --- CTT [420]

#D1(Dsa-1J) TCT CTA TCC TGC CAC GAT ACA AGT GTT TAT ACG TGC GTC CGA GAA GGA GTT CAT --- CTT [420]

#D1(Dsa-3J) TCT CTA TCC TGC CAC GAT ACA AGT GTT TAT ACG TGC GTC CGA GAA GGA GTT CAT --- CTT [420]

#D1(Dsm-4J) TCT CTA TCC TGC CAC GAT ACA AGT GTT TAT ACG TGC GTC CGA GAA GGA GTT CAT --- CTT [420]

#D1(Dsa-2J) TCT CTA TCC TGC CAC GAT ACA AGT GTT TAT ACG TGC GTC CGA GAA GGA GTT CAT --- CTT [420]

#D1(Dh-5B) TCT CTA TCC TGC CAC GAT ACA AGT GTT TAT ACG TGC GTC CGA GAA GGA GTT CAT --- CTT [420]

#Ce1(Ce-1B) TCT TTA TCC TGC CGC GAT ACA AGT GTT TAT ACG TGC GTC CGA GAT GGA GTT CAT --- ATG [420]

#Ce1(Ecc-1B) TCT TTA TCC TGC CGC GAT ACA AGT GTT TAT ACG TGC GTC CGA GAT GGA GTT CAT --- ATG [420]

#DQ855958 TAT ATC ACC TGC GAA GAT GAA ACT GGA AAG TGT AAA GAA AAT CAA GGA TAC AAA AAC GTT [420]

#J1(70-15) TTG GCG TAT GCA CTT ATT AAC GAA AAA GAA ATT GTT ATA TGC CCT CCT TTC TTC AAC AAC [480]

#J1(O-20IN) TTG GCG TAT GCA CTT ATT AAC GAA AAA GAA ATT GTT ATA TGC CCT CCT TTC TTC AAC AAC [480]

#J1(O-29J) TTG GCG TAT GCA CTT ATT AAC GAA AAA GAA ATT GTT ATA TGC CCT CCT TTC TTC AAC AAC [480]

#J1(O-11J) TTG GCG TAT GCA CTT ATT AAC GAA AAA GAA ATT GTT ATA TGC CCT CCT TTC TTC AAC AAC [480]

#J1(O-2J) TTG GCG TAT GCA CTT ATT AAC GAA AAA GAA ATT GTT ATA TGC CCT CCT TTC TTC AAC AAC [480]

#J1(O-30C) TTG GCG TAT GCA CTT ATT AAC GAA AAA GAA ATT GTT ATA TGC CCT CCT TTC TTC AAC AAC [480]

#J1(O-5J) TTG GCG TAT GCA CTT ATT AAC GAA AAA GAA ATT GTT ATA TGC CCT CCT TTC TTC AAC AAC [480]

#J1(O-6J) TTG GCG TAT GCA CTT ATT AAC GAA AAA GAA ATT GTT ATA TGC CCT CCT TTC TTC AAC AAC [480]

#J2(O-11J) TTG GCG TAT GCA CTT ATT AAC GAA AAA GAA ATT GTT ATA TGC CCT CCT TTC TTC AAC AAC [480]

#J2(O-29J) TTG GCG TAT GCA CTT ATT AAC GAA AAA GAA ATT GTT ATA TGC CCT CCT TTC TTC AAC AAC [480]

#J3(O-5J) TTG GCG TAT GCA CTT ATT AAC GAA AAA GAA ATT GTT ATA TGC CCT CCT TTC TTC AAC AAC [480]

#J3(O-2J) TTG GCG TAT GCA CTT ATT AAC GAA AAA GAA ATT GTT ATA TGC CCT CCT TTC TTC AAC AAC [480]

#PO(O-23IN) TTG GCG TAT GCA CTT ATT AAC GAA AAA GAA ATT GTT ATA TGC CCT CCT TTC TTC AAC AAC [480]

#PO(O-20IN) TTG GCG TAT GCA CTT ATT AAC GAA AAA GAA ATT GTT ATA TGC CCT CCT TTC TTC AAC AAC [480]

#PO(4224-7-8) TTG GCG TAT GCA CTT ATT AAC GAA AAA GAA ATT GTT ATA TGC CCT CCT TTC TTC AAC AAC [480]

#Si(Si-5I) TTG GCG TAT GCA CGC ATT AAC GAA AAA GAA ATT GTT ATA TGC CCT CGT TTC TTC AAC AAG [480]

#Si(Si-6I) TTG GCG TAT GCA CGC ATT AAC GAA AAA GAA ATT GTT ATA TGC CCT CGT TTC TTC AAC AAG [480]

#Sv(Sv-7J) TTG GCG TAT GCA CTT ATT AAC GAA AAA GAA ATT GTT ATA TGC CCT CGT TTC TTC AAC CAC [480]

#Sv(Sv-8J) TTG GCG TAT GCA CTT ATT AAC GAA AAA GAA ATT GTT ATA TGC CCT CGT TTC TTC AAC CAC [480]

#Pm(Pm-1J) TTG GGC TAT GCA AAG ATG TAC GAA AAA CAA GTT GTT TTA TGC CCT CAT TTC TTT GAT CAC [480]

#Pm(Pm-2J) TTG GGC TAT GCA AAG ATG TAC GAA AAA CAA GTT GTT TTA TGC CCT CAT TTC TTT GAT CAC [480]

#Pm(Pm-3J) TTG GGC TAT GCA AAG ATG TAC GAA AAA CAA GTT GTT TTA TGC CCT CAT TTC TTT GAT CAC [480]

#Pm(Pm-4J) TTG GGC TAT GCA AAG ATG TAC GAA AAA CAA GTT GTT TTA TGC CCT CAT TTC TTT GAT CAC [480]

#Pm(Pm-5J) TTG GGC TAT GCA AAG ATG TAC GAA AAA CAA GTT GTT TTA TGC CCT CAT TTC TTT GAT CAC [480]

#D1(Dsa-1J) TTG GGC TAT GCA AAG ATG TAC GAA AAA CAA GTT GTT TTA TGC CCT CAT TTC TTT GAT CAC [480]

#D1(Dsa-3J) TTG GGC TAT GCA AAG ATG TAC GAA AAA CAA GTT GTT TTA TGC CCT CAT TTC TTT GAT CAC [480]

#D1(Dsm-4J) TTG GGC TAT GCA AAG ATG TAC GAA AAA CAA GTT GTT TTA TGC CCT CAT TTC TTT GAT CAC [480]

#D1(Dsa-2J) TTG GGC TAT GCA AAG ATG TAC GAA AAA CAA GTT GTT TTA TGC CCT CAT TTC TTT GAT CAC [480]

#D1(Dh-5B) TTG GGC TAT GCA AAG ATG TAC GAA AAA CAA GTT GTT TTA TGC CCT CAT TTC TTT GAT CAC [480]

#Ce1(Ce-1B) ATG GGC TAT GCA AAT ATC CAC GAA AAA CAA ATT GTG GTA TGC CCT ATA TTC TTC AAT GCC [480]

#Ce1(Ecc-1B) ATG GGC TAT GCA AAT ATC CAC GAA AAA CAA ATT GTG GTA TGC CCT ATA TTC TTC AAT GCC [480]

#DQ855958 TTG GGC TAC GCA AGA ATT GCA AAA GAA CAA ATT GTT ATA TGC CCC AAT TTC TTC AAT CAA [480]

#J1(70-15) CCC GTA AAC AGC AGG GAA ATT ACT GCC GGT AAC CAA GAT ACA ATT ATA TTA CAT GAA ATG [540]

#J1(O-20IN) CCC GTA AAC AGC AGG GAA ATT ACT GCC GGT AAC CAA GAT ACA ATT ATA TTA CAT GAA ATG [540]

#J1(O-29J) CCC GTA AAC AGC AGG GAA ATT ACT GCC GGT AAC CAA GAT ACA ATT ATA TTA CAT GAA ATG [540]

#J1(O-11J) CCC GTA AAC AGC AGG GAA ATT ACT GCC GGT AAC CAA GAT ACA ATT ATA TTA CAT GAA ATG [540]

#J1(O-2J) CCC GTA AAC AGC AGG GAA ATT ACT GCC GGT AAC CAA GAT ACA ATT ATA TTA CAT GAA ATG [540]

#J1(O-30C) CCC GTA AAC AGC AGG GAA ATT ACT GCC GGT AAC CAA GAT ACA ATT ATA TTA CAT GAA ATG [540]

#J1(O-5J) CCC GTA AAC AGC AGG GAA ATT ACT GCC GGT AAC CAA GAT ACA ATT ATA TTA CAT GAA ATG [540]

#J1(O-6J) CCC GTA AAC AGC AGG GAA ATT ACT GCC GGT AAC CAA GAT ACA ATT ATA TTA CAT GAA ATG [540]

#J2(O-11J) CCC GTA AAC AGC AGG GAA ATT ACT GCC GGT AAC CAA GAT ACA ATT ATA TTA CAT GAA ATG [540]

#J2(O-29J) CCC GTA AAC AGC AGG GAA ATT ACT GCC GGT AAC CAA GAT ACA ATT ATA TTA CAT GAA ATG [540]

#J3(O-5J) CCC GTA AAC AGC AGG GAA ATT ACT GCC GGT AAC CAA GAT ACA ATT ATA TTA CAT GAA ATG [540]

#J3(O-2J) CCC GTA AAC AGC AGG GAA ATT ACT GCC GGT AAC CAA GAT ACA ATT ATA TTA CAT GAA ATG [540]

#PO(O-23IN) CCC GTA AAC AGC AGG GAA ATT ACT GCC GGT AAC CAA GAT ACA GTT ATA TTA CAT GAA ATG [540]

#PO(O-20IN) CCC GTA AAC AGC AGG GAA ATT ACT GCC GGT AAC CAA GAT ACA GTT ATA TTA CAT GAA ATG [540]

#PO(4224-7-8) CCC GTA AAC AGC AGG GAA ATT ACT GCC GGT AAC CAA GAT ACA GTT ATA TTA CAT GAA ATG [540]

#Si(Si-5I) CCC GTA AAC AGC AGG GAA ATT ACT GCC GGT AAC CAA GAT ACA GTT ATA TTA CAT GAA ATG [540]

#Si(Si-6I) CCC GTA AAC AGC AGG GAA ATT ACT GCC GGT AAC CAA GAT ACA GTT ATA TTA CAT GAA ATG [540]

#Sv(Sv-7J) CCC GTA AAC AGC AGG GAA ATT ACT GCC GGT AAC CAA GAT ACA GTT ATA TTA CAT GAA ATG [540]

#Sv(Sv-8J) CCC GTA AAC AGC AGG GAA ATT ACT GCC GGT AAC CAA GAT ACA GTT ATA TTA CAT GAA ATG [540]

#Pm(Pm-1J) CCC GTA AAC AGC AGG GAA ATC ACT GCC CAA AAC CAA GAT ACA GTT ATA TTG CAT GAA ATG [540]

#Pm(Pm-2J) CCC GTA AAC AGC AGG GAA ATC ACT GCC CAA AAC CAA GAT ACA GTT ATA TTG CAT GAA ATG [540]

#Pm(Pm-3J) CCC GTA AAC AGC AGG GAA ATC ACT GCC CAA AAC CAA GAT ACA GTT ATA TTG CAT GAA ATG [540]

#Pm(Pm-4J) CCC GTA AAC AGC AGG GAA ATC ACT GCC CAA AAC CAA GAT ACA GTT ATA TTG CAT GAA ATG [540]

#Pm(Pm-5J) CCC GTA AAC AGC AGG GAA ATC ACT GCC CAA AAC CAA GAT ACA GTT ATA TTG CAT GAA ATG [540]

#D1(Dsa-1J) CCC GTA AAC AGC AGG GAA ATC ACT GCC CAA AAC CAA GAT ACA GTT ATA TTG CAT GAA ATG [540]

#D1(Dsa-3J) CCC GTA AAC AGC AGG GAA ATC ACT GCC CAA AAC CAA GAT ACA GTT ATA TTG CAT GAA ATG [540]

#D1(Dsm-4J) CCC GTA AAC AGC AGG GAA ATC ACT GCC CAA AAC CAA GAT ACA GTT ATA TTG CAT GAA ATG [540]

#D1(Dsa-2J) CCC GTA AAC AGC AGG GAA ATC ACT GCC CAA AAC CAA GAT ACA GTT ATA TTG CAT GAA ATG [540]

#D1(Dh-5B) CCC GTA AAC AGC AGG GAA ATC ACT GCC CAA AAC CAA GAT ACA GTT ATA TTG CAT GAA ATG [540]

#Ce1(Ce-1B) CCA GTA AGC AGC AGG AGA ATT ACT GCA AGC AAC CAA GAT ACA GCT ATA TTA CAT GAA ATG [540]

#Ce1(Ecc-1B) CCA GTA AGC AGC AGG AGA ATT ACT GCA AGC AAC CAA GAT ACA GCT ATA TTA CAT GAA ATG [540]

#DQ855958 CCC GAG AGC AGC AGT GAA ATT ACT GCA ACC AAC CAA GAT ACT ACG ATT ATG CAT GAA CTA [540]

#J1(70-15) GTG CAT ATA ATT TTA AAA GAG TGG AAA GAT TAT GGT TGC GAA TGG GAT GGG ATT CAC AAA [600]

#J1(O-20IN) GTG CAT ATA ATT TTA AAA GAG TGG AAA GAT TAT GGT TGC GAA TGG GAT GGG ATT CAC AAA [600]

#J1(O-29J) GTG CAT ATA ATT TTA AAA GAG TGG AAA GAT TAT GGT TGC GAA TGG GAT GGG ATT CAC AAA [600]

#J1(O-11J) GTG CAT ATA ATT TTA AAA GAG TGG AAA GAT TAT GGT TGC GAA TGG GAT GGG ATT CAC AAA [600]

#J1(O-2J) GTG CAT ATA ATT TTA AAA GAG TGG AAA GAT TAT GGT TGC GAA TGG GAT GGG ATT CAC AAA [600]

#J1(O-30C) GTG CAT ATA ATT TTA AAA GAG TGG AAA GAT TAT GGT TGC GAA TGG GAT GGG ATT CAC AAA [600]

#J1(O-5J) GTG CAT ATA ATT TTA AAA GAG TGG AAA GAT TAT GGT TGC GAA TGG GAT GGG ATT CAC AAA [600]

#J1(O-6J) GTG CAT ATA ATT TTA AAA GAG TGG AAA GAT TAT GGT TGC GAA TGG GAT GGG ATT CAC AAA [600]

#J2(O-11J) GTG CAT ATA ATT TTA AAA GAG TGG AAA GAT TAT GGT TAC GAA TGG GAT GGG ATT CAC AAA [600]

#J2(O-29J) GTG CAT ATA ATT TTA AAA GAG TGG AAA GAT TAT GGT TAC GAA TGG GAT GGG ATT CAC AAA [600]

#J3(O-5J) GTG CAT ATA ATT TTA AAA GAG TGG AAA GAT TAT GGT TAC GAA TGG GAT GGG ATT CAC AAA [600]

#J3(O-2J) GTG CAT ATA ATT TTA AAA GAG TGG AAA GAT TAT GGT TAC GAA TGG GAT GGG ATT CAC AAA [600]

#PO(O-23IN) GTG CAT ATA ATT TTA AAA GAG TGG AAA GAT TAT GGT TGC GAA TGG CAT GGG ATT CAC AAA [600]

#PO(O-20IN) GTG CAT ATA ATT TTA AAA GAG TGG AAA GAT TAT GGT TGC GAA TGG CAT GGG ATT CAC AAA [600]

#PO(4224-7-8) GTG CAT ATA ATT TTA AAA GAG TGG AAA GAT TAT GGT TAC GAA TGG GAT GGG ATT CAC AAA [600]

#Si(Si-5I) GTG CAT ATA ATT TTA AAA GAG TGG AAA GAT TAT GGT TCC GAA TGG GGT GGG ATT CAC AAA [600]

#Si(Si-6I) GTG CAT ATA ATT TTA AAA GAG TGG AAA GAT TAT GGT TCC GAA TGG GGT GGG ATT CAC AAA [600]

#Sv(Sv-7J) GTG CAT ATA ATT TTA AAA CAG TGG AAA GAT TAT GGT TAC GAA TGG GAT GGG ATT CAC AAA [600]

#Sv(Sv-8J) GTG CAT ATA ATT TTA AAA CAG TGG AAA GAT TAT GGT TAC GAA TGG GAT GGG ATT CAC AAA [600]

#Pm(Pm-1J) CTG CAT ATA ATT CTA AAT GAG TGG GAA GAT TAT GGT TAC GAA TGG GAT GGG ATT CAC AAT [600]

#Pm(Pm-2J) CTG CAT ATA ATT CTA AAT GAG TGG GAA GAT TAT GGT TAC GAA TGG GAT GGG ATT CAC AAT [600]

#Pm(Pm-3J) CTG CAT ATA ATT CTA AAT GAG TGG GAA GAT TAT GGT TAC GAA TGG GAT GGG ATT CAC AAT [600]

#Pm(Pm-4J) CTG CAT ATA ATT CTA AAT GAG TGG GAA GAT TAT GGT TAC GAA TGG GAT GGG ATT CAC AAT [600]

#Pm(Pm-5J) CTG CAT ATA ATT CTA AAT GAG TGG GAA GAT TAT GGT TAC GAA TGG GAT GGG ATT CAC AAT [600]

#D1(Dsa-1J) CTG CAT ATA ATT CTA AAT GAG TGG GAA GAT TAT GGT TAC GAA TGG GAT GGG ATT CAC AAT [600]

#D1(Dsa-3J) CTG CAT ATA ATT CTA AAT GAG TGG GAA GAT TAT GGT TAC GAA TGG GAT GGG ATT CAC AAT [600]

#D1(Dsm-4J) CTG CAT ATA ATT CTA AAT GAG TGG GAA GAT TAT GGT TAC GAA TGG GAT GGG ATT CAC AAT [600]

#D1(Dsa-2J) CTG CAT ATA ATT CTA AAT GAG TGG GAA GAT TAT GGT TAC GAA TGG GAT GGG ATT CAC AAT [600]

#D1(Dh-5B) CTG CAT ATA ATT CTA AAT GAG TGG GAA GAT TAT GGT TAC GAA TGG GAT GGG ATT CAC AAT [600]

#Ce1(Ce-1B) CTG CAT ATA ATT TTA AAT GAG TGG GAA GAT TAT GGT TAC GAA TGG GAT GAG ATT CAC AGA [600]

#Ce1(Ecc-1B) CTG CAT ATA ATT TTA AAT GAG TGG GAA GAT TAT GGT TAC GAA TGG GAT GAG ATT CAC AGA [600]

#DQ855958 GCG CAT ATC ATT TTA GAT CTC CCT GAA GAC TTT GGC TAC GAA TGG ACG GGA GTT CAT AAG [600]

#J1(70-15) TTG GAT AGT ACA GAA AGT ATT AAA AAC CCC GAC AGT TAT GCT ATT TTT GCA CAA TGT GCA [660]

#J1(O-20IN) TTG GAT AGT ACA GAA AGT ATT AAA AAC CCC GAC AGT TAT GCT ATT TTT GCA CAA TGT GCA [660]

#J1(O-29J) TTG GAT AGT ACA GAA AGT ATT AGA AAC CCC GAC AGT TAT GCT ATT TTT GCA CAA TGT GCA [660]

#J1(O-11J) TTG GAT AGT ACA GAA AGT ATT AGA AAC CCC GAC AGT TAT GCT ATT TTT GCA CAA TGT GCA [660]

#J1(O-2J) TTG GAT AGT ACA GAA AGT ATT AGA AAC CCC GAC AGT TAT GCT ATT TTT GCA CAA TGT GCA [660]

#J1(O-30C) TTG GAT AGT ACA GAA AGT ATT AAA AAC CCC GAC AGT TAT GCT ATT TTT GCA CAA TGT GCA [660]

#J1(O-5J) TTG GAT AGT ACA GAA AGT ATT AAA AAC CCC GAC AGT TAT GCT ATT TTT GCA CAA TGT GCA [660]

#J1(O-6J) TTG GAT AGT ACA GAA AGT ATT AAA AAC CCC GAC AGT TAT GCT ATT TTT GCA CAA TGT GCA [660]

#J2(O-11J) TTG GAT AGT ACA GAA AGT ATT AAA AAC CCC GAC AGT TAT GCT ATT TTT GCA CAA TGT GCA [660]

#J2(O-29J) TTG GAT AGT ACA GAA AGT ATT AAA AAC CCC GAC AGT TAT GCT ATT TTT GCA CAA TGT GCA [660]

#J3(O-5J) TTG GAT AGT ACA GAA AGT ATT AAA AAC CCC GAC AGT TAT GCT ATT TTT GCA CAA TGT GCA [660]

#J3(O-2J) TTG GAT AGT ACA GAA AGT ATT AAA AAC CCC GAC AGT TAT GCT ATT TTT GCA CAA TGT GCA [660]

#PO(O-23IN) TTG GAT AGT ACA GAA AGT ATT AAA AAC CCC GAC AGT TAT GCT ATT TTT GCA CAA TGT GCA [660]

#PO(O-20IN) TTG GAT AGT ACA GAA AGT ATT AAA AAC CCC GAC AGT TAT GCT ATT TTT GCA CAA TGT GCA [660]

#PO(4224-7-8) TTG GAT AGT ACA GAA AGT ATT AAA AAC CCC GAC AGT TAT GCT ATT TTT GCA CAA TGT GCA [660]

#Si(Si-5I) TTG GAT AGT ACA GAA AGT ATT AAA AAC CCC GAC AGT TAT GCT ATT TTT GCA CAA TGT GCA [660]

#Si(Si-6I) TTG GAT AGT ACA GAA AGT ATT AAA AAC CCC GAC AGT TAT GCT ATT TTT GCA CAA TGT GCA [660]

#Sv(Sv-7J) TTG GAT AGT ACA GAA AGT ATT AAA AAC CCC GAC AGT TAT GCT ATT TTT GCA CAA TGT GCA [660]

#Sv(Sv-8J) TTG GAT AGT ACA GAA AGT ATT AAA AAC CCC GAC AGT TAT GCT ATT TTT GCA CAA TGT GCA [660]

#Pm(Pm-1J) TTG GAT AGT ACA ACA AGT ATT AAA AAC CCC GAC AGC TAT GCT ATT TTT GCA CAA TGT GCA [660]

#Pm(Pm-2J) TTG GAT AGT ACA ACA AGT ATT AAA AAC CCC GAC AGC TAT GCT ATT TTT GCA CAA TGT GCA [660]

#Pm(Pm-3J) TTG GAT AGT ACA ACA AGT ATT AAA AAC CCC GAC AGC TAT GCT ATT TTT GCA CAA TGT GCA [660]

#Pm(Pm-4J) TTG GAT AGT ACA ACA AGT ATT AAA AAC CCC GAC AGC TAT GCT ATT TTT GCA CAA TGT GCA [660]

#Pm(Pm-5J) TTG GAT AGT ACA ACA AGT ATT AAA AAC CCC GAC AGC TAT GCT ATT TTT GCA CAA TGT GCA [660]

#D1(Dsa-1J) TTG GAT AGT ACA ACA AGT ATT AAA AAC CCC GAC AGC TAT GCT ATT TTT GCA CAA TGT GCA [660]

#D1(Dsa-3J) TTG GAT AGT ACA ACA AGT ATT AAA AAC CCC GAC AGC TAT GCT ATT TTT GCA CAA TGT GCA [660]

#D1(Dsm-4J) TTG GAT AGT ACA ACA AGT ATT AAA AAC CCC GAC AGC TAT GCT ATT TTT GCA CAA TGT GCA [660]

#D1(Dsa-2J) TTG GAT AGT ACA ACA AGT ATT AAA AAC CCC GAC AGC TAT GCT ATT TTT GCA CAA TGT GCA [660]

#D1(Dh-5B) TTG GAT AGT ACA ACA AGT ATT AAA AAC CCC GAC AGC TAT GCT ATT TTT GCA CAA TGT GCA [660]

#Ce1(Ce-1B) TTG GAT AGT ACA AAA AGT ATT AGA AAT GGC GAC AGC TAT GCT ATT TTT GCA CAA TGT GCA [660]

#Ce1(Ecc-1B) TTG GAT AGT ACA AAA AGT ATT AGA AAT GGC GAC AGC TAT GCT ATT TTT GCA CAA TGT GCA [660]

#DQ855958 TTG GAT AGT ACG AAA AGT ATC AAA AAT CCT GAC AGT TAT GTT ATC TTT GCA CAA TGT GCA [660]

#J1(70-15) CGT TAT AAA TAT TGT TAA --- [681]

#J1(O-20IN) CGT TAT AAA TAT TGT TAA --- [681]

#J1(O-29J) CGT TAT AAA TAT TGT TAA --- [681]

#J1(O-11J) CGT TAT AAA TAT TGT TAA --- [681]

#J1(O-2J) CGT TAT AAA TAT TGT TAA --- [681]

#J1(O-30C) CGT TAT AAA TAT TGT TAA --- [681]

#J1(O-5J) CGT TAT AAA TAT TGT TAA --- [681]

#J1(O-6J) CGT TAT AAA TAT TGT TAA --- [681]

#J2(O-11J) CGT TAT AAA TAT TGT TAA --- [681]

#J2(O-29J) CGT TAT AAA TAT TGT TAA --- [681]

#J3(O-5J) CGT TAT AAA TAT TGT TAA --- [681]

#J3(O-2J) CGT TAT AAA TAT TGT TAA --- [681]

#PO(O-23IN) CGT TAT AAA TAT TGT TAA --- [681]

#PO(O-20IN) CGT TAT AAA AAT TGT TAA --- [681]

#PO(4224-7-8) CGT TAT AAA TAT TGT TAA --- [681]

#Si(Si-5I) CGT TAT AAA TAT TGT TAA --- [681]

#Si(Si-6I) CGT TAT AAA TAT TGT TAA --- [681]

#Sv(Sv-7J) CGT TAT AAA TAT TGT TAA --- [681]

#Sv(Sv-8J) CGT TAT AAA TAT TGT TAA --- [681]

#Pm(Pm-1J) CGT TAT AAA TAT TGT TAA --- [681]

#Pm(Pm-2J) CGT TAT AAA TAT TGT TAA --- [681]

#Pm(Pm-3J) CGT TAT AAA TAT TGT TAA --- [681]

#Pm(Pm-4J) CGT TAT AAA TAT TGT TAA --- [681]

#Pm(Pm-5J) CGT TAT AAA TAT TGT TAA --- [681]

#D1(Dsa-1J) CGT TAT AAA TAT TGT TAA --- [681]

#D1(Dsa-3J) CGT TAT AAA TAT TGT TAA --- [681]

#D1(Dsm-4J) CGT TAT AAA TAT TGT TAA --- [681]

#D1(Dsa-2J) CGT TAT AAA AAT TGT TAA --- [681]

#D1(Dh-5B) CGT TAT AAA AAT TGT TAA --- [681]

#Ce1(Ce-1B) CGT TAT AAA ATC TGT TAA --- [681]

#Ce1(Ecc-1B) CGT TAT AAA ATC TGT TAA --- [681]

#DQ855958 CGA TAT GGA AGT TGT TAT TGA [681]
