# Supplementary material for: Multiple Translocation of the AVR-Pita Effector Gene among Chromosomes of the Rice Blast Fungus Magnaporthe oryzae and Related Species
Source: PLoS Pathog. 2011 Jul 28;7(7):e1002147. doi: 10.1371/journal.ppat.1002147 (PMC3145791; doi:10.1371/journal.ppat.1002147)
Supplement: Supplementary file 2 [file ppat.1002147.s004.doc]

Table S1. *Pyricularia* isolates used in this study.

Species	Code	Isolate[a]	Host	Locality	Isolation year	Collector or reference[b]	Genotype[c]
Pyricularia oryzae	O-1G	Guy11	*Oryza sativa*	Guiana (Cortin)	1978	J.L. Notteghem	Os1
	O-2J	Ken54-04 (MAFF235006)	*Oryza sativa*	Japan (Gifu)	1954	Kiyosawa (1974)	Os2
	O-3J	Ken54-20 (MAFF235005)	*Oryza sativa*	Japan (Yamaguchi)	1954	Kiyosawa (1974)	Os1
	O-4J	Hoku1	*Oryza sativa*	Japan (Hokkaido)	1948	Kiyosawa (1974)	Os1
	O-5J	Ina72 (MAFF235003)	*Oryza sativa*	Japan (Nagano)	1957	Kiyosawa (1974)	Os3
	O-6J	Ina168	*Oryza sativa*	Japan (Aichi)	1958	Kiyosawa (1974)	Os2
	O-7J	Ken53-33	*Oryza sativa*	Japan (Aichi)	1953	Kiyosawa (1974)	Os1
	O-8J	P-2b	*Oryza sativa*	Japan (Niigata)	1948	Kiyosawa (1974)	Os1
	O-9J	0903-4	*Oryza sativa*	Japan (Tochigi)	1976	Yamada et al. (1976)	Os1
	O-10J	2012-1	*Oryza sativa*	Japan (Ishikawa)	1976	Yamada et al. (1976)	Os1
	O-11J	2403-1	*Oryza sativa*	Japan (Mie)	1976	Yamada et al. (1976)	Os2
	O-12J	1836-3	*Oryza sativa*	Japan (Niigata)	1976	Yamada et al. (1976)	Os2
	O-13J	88A	*Oryza sativa*	Japan (Ibaraki)	1976	Yamada et al. (1976)	Os2
	O-14C	CHNOS 59-6-1	*Oryza sativa*	China (Yunnan)	1989	N. Hayashi	Os1
	O-15C	CHNOS 60-8-1	*Oryza sativa*	China (Yunnan)	1989	N. Hayashi	Os1
	O-16B	Br10	*Oryza sativa*	Brazil (Parana)	1990	S. Igarashi	Os2
	O-17B	Br13	*Oryza sativa*	Brazil (Parana)	1990	S. Igarashi	Os2
	O-18B	Br15	*Oryza sativa*	Brazil (Parana)	1990	S. Igarashi	Os1
	O-19B	Br18	*Oryza sativa*	Brazil (Parana)	1990	S. Igarashi	Os2
	O-20IN	PO-02-7306	*Oryza sativa*	Indonesia (Jawa Barat)	1973	A. Mukelar	Os4
	O-21IN	PO-02-7501	*Oryza sativa*	Indonesia (Jawa Barat)	1975	A. Mukelar	Os1
	O-22IN	PO-04-7501	*Oryza sativa*	Indonesia (Jawa Timur)	1975	A. Mukelar	Os1
	O-23IN	PO-12-7301-2	*Oryza sativa*	Indonesia (Lampung)	1973	A. Mukelar	Os1
	O-24IN	PO-12-7301	*Oryza sativa*	Indonesia (Lampung)	1973	A. Mukelar	Os1
	O-25V	VHT6.1	*Oryza sativa*	Vietnam (Ha Tay)	1998	L.D. Don	Os1
	O-26V	VTB6.1	*Oryza sativa*	Vietnam (Thai Binh)	1998	L.D. Don	Os1
	O-27V	VHG4.5	*Oryza sativa*	Vietnam (Hau Giang)	1996	L.D. Don	Os1
	O-28V	VHT3.3	*Oryza sativa*	Vietnam (Ha Tay)	1998	L.D. Don	Os1
	O-29J	84R-62B	*Oryza sativa*	Japan (Aichi)	1984	H. Naito	Os2
	O-30C	Y93-245c-2	*Oryza sativa*	China (Yunnan)	1993	Y. Fujita	Os5
	Si-1J	GFSI1-7-2	*Setaria italica*	Japan (Gifu)	1977	H. Kato	SP
	Si-2J	NRSI2-2-2	*Setaria italica*	Japan (Nara)	1977	H. Kato	SP
	Si-3J	NRSI3-1-1	*Setaria italica*	Japan (Nara)	1977	H. Kato	SP
	Si-4J	NNSI3-2-1	*Setaria italica*	Japan (Nagano)	1984	H. Kato	SP
	Si-5I	IN77-16-1-1	*Setaria italica*	India (Mysore)	1977	H. Kato	SP
	Si-6I	IN77-20-1-1	*Setaria italica*	India (Mysore)	1977	H. Kato	SP
	Sv-7J	KANSV1-4-1	*Setaria viridis*	Japan (Kanagawa)	1975	H. Kato	SP
	Sv-8J	N913	*Setaria viridis*	Japan (Chiba)	1974	N. Nishihara	SP
	Pm-1J	NNPM1-2-1	*Panicum miliaceum*	Japan (Nagano)	1984	N. Hayashi	SP
	Pm-2J	NRPM1-1-1	*Panicum miliaceum*	Japan (Nara)	1990	H. Kato	SP
	Pm-3J	STPM1-3-2	*Panicum miliaceum*	Japan (Saitama)	1981	H. Kato	SP
	Pm-4J	STPM4-2-2	*Panicum miliaceum*	Japan (Saitama)	1981	H. Kato	SP
	Pm-5J	SZPM1-1-1	*Panicum miliaceum*	Japan (Shizuoka)	1978	H. Kato	SP
	Pm-6J	YNPM4-1-1	*Panicum miliaceum*	Japan (Yamanashi)	1983	H. Kato	SP
	Pb-7J	N922	*Panicum bisulcatum*	Japan (Tochigi)	1974	N. Nishihara	SP
	Ec-1J	G10-1	*Eleusine coracana*	Japan (Tochigi)	1977	S. Itoi	TELE1
	Ec-2J	22-1	*Eleusine coracana*	Japan (Kagawa)	1977	S. Itoi	TELE1
	Ec-3J	MZ5-1-6	*Eleusine coracana*	Japan (Miyazaki)	1976	H. Kato	TELE1
	Ec-4J	Ken15-15-1	*Eleusine coracana*	Japan (Tokyo)	1976	H. Kato	TELE1
	Ec-5J	SZEC1-1-1	*Eleusine coracana*	Japan (Shizuoka)	1978	H. Kato	TELE2
	Ec-6J	GFEC1-5-1	*Eleusine coracana*	Japan (Gifu)	1977	H. Kato	TELE2
	Ec-7I	IN77-31-1-1	*Eleusine coracana*	India (Bangalor)	1977	H. Kato	TELE1
	Ec-8I	IN77-39-1-2	*Eleusine coracana*	India (Bangalor)	1977	H. Kato	TELE1
	Ec-9N	NP10-17-4-1-3	*Eleusine coracana*	Nepal (Syabru)	1975	S. Sakamoto	TELE1
	Ec-10N	NP10-28-1-1-1	*Eleusine coracana*	Nepal (Pokara)	1975	S. Sakamoto	TELE1
	Ea-11J	N1006	*Eleusine africana*	Japan (Tochigi)	1975	N. Nishihara	TELE1
	Eb-12J	N1011	*Eleusine boranensis*	Japan (Tochigi)	1975	N. Nishihara	TELE1
	Ei-13I	IN77-36-1-1	*Eleusine indica*	India (Mysore)	1977	H. Kato	TELE1
	A-1B	Br58	*Avena sativa*	Brazil (Parana)	1990	S. Igarashi	As
	T-1B	Br3	*Triticum aestivum*	Brazil (Parana)	1990	S. Igarashi	TELE3
	T-2B	Br7	*Triticum aestivum*	Brazil (Parana)	1990	S. Igarashi	TELE1
	T-3B	Br8	*Triticum aestivum*	Brazil (Parana)	1990	S. Igarashi	TELE3
	T-4B	Br48 (IMI368172)	*Triticum aestivum*	Brazil (Mato Grosso do Sul)	1990	S. Igarashi	TELE3
	T-5B	Br49 (IMI368173)	*Triticum aestivum*	Brazil (Mato Grosso do Sul)	1990	S. Igarashi	TELE1
	T-6B	Br115.7	*Triticum aestivum*	Brazil (Parana)	1992	A.S. Urashima	TELE1
	T-7B	Br116.5	*Triticum aestivum*	Brazil (Parana)	1992	A.S. Urashima	TELE1
	T-8B	Br118.2D	*Triticum aestivum*	Brazil (Parana)	1992	A.S. Urashima	TELE3
	L-1J	TP1	*Lolium perenne*	Japan (Tochigi)	1997	A. Tanaka, T. Tani	TELE1
	L-2J	TP2	*Lolium perenne*	Japan (Tochigi)	1997	A. Tanaka, T. Tani	TELE1
	L-3J	AK1	*Lolium perenne*	Japan (Akita)	1998	A. Tanaka, T. Tani	TELE1
	L-4J	LW3	*Lolium perenne*	Japan (Yamanashi)	1999	A. Tanaka, T. Tani	TELE1
	L-5J	F6	*Lolium perenne*	Japan (Chiba)	1998	A. Tanaka, T. Tani	TELE1
	L-6J	WK3-1	*Lolium perenne*	Japan (Yamaguchi)	1996	A. Tanaka, T. Tani	TELE1
	B-1B	Br35	*Brachiaria plantaginea*	Brazil (Parana)	1990	S. Igarashi	Bp1
	B-2B	Bp1a	*Brachiaria plantaginea*	Brazil (Sao Paulo)	1996	A.S. Urashima	Bp2
	Erl-1J	N986	*Eragrostis lehmanniana*	Japan (Kumamoto)	1975	N. Nishihara	TELE2
Pyricularia sp. (LS)[d]	Leo-1J	N919 (MAFF305509)	*Leersia oryzoides*	Japan (Chiba)	1974	N. Nishihara	Lo
	Sg-1B	Br37	*Setaria geniculata*	Brazil (Parana)	1990	S. Igarashi	Sg
Pyricularia sp. (CE)[d]	Cc-1J	N981 (MAFF305501)	*Cenchrus ciliaris*	Japan (Kumamoto)	1975	N. Nishihara	CE1
	Ce-1B	Br36	*Cenchrus echinatus*	Brazil (Parana)	1990	S. Igarashi	CE2
	Ecc-1B	Br38	*Echinochloa colonum*	Brazil (Parana)	1990	S. Igarashi	CE2
Pyricularia grisea	Dsa-1J	Dig41	*Digitaria sanguinalis*	Japan (Hyogo)	1990	Y. Iwamoto	Dsah1
	Dsa-2J	N907	*Digitaria sanguinalis*	Japan (Tochigi)	1974	N. Nishihara	Dsah2
	Dsa-3J	IBDS4-1-1	*Digitaria sanguinalis*	Japan (Ibaraki)	1985	N. Hayashi	Dsah1
	Dsm-4J	N980	*Digitaria smutsii*	Japan (Kumamoto)	1975	N. Nishihara	Dsah2
	Dh-5B	Br29 (IMI368175)	*Digitaria horizontalis*	Brazil (Sao Paulo)	1990	S. Igarashi	Dsah2
	Dh-6B	Br33	*Digitaria horizontalis*	Brazil (Parana)	1990	S. Igarashi	Dsah3
Pyricularia zizaniaecola	Zz-1J	IBZL3-1-1	*Zizania latifolia*	Japan (Ibaraki)	1985	N. Hayashi	Zl1
	Zz-2J	KYZL201-1-1	*Zizania latifolia*	Japan (Kyoto)	2003	K. Yoshida, K. Hirata	Zl2
Pyricularia zingiberi	Z-1J	HYZiM101-1-1-1	*Zingiber mioga*	Japan (Hyogo)	1990	M. Ogawa	Zm1
	Z-2J	HYZiM201-0-1	*Zingiber mioga*	Japan (Hyogo)	2002	H. Kato	Zm2
	Z-3J	HYZiM202-1-2	*Zingiber mioga*	Japan (Hyogo)	2003	I. Chuma	Zm3
	Z-4J	HYZiM201-1-1	*Zingiber mioga*	Japan (Hyogo)	2003	I. Chuma	Zm4
Pyricularia sp. (SsPh)[d]	Ss-1J	INA-B-92-45	*Sasa* sp.	Japan (Aichi)	1992	S. Koizumi	Ss
	Ph-1J	INA-B-93-19	*Phyllostachys bambusoides*	Japan (Aichi)	1993	S. Koizumi	Ph
Pyricularia sp. (Kb)[d]	K-1J	HYKB202-1-2	*Kyllinga brevifolia*	Japan (Hyogo)	2003	I. Chuma	Kb1
	K-2J	FKKB201-1-5	*Kyllinga brevifolia*	Japan (Fukuoka)	2003	I. Chuma	Kb2
	K-3J	FKKB201-3-2	*Kyllinga brevifolia*	Japan (Fukuoka)	2003	I. Chuma	Kb2
Pyricularia higginsii	Ci-1J	HYCI201-1-1	*Cyperus iria*	Japan (Hyogo)	2002	H. Kato	Ci

[a] Accession numbers in public culture collections are shown in parentheses. (MAFF, Microorganisms Section of the NIAS Genebank, National Institute of Agrobiological Sciences, Tsukuba, Japan; IMI, CABI Bioscience, UK Centre, Egham, UK)

[b] Kiyosawa, S. (1974). Studies on genetics and breeding of blast resistance in rice. Misc. Publ. Bull. Natl. Inst. Agric. Sci. D No.1: 1-58 (in Japanese with English summary).

[c] Yamada, M., Kiyosawa, S., Yamaguchi, T., Hirano, T., Kobayashi, T., Kushibuchi, K., and Watanabe, S. (1976). Proposal of a new method for differentiating races of *Pyricularia oryzae* Cavara in Japan. Ann. Phytopathol. Soc. Jpn. 42: 216-219.

[d] Refer to Hirata et al. [12]
