# Supplementary material for: Multiple Translocation of the AVR-Pita Effector Gene among Chromosomes of the Rice Blast Fungus Magnaporthe oryzae and Related Species
Source: PLoS Pathog. 2011 Jul 28;7(7):e1002147. doi: 10.1371/journal.ppat.1002147 (PMC3145791; doi:10.1371/journal.ppat.1002147)
Supplement: Supplementary file 3 [file ppat.1002147.s005.doc]

Table S2. *AVR-Pita* clones used in this study

				Insert		
Clone	Accession No.	Vector	Cloning site	RFLP type	Origin	Description
pTA-O2-1	-	pBluescript II SK+	*Eco* RV	J1	O-2J	1.8 kb PCR fragment amplified from a O-2J 6.5 kb *Eco* RI fragment with AVRPita-O11-25 and AVRPita-O11-26
pTA-O2-2	AB607337	pBluescript II SK+	*Eco* RV	J3	O-2J	1.8 kb PCR fragment amplified from a O-2J 8.2 kb *Eco* RI fragment with AVRPita-O11-25 and AVRPita-O11-26
pTA-O5-2	-	pBluescript II SK+	*Eco* RV	J1	O-5J	1.8 kb PCR fragment amplified from a O-5J 6.5 kb *Eco* RI fragment with AVRPita-O11-25 and AVRPita-O11-26
pTA-O5-4	-	pBluescript II SK+	*Eco* RV	J3	O-5J	1.8 kb PCR fragment amplified from a O-5J 5.0 kb *Eco* RI fragment with AVRPita-O11-25 and AVRPita-O11-26
pP4-1	-	pBluescript II SK+	*Eco* RV	J3	O-5J	766 bp PCR fragment amplified from O-5J total DNA with AVRPita-ORF-F2 and AVRPita-ORF-R2
pTA-OU1-1-2	-	pMLF2	*Bam* HI	J1	O-11J	33.4 kb fragment from a O-11J cosmid library
pTA-O11-1*	-	pBluescript II SK+	*Eco* RV	J1	O-11J	1.8 kb PCR fragment amplified from O-11J total DNA with AVRPita-O11-25 and AVRPita-O11-26
pTA-O11-2	-	pBluescript II SK+	*Eco* RV	J2	O-11J	1.8 kb PCR fragment amplified from a O-11J >20 kb *Eco* RI fragment with AVRPita-O11-25 and AVRPita-O11-26
pTA-O29-F39*	AB607334	pCC1FOS	*Pma* CI	J1	O-29J	38.9 kb fragment from a O-29J fosmid library
pTA-O29-F8*	AB607336	pCC1FOS	*Pma* CI	J2	O-29J	39.9 kb fragment from a O-29J fosmid library
pTA-O30-28*	AB607335	pBluescript II SK+	*Eco* RV	J1	O-30C	1.8 kb PCR fragment amplified from O-30C total DNA with AVRPita-O11-25 and AVRPita-O11-26
pTA-O30-F8	AB607346	pCC1FOS	*Pma* CI	CH	O-30C	≈35 kb fragment from a O-30C fosmid library
pTA-O20-7*	AB607333	pBluescript II SK+	*Eco* RV	J1	O-20IN	1.8 kb PCR fragment amplified from O-20N total DNA with AVRPita-O11-25 and AVRPita-O11-26
pTA-O20-12*	AB607339	pBluescript II SK+	*Eco* RV	PO	O-20IN	2.1 kb PCR fragment amplified from O-20N total DNA with AVRPita-O23-6 and AVRPita-O23-7
pTA-O23-17*	-	pBluescript II SK+	*Eco* RV	PO	O-23IN	2.1 kb PCR fragment amplified from O-23N total DNA with AVRPita-O23-6 and AVRPita-O23-7
pTA-O23-AclI2746	AB607338	pBluescript II SK+	*Acl* I	PO	O-23IN	2.7 kb *Acl* I fragment from O-23IN total DNA
pTA-O23-tel3	AB607338	pBluescript II SK+	*Eco* RV	PO	O-23IN	0.9 kb PCR fragment amplified from O-23N total DNA with AVRPita-ORF-F2 and Tel-CCCTAAx4
pTA-S6-F3-3	AB607340	pCC1FOS	*Pma* CI	Si	Si-6I	37.4 kb fragment from a Si-6I fosmid library
pTA-S6-P4.6-1*	-	pBluescript II SK+	*Pst* I	Si	Si-6I	4.6 kb *Pst* I fragment from Si-6I total DNA
pTA-S7-A3.9-1	AB607341	pBluescript II SK+	*Acl* I	Sv	Sv-7J	3.9 kb *Acl* I fragment from Sv-7J total DNA
pTA-S7-H4.0-1-1	AB607341	pBluescript II SK+	*Hin* dIII	Sv	Sv-7J	4.0 kb *Hin*dIII fragment from Sv-7J total DNA
pTA-S7-3-22*	-	pBluescript II SK+	*Eco* RV	Sv	Sv-7J	1.5 kb PCR fragment amplified from Sv-7J total DNA with AVRPita-S7-6 and AVRPita-S7-7
pTA-P1-F5-2	AB607342	pCC1FOS	*Pma* CI	Pm	Pm-1J	36.4 kb fragment from a Pm-1J fosmid library
pTA-P1-B5.5-5*	-	pBluescript II SK+	*Bam* HI	Pm	Pm-1J	5.5 kb *Bam* HI fragment from Pm-1J total DNA
pTA-P2-10	-	pBluescript II SK+	*Eco* RV	Pm	Pm-2J	2.3 kb PCR fragment amplified from Pm-2J total DNA with AVRPita-P1-20 and AVRPita-P1-2
pTA-P3-17	-	pBluescript II SK+	*Eco* RV	Pm	Pm-3J	2.3 kb PCR fragment amplified from Pm-3J total DNA with AVRPita-P1-20 and AVRPita-P1-2
pTA-P4-27	-	pBluescript II SK+	*Eco* RV	Pm	Pm-4J	2.3 kb PCR fragment amplified from Pm-4J total DNA with AVRPita-P1-20 and AVRPita-P1-2
pTA-P5-33	-	pBluescript II SK+	*Eco* RV	Pm	Pm-5J	2.3 kb PCR fragment amplified from Pm-5J total DNA with AVRPita-P1-20 and AVRPita-P1-2
pTA-D1-F5-1	AB607343	pCC1FOS	*Pma* CI	D1	Dsa-1J	39.9 kb fragment from a Dsa-1J fosmid library
pTA-D1-E3.6-1*	-	pBluescript II SK+	*Eco* RI	D1	Dsa-1J	3.6 kb *Eco* RI fragment from Dsa-1J total DNA
pTA-D2-52	-	pBluescript II SK+	*Eco* RV	D1	Dsa-2J	1.6 kb PCR fragment amplified from Dsa-2J total DNA with AVRPita-P1-21 and AVRPita-D1-5
pTA-D3-58	-	pBluescript II SK+	*Eco* RV	D1	Dsa-3J	1.6 kb PCR fragment amplified from Dsa-3J total DNA with AVRPita-P1-21 and AVRPita-D1-5
pTA-D4-65	-	pBluescript II SK+	*Eco* RV	D1	Dsm-4J	1.6 kb PCR fragment amplified from Dsm-4J total DNA with AVRPita-P1-21 and AVRPita-D1-5
pTA-D5-75*	AB607344	pBluescript II SK+	*Eco* RV	D1	Dh-5B	1.6 kb PCR fragment amplified from Dh-5B total DNA with AVRPita-P1-21 and AVRPita-D1-5
pTA-X4-A2.1-1-1	AB607345	pBluescript II SK+	*Acl* I	Ce1	Ce-1B	2.1 kb *Acl* I fragment from Ce-1B total DNA
pTA-X4-H4.1-2	AB607345	pBluescript II SK+	*Hin* dIII	Ce1	Ce-1B	4.1 kb *Hin*dIII fragment from Ce-1B total DNA
pTA-X4-5-32*	-	pBluescript II SK+	*Eco* RV	Ce1	Ce-1B	2.1 kb PCR fragment amplified from Ce-1B total DNA with AVRPita-X4-9 and AVRPita-X4-11
pTA-Br38-36	-	pBluescript II SK+	*Eco* RV	Ce1	Ecc-1B	1.5 kb PCR fragment amplified from Ecc-1B total DNA with AVRPita-X4-10 and AVRPita-X4-11

*, Clones used for the functional analysis through transformation
