# Supplementary material for: Multiple Translocation of the AVR-Pita Effector Gene among Chromosomes of the Rice Blast Fungus Magnaporthe oryzae and Related Species
Source: PLoS Pathog. 2011 Jul 28;7(7):e1002147. doi: 10.1371/journal.ppat.1002147 (PMC3145791; doi:10.1371/journal.ppat.1002147)
Supplement: Supplementary file 4 [file ppat.1002147.s006.doc]

Table S3. Primers used in this study

Target gene or region	Primer name	Primer sequence (5' - 3')	Tm (C)	Size (bp)	Template	Polymerase used for amplification	Purpose
AVR-Pita (APita766)	AVRPita-ORF-F2	TTACGATTACAATCCAATTCCAAACCATAT	67.64	766	Genomic DNA of O-5J, pR4-1	KOD+ (TOYOBO, Osaka)	Hybridization probe
	AVRPita-ORF-R2	TAGCATAACTGTCGGGGTTTTTAATACTTT	66.48				
AVR-Pita	AVRPita-ORF-F2	TTACGATTACAATCCAATTCCAAACCATAT	67.64	801	Fosmid clone pTA-O29-F8	KOD+ (TOYOBO, Osaka)	Sequencing
	AVRPita-ORF-R1	TTAACAATATTTATAACGTGCACATTGTGC	66.50				
AVR-Pita3 (APita3-958)	AVRPita-G22-2	GTTTTTATTATATTTACAACTGGA	52.91	958	Genomic DNA of B-13I	KOD+ (TOYOBO, Osaka)	Hybridization probe
	AVRPita-G22-3	AAAAGGTAGCTCAATAACAA	53.20				
MGG_10916	AVRPita-S6-16	ATGGGCAAGTTCTTCTTCG	61.75	1766	Fosmid clone pTA-S6-F3-3	KOD+ (TOYOBO, Osaka)	Hybridization probe
	AVRPita-S6-17	TTTCGCTTTAACCGCTG	60.09				
AVR-Pita J1 (O-29J)	AVRPita-O23-3	AATCGACCCGTTTCCGCC	69.30	1019	Genomic DNA of O-29J, O-30C and their F_1	HotStarTaq (QIAGEN, Hiden)	Detection of single nucleotide polymorphism
	AVRPita-O29-5	AGCATAACTGTCGGGGTTTC	62.77				
AVR-Pita J1, J2, J3	AVRPita-O11-25	TACAAATCGCTTCGGTTCAG	62.67	1841	Genomic DNA of isolates carrying J1, J2, J3	KOD+ (TOYOBO, Osaka)	Cloning, sequencing, transformation
	AVRPita-O11-26	TCACGTGCAACTGGCTTG	65.02				
AVR-Pita PO	AVRPita-O23-6	TGGTGGGGCTTTTTAATTTC	62.42	2066	Genomic DNA of isolates carrying PO	KOD+ (TOYOBO, Osaka)	Cloning, sequencing, transformation
	AVRPita-O23-7	CTCTATTGTTAGATTGACCGCG	62.37				
AVR-Pita PO telomere	AVRPita-ORF-F2	TTACGATTACAATCCAATTCCAAACCATAT	67.64	880	Genomic DNA of O-23IN	KOD FX (TOYOBO, Osaka)	Cloning, sequencing
	Tel-CCCTAAx4	CCCTAACCCTAACCCTAACCCTAA	64.71				
AVR-Pita Si	AVRPita-S6-14	ACGATAATCTCTGCACGCCG	67.28	1938	Genomic DNA of Si-5I	KOD+ (TOYOBO, Osaka)	Direct sequencing
	AVRPita-S6-15	TAACACGTGCAACTGGCTTG	64.82				
AVR-Pita Sv	AVRPita-S7-6	CGTGAGGTGGAGCTAGTC	58.41	1460	Genomic DNA of Sv-7J	KOD+ (TOYOBO, Osaka)	Cloning, sequencing, transformation
	AVRPita-S7-7	GGCCGTGACAATTTATCCCT	64.70				
AVR-Pita Sv	AVRPita-S7-14	AGTTGAACAAAGCTGCACGAC	64.27	1737	Genomic DNA of Sv-8J	KOD+ (TOYOBO, Osaka)	Direct sequencing
	AVRPita-S7-7	GGCCGTGACAATTTATCCCT	64.70				
AVR-Pita Pm	AVRPita-P1-20	AATGTTCAATGAAGGAAAAGG	59.43	2309	Genomic DNA of isolates carrying Pm	KOD+ (TOYOBO, Osaka)	Cloning, sequencing, transformation
	AVRPita-P1-2	TGCCTGGCCGTAACAATTCC	68.94				
AVR-Pita D1	AVRPita-P1-21	CCTGGCCGTGACAGTTAC	61.81	1603	Genomic DNA of isolates carrying D1	KOD+ (TOYOBO, Osaka)	Cloning, sequencing, transformation
	AVRPita-D1-5	TGCCTGGCCGTGACAATTCC	72.55				
AVR-Pita Ce1	AVRPita-X4-9	TACTACGTGCGAATAGTACG	55.56	2144	Genomic DNA of Ce-1B	KOD+ (TOYOBO, Osaka)	Cloning, sequencing, transformation
	AVRPita-X4-11	AAGGTGAACGTGGCTGTAC	60.18				
AVR-Pita Ce1	AVRPita-X4-10	AAAGTTAATGCCTCGTACAC	55.83	1453	Genomic DNA of Ecc-1B	KOD+ (TOYOBO, Osaka)	Cloning, sequencing
	AVRPita-X4-11	AAGGTGAACGTGGCTGTAC	60.18				
rDNA in GFSI1-7-2	ITS_rd5*	CCGAGGTCAACCTTTAAAA	59.13	516	Genomic DNA of Si-1J, T-4B, their F_1	HotStarTaq (QIAGEN, Hiden)	Detection of single nucleotide polymorphism
	ITS5**	GGAAGTAAAAGTCGTAACAAGG	58.34				
rDNA in Br48	ITS_rd6*	CCGAGGTCAACCTTTAAAG	58.74	516	Genomic DNA of Si-1J, T-4B, their F_1	HotStarTaq (QIAGEN, Hiden)	Detection of single nucleotide polymorphism
	ITS5**	GGAAGTAAAAGTCGTAACAAGG	58.34				

*Chuma I. et al. [51]

**White T. J. et al. (1990) Amplification and direct sequencing of fungal ribosomal RNA genes for phylogenetics. Pages. 315-322 in: PCR Protocols: A Guide to Methods and Applications. M. A. Innis, D. H. Gelfand, J. J. Sninsky, and T. J. White, eds. Academ
