# Supplementary material for: Multiple Translocation of the AVR-Pita Effector Gene among Chromosomes of the Rice Blast Fungus Magnaporthe oryzae and Related Species
Source: PLoS Pathog. 2011 Jul 28;7(7):e1002147. doi: 10.1371/journal.ppat.1002147 (PMC3145791; doi:10.1371/journal.ppat.1002147)
Supplement: Supplementary file 5 [file ppat.1002147.s007.doc]

Table S4. Genetic markers used in this study

**Si-1J x T-4B**	
**Chromosome 1**	
Marker	Description
Tel8r-st	RFLP identified with telomere oligonucleotide $(TTAGGG)_{10}$
2-9-G	RFLP marker described by Nitta et al. [47]
lk0804-5s	AFLP
T1-C9	RFLP identified with cosmid clone T1-C9 derived from T-4B
T1-F10	RFLP identified with cosmid clone T1-F10 derived from T-4B
lk0704-3st	AFLP
11	RFLP marker described by Nitta et al. [47]
lk0410-2t	AFLP
lk0509-4s	AFLP
G34R	RFLP marker described by Nitta et al. [47]
MGM423ag	SSR described by Zheng et al. [52]
T1-F9-2	RFLP identified with cosmid clone T1-F9 derived from T-4B
lk0410-1s	AFLP
lk0506-3t	AFLP
lk0312-1s	AFLP
T1-G5	RFLP identified with cosmid clone T1-G5 derived from T-4B
AVR1-CO39	RFLP identified with the 1.05-kb *AVR1-CO39* fragment described by Farman et al. (2002)*
T1-A6	RFLP identified with cosmid clone T1-A6 derived from T-4B
5-10-F	RFLP marker described by Nitta et al. [47]
T1-F3-2	RFLP identified with cosmid clone T1-F3 derived from T-4B
MGM35	SSR described by Zheng et al. [52]
Tel14r-s	RFLP identified with telomere oligonucleotide $(TTAGGG)_{10}$
**Chromosome 2**	
Marker	Description
Pwt1	Avirulence gene locus determined in an infection assay [51]
rDNA	SNP identified with ITS_rd5 / ITS_rd6 and ITS5
36-7-E-E3.7-t	RFLP marker described by Nitta et al. [47]
35-12-A	RFLP marker described by Nitta et al. [47]
A15C10	RFLP marker described by Nitta et al. [47]
lk0804-8s	AFLP
T-U2-E4.2-t	RFLP identified with a cosmid clone T-U2 derived from T-4B
16-1-B	RFLP marker described by Nitta et al. [47]
T1-D10	RFLP identified with cosmid clone T1-D10 derived from T-4B
lk0712-2t	AFLP
lk0212-2t	AFLP
T1-D5-1	RFLP identified with cosmid clone T1-D5 derived from T-4B
T1-H2	RFLP identified with cosmid clone T1-H2 derived from T-4B
13-4-A	RFLP marker described with Nitta et al. [47]
lk0506-5st	AFLP
CH2-90H	RFLP marker described by Nitta et al. [47]
T1-B2	RFLP identified with cosmid clone T1-B2 derived from T-4B
T1-A2	RFLP identified with cosmid clone T1-A2 derived from T-4B
4-78	RFLP marker described by Nitta et al. [47]
T1-F12	RFLP identified with cosmid clone T1-F12 derived from T-4B
Tel2r-s	RFLP identified with telomere oligonucleotide $(TTAGGG)_{10}$

Chromosome 3	
Marker	Description
Tel3r-st	RFLP identified with telomere oligonucleotide $(TTAGGG)_{10}$
lk0506-1t	AFLP
lk0212-1s	AFLP
lk0212-3st	AFLP
lk0712-3st	AFLP
lk0804-4s	AFLP
lk0704-5s	AFLP
lk0410-6s	AFLP
T1-F3-1	RFLP identified with cosmid clone T1-F3 derived from T-4B
T1-D4	RFLP identified with cosmid clone T1-D4 derived from T-4B
lk0312-2t	AFLP
T1-F3-3	RFLP identified with cosmid clone T1-F3 derived from T-4B
T1-D8-1	RFLP identified with cosmid clone T1-D8 derived from T-4B
lk0509-8t	AFLP
lk0704-4t	AFLP
T1-F6	RFLP identified with cosmid clone T1-F6 derived from T-4B
T1-H4	RFLP identified with cosmid clone T1-H4 derived from T-4B
T1-H5	RFLP identified with cosmid clone T1-H5 derived from T-4B
T1-F11	RFLP identified with cosmid clone T1-F11 derived from T-4B
lk0804-11s	AFLP
CH5-176H	RFLP marker described by Nitta et al. [47]
lk0509-7t	AFLP
T1-D6	RFLP identified with cosmid clone T1-D6 derived from T-4B
T1-D11	RFLP identified with cosmid clone T1-D11 derived from T-4B
T1-C11	RFLP identified with cosmid clone T1-C11 derived from T-4B
T1-D8-3	RFLP identified with cosmid clone T1-D8 derived from T-4B
lk0312-3t	AFLP
T1-D8-2	RFLP identified with cosmid clone T1-D8 derived from T-4B
Tel7r-st	RFLP identified with telomere oligonucleotide $(TTAGGG)_{10}$

Chromosome 4	
Marker	Description
Tel5r-s	RFLP identified with telomere oligonucleotide $(TTAGGG)_{10}$
lk0804-7t	AFLP
55	RFLP marker described by Nitta et al. [47]
T1-E3-2	RFLP identified with cosmid clone T1-E3 derived from T-4B
lk0509-6t	AFLP
T1-A3	RFLP identified with cosmid clone T1-A3 derived from T-4B
T1-B3	RFLP identified with cosmid clone T1-B3 derived from T-4B
4-14	RFLP marker described by Nitta et al. [47]
T1-C4	RFLP identified with cosmid clone T1-C4 derived from T-4B
lk1103-2s	AFLP
T1-E3-1	RFLP identified with cosmid clone T1-E3 derived from T-4B
T1-C2	RFLP identified with cosmid clone T1-C2 derived from T-4B
lk0509-3t	AFLP
lk0804-1s	AFLP
T-U2-E5.5-s	RFLP identified with a cosmid clone T-U2 derived from T-4B
T1-E8	RFLP identified with cosmid clone T1-E8 derived from T-4B
T1-D1	RFLP identified with cosmid clone T1-D1 derived from T-4B
T1-F2	RFLP identified with cosmid clone T1-F2 derived from T-4B
T1-H3	RFLP identified with cosmid clone T1-H3 derived from T-4B
lk0509-9st	AFLP
42-10-C	RFLP marker described by Nitta et al. [47]
T1-G6	RFLP identified with cosmid clone T1-G6 derived from T-4B
T1-G3	RFLP identified with cosmid clone T1-G3 derived from T-4B
lk0410-5s	AFLP
lk0804-12s	AFLP
lk1103-7s	AFLP
lk0509-1t	AFLP
CH3-67H	RFLP marker described by Nitta et al. [47]
Tel4r-s	RFLP identified with telomere oligonucleotide $(TTAGGG)_{10}$

Chromosome 5	
Marker	Description
Tel13-st	RFLP identified w ith telomere oligonucleotide (TTAGGG)$_{10}$
28-6-E	RFLP marker described by Nitta et al. [47]
T1-D7	RFLP identified w ith cosmid clone T1-D7 derived from T-4B
Ik1103-3s	AFLP
T1-F8	RFLP identified w ith cosmid clone T1-F8 derived from T-4B
Ik0701-2t	AFLP
Ik0704-7s	AFLP
MGM253ag	SSR described by Zheng et al. [52]
T1-D5	RFLP identified w ith cosmid clone T1-D5 derived from T-4B
19-10-A	RFLP marker described by Nitta et al. [47]
T1-G10	RFLP identified w ith cosmid clone T1-G10 derived from T-4B
T1-H1	RFLP identified w ith cosmid clone T1-H1 derived from T-4B
MGM171ag	SSR described by Zheng et al. [52]
Ik0410-7t	AFLP
T1-F7	RFLP identified w ith cosmid clone T1-F7 derived from T-4B
MGM73ag	SSR described by Zheng et al. [52]
Ik0410-9st	AFLP
T1-G1	RFLP identified w ith cosmid clone T1-G1 derived from T-4B
4-178	RFLP marker described by Nitta et al. [47]
T1-E6	RFLP identified w ith cosmid clone T1-E6 derived from T-4B
CH3-12H	RFLP marker described by Nitta et al. [47]
Tel1r-st	RFLP identified w ith telomere oligonucleotide (TTAGGG)$_{10}$

Chromosome 6	
Marker	Description
Tel9r-st	RFLP identified w ith telomere oligonucleotide (TTAGGG)$_{10}$
MGG10916-st	RFLP identified w ith *AVR-Pita* -flanking gene MGG_10916 (amplified w ith AVRPita-S6-16 and AVRPita-S6-17)
T1-B1	RFLP identified w ith cosmid clone T1-B1 derived from T-4B
T1-G4	RFLP identified w ith cosmid clone T1-G4 derived from T-4B
T1-C6-1	RFLP identified w ith cosmid clone T1-C6 derived from T-4B
T1-E11	RFLP identified w ith cosmid clone T1-E11 derived from T-4B
Ik0212-5t	AFLP
T1-G12	RFLP identified w ith cosmid clone T1-G12 derived from T-4B
T1-A1	RFLP identified w ith cosmid clone T1-A1 derived from T-4B
CH4-121H	RFLP marker described by Nitta et al. [47]
Ik0410-8st	AFLP
Ik0410-4s	AFLP
Ik0509-5st	AFLP
T1-F4	RFLP identified by cosmid clone T1-F4 derived from T-4B
CH2-57H	RFLP marker described by Nitta et al. [47]
CH3-122H	RFLP marker described by Nitta et al. [47]
Ik0704-2st	AFLP
Ik0704-8t	AFLP
Ik1103-4s	AFLP
T1-B5	RFLP identified w ith cosmid clone T1-B5 derived from T-4B
T1-C10	RFLP identified w ith cosmid clone T1-C10 derived from T-4B
Ik1103-6s	AFLP
Tel15r-s	RFLP identified w ith telomere oligonucleotide (TTAGGG)$_{10}$

Chromosome 7	
Marker	Description
Tel6r-st	RFLP identified with telomere oligonucleotide $(TTAGGG)_{10}$
G131R	RFLP marker described by Nitta et al. [47]
T1-E5	RFLP identified with cosmid clone T1-E5 derived from T-4B
CH5-75H	RFLP marker described by Nitta et al. [47]
MGM282ag	SSR described by Zheng et al. [52]
T1-C8	RFLP identified with cosmid clone T1-C8 derived from T-4B
CH2-32H	RFLP marker described by Nitta et al. [47]
lk1103-9s	AFLP
T1-A11	RFLP identified with cosmid clone T1-A11 derived from T-4B
T1-E1	RFLP identified with cosmid clone T1-E1 derived from T-4B
lk0312-4t	AFLP
AVR-Pita3	RFLP identified with *AVR-Pita3* (amplified with AVRPita-G22-2 and AVRPita-G22-3)
MGM302ag	SSR described by Zheng et al. [52]
Tel10r-st	RFLP identified with telomere oligonucleotide $(TTAGGG)_{10}$
**Si-6I x T-7B**	
**Chromosome 6**	
Marker	Description
Tel-Sc-4.1s	RFLP identified with telomere oligonucleotide $(TTAGGG)_{10}$
AVR-Pita-Si (Si-6I)	RFLP identified with *AVR-Pita* probe APita801 (amplified with AVRPita-ORF-F2 and AVRPita-ORF-R1)
MGG10916-st	RFLP identified with *AVR-Pita*-flanking gene MGG_10916 (amplified with AVRPita-S6-16 and AVRPita-S6-17)
MGG11075-st	RFLP identified with *AVR-Pita*-flanking gene MGG_11075 (amplified with AVRPita-S6-26 and AVRPita-S6-27)
MGG10771-st	RFLP identified with *AVR-Pita*-flanking gene MGG_10771 (amplified with AVRPita-S6-30 and AVRPita-S6-31)
CO39-4s	RFLP identified as a weak signal with the *AVR1-CO39* 1.05-kb fragment described by Farman et al. (2002)*
T1-B1-st	RFLP identified with cosmid clone T1-B1 derived from T-4B
1003-150s	AFLP
MGG13755-2st	RFLP identified with *AVR-Pita*-flanking gene MGG_13755 (amplified with AVRPita-O11-19 and AVRPita-O11-20)
0108-1400s	AFLP
0108-330s	AFLP
0404-280s	AFLP
0211-1000s	AFLP
0207-190s	AFLP
0207-310s	AFLP
CH4-121H-st	RFLP marker described by Nitta et al. [47]
1003-420s	AFLP
1003-440t	AFLP
0211-1800t	AFLP
CH2-57H-st	RFLP marker described by Nitta et al. [47]
CH3-122H-1s	RFLP marker described by Nitta et al. [47]
T1-F4-S-st	RFLP identified with a cosmid clone T1-F4 derived from T-4B
0106-360s	AFLP
0106-380t	AFLP
CO39-5s	RFLP identified as a weak signal with the *AVR1-CO39* 1.05-kb fragment described by Farman et al. (2002)*
Tel-S-5.2s	RFLP identified with telomere oligonucleotide $(TTAGGG)_{10}$

*Farman, M.L., Eto, Y., Nakao, T., Tosa, Y., Nakayashiki, H., Mayama, S., Leong, S.A. (2002). Analysis of the structure of the *AVR1-CO39* avirulence locus in virulent rice-infecting isolates of *Magnaporthe grisea*. Mol. Plant-Microbe Interact. 15: 6-16.
